# LINC01116, a hypoxia‐lncRNA marker of pathological lymphangiogenesis and poor prognosis in lung adenocarcinoma

**DOI:** 10.1002/1878-0261.70175

**Published:** 2025-12-09

**Authors:** Marine Gautier‐Isola, Rafael Lopes Goncalves, Marin Truchi, Caroline Lacoux, Célia Scribe, Hugo Cadis, Laetitia Guardini, Sophie Bekisz, Marius Ilié, Paul Hofman, Georges Vassaux, Bernard Mari, Roger Rezzonico

**Affiliations:** ^1^ UMR CNRS 7275, INSERM 1323, IPMC, IHU RespirERA Valbonne Université Côte d'Azur France; ^2^ Laboratory of Biology of Tumor and Development, GIGA‐Cancer Liege University Belgium; ^3^ Laboratory of Clinical and Experimental Pathology and Hospital‐Integrated Biobank (BB‐0033‐00025), CHU Nice, IHU RespirERA Université Côte d'Azur France

**Keywords:** biomarker, hypoxia, long noncoding RNA, lung adenocarcinoma, lymphangiogenesis

## Abstract

A hypoxic microenvironment promotes the aggressiveness of lung adenocarcinoma (LUAD) through treatment resistance and generation of new lymphatic vessels (i.e., lymphangiogenesis) favoring metastatic dissemination. Transcriptomic analysis of cohorts of LUAD patients highlighted LINC01116, a long noncoding RNA, associated with a bad prognosis, a high rate of recurrence, and induced by hypoxia in tumors. Gain‐ (overexpression) and loss‐of‐function (CRISPRi (Clustered Regularly Interspaced Short Palindromic Repeats interference, RNA interference)) approaches performed in LUAD cancer cell lines did not reveal a clear regulatory role for LINC01116 in tumor cells. Analyses of LUAD single‐cell RNA sequencing data sets and RNA Fluorescent *In Situ* Hybridization (RNA‐FISH) showed high expression of LINC01116 in lymphatic endothelial cells (LEC) pointing to this transcript as a specific biomarker of tumoral lymphangiogenesis. Efficient knockdown of LINC01116 in LEC in normoxic or hypoxic conditions impacted the proliferation rate under hypoxic stimulation and revealed a gene signature associated with proliferation and hypoxia sensing. Together, our data suggest a role for LINC01116 in pathological lymphangiogenesis of lung tumors.

## Graphical abstract

The LINC01116 long noncoding RNA is induced by hypoxia and associated with poor prognosis and high recurrence rates in two cohorts of lung adenocarcinoma patients. Here, we demonstrate that besides its expression in cancer cells, LINC01116 is markedly expressed in lymphatic endothelial cells of the tumor stroma in which it participates in hypoxia‐sensing and cell proliferation control in lymphangiogenesis.

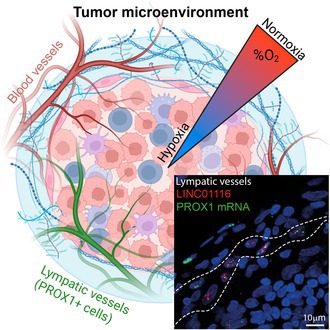

**Abbreviations**
CDH1: Cadherin 1 (E‐cadherin)
CDH5: Cadherin 5 (VE‐cadherin)
CRISPRi: Clustered Regularly Interspaced Short Palindromic Repeats interference, RNA interference
DAPI: 4',6‐diamidino‐2‐phénylindole
DEG: differentially expressed gene
DMEM: Dulbecco's modified Eagle's medium
GSEA: Gene Set Enrichment Analysis
HIF: hypoxia‐inducible factor
HUVEC: human umbilical vein endothelial cells
LEC: lymphatic endothelial cell
LncRNA: long noncoding RNA
LUAD: lung adenocarcinoma
LUCAT1: LUng carcinoma associated transcript 1
MTT: 3‐ [4,5‐ dimethylthiazol‐2‐yl]‐2,5‐diphenyl tetrazolium bromide
NSCLC: nonsmall cell lung cancer
NUMA1: nuclear mitotic apparatus protein 1
ORF: open reading frame
PBS: phosphate‐buffered saline
PDPN: podoplanin
PROX1: prospero homeobox 1
PYCARD: PYD and CARD domain containing
RIOX1: ribosomal oxygenase 1
RNA‐FISH: RNA‐fluorescence *in situ* hybridization
RT‐qPCR: reverse transcriptase quantitative polymerase chain reaction
scRNAseq: single‐cell RNA sequencing
siRNA: small interfering RNA
TCGA: the cancer genome atlas
UMAP: uniform manifold approximation and projection
UMI: unique molecular identifier
VEGF: vascular endothelial growth factor
VWF: von Willebrand Factor

## 1 Introduction

Lung cancer is the first and second leading cause of cancer mortality worldwide in men and women respectively. Nonsmall‐cell lung cancers (NSCLC) are the most common histological forms of bronchopulmonary cancers (around 85% of cases), lung adenocarcinoma (LUAD) being the most frequent subtype. The complex molecular mechanisms governing the initiation, progression, and resistance of NSCLC have hampered the development of effective therapies, necessitating the identification of new biomarkers for early diagnosis, prognosis, and treatment. Although early detection of NSCLC and surgery have improved patient survival, the overall survival rate remains low [1].

The list of molecular drivers for these cancers increases regularly, opening avenues for new potential therapeutic targets [2]. Another important pathophysiological condition driving LUAD is hypoxia. The low oxygenation of tumor cores activates the hypoxia‐inducible factors (HIFs) [3]. These transcription factors regulate many genes involved in a multitude of cellular processes, leading to the initiation of neovascularization, altered glucose metabolism, inhibition of cell death, increased resistance to anticancer agents, tumor cell propagation, and modulation of the immune response [4, 5]. As expected from these pleiotropic effects, the HIF1A gene and its downstream targets have been proposed to classify LUAD and to predict patient survival [6, 7].

Long noncoding RNAs (lncRNAs) represent a class of usually low abundant transcripts, longer than 200 nucleotides. Although their coding potential is debated, with reports demonstrating the functional expression of small peptides generated from small ORFs (open reading frames) found in lncRNAs [8], they are generally described as devoid of protein‐coding capacities. To date, around ten thousand of lncRNA have been identified and validated. These RNAs are often associated with proteins and these ribonucleoprotein complexes exert a wide range of functions, that include the regulation of gene expression, chromatin epigenetic modifications, control of mRNA processing, translation, and stability [9, 10].

In a previous study [11], we have combined transcriptomic analysis of early stage LUAD biopsies from a local patient cohort, as well as A549 LUAD cell line cultured in normoxic and hypoxic conditions to identify a subset of lncRNAs that are both correlated with the hypoxic status of tumors and regulated by hypoxia *in vitro*. Based on this screening, we identified a nuclear form of the lncRNA LUCAT1 (NLUCAT1), validated its high expression in LUAD with a strong hypoxic signature using the Cancer Genome Atlas (TCGA) dataset and documented its contribution to the aggressive LUAD phenotype via oxidative stress regulation.

In the present article, we re‐examined our metadata analyses, including clinical data from LUAD patients, to select candidate lncRNAs that were both hypoxia‐regulated *in vivo* and *in vitro*, but also correlated with poor prognosis when considering overall survival and recurrence data. This identified LINC01116 as the best lncRNA candidate for further research.

LINC01116 has already been associated with a bad prognosis in various cancers (glioblastoma, breast, gastric, prostate) including lung tumors [12]. In the context of LUAD, its silencing has been associated with repression of tumor development through an inhibition of the AKT signaling pathway [13].

LUADs are very heterogeneous tissues constituted of course of epithelial cancer cells but also of a variety of nontumoral cells composing the tumor stroma which include notably cells from immune/inflammatory, mesenchymal, vascular (endothelial), or nervous origins. The vast majority of studies on lncRNA deregulation in tumors are based on large‐scale global expression data from heterogeneous tumor biopsies. Most of them ignore the cellular origins of these deregulations and focus their loss‐ and gain‐of‐function studies exclusively on cancer cells. Single‐cell RNA sequencing methods can now be used to analyze cancer‐related gene expression deregulations, while taking into account the cellular heterogeneity that makes up a tumor.

Despite some consensus on the association of LINC01116 with poor prognosis in certain cancers, including NSCLC, its specificity of expression and role implicating it in a more aggressive phenotype have yet to be established. Here, we embarked on a study to re‐evaluate its function and relevance as a biomarker, focusing notably on the vascular stromal counterpart of LUAD.

## 2 Material and methods

### 2.1 LUAD patients' cohort analyses

As previously mentioned, we used gene expression microarrays data from a local (Nice University Hospital) cohort of 11 peritumoral healthy lungs and 57 early stages LUAD tumors and bulk RNA‐seq data from the TCGA public dataset of 531 LUAD tumors and 59 Healthy lung samples [11]. RNA‐seq data were normalized using the Bioconductor package DESeq2. The hypoxic status of LUAD biopsies was determined a posteriori based on a selection of 27 genes extracted from a hypoxia‐derived metagene signature [14] using a score corresponding to the number of genes with a fold change > 2 compared with the mean average expression in healthy tissues (from 1 to 27: 1–14: ‘Low’; 15–27: ‘High’ hypoxic). Statistical analyses were performed through ANOVA one‐way test with Bonferroni correction for *n* > 2 and *t*‐test for *n* = 2 groups. Survival and recurrence curves were generated using the Kaplan–Meier method, and significance was assessed using the log‐rank test. Stroma or immune score were determined for each patient sample using the cumulative *z*‐score (*z*‐score = (Gene expression—base mean of gene)/standard deviation) of 141 genes based on published signatures [15]. Correlations between LINC01116 and stroma/immune scores or PROX1 were determined by Pearson test.

### 2.2 Cell culture and siRNA transfection

BEAS‐2B (CVCL_0168) human bronchial immortalized cell line and LUAD cell lines (A549 (CVCL_0023), H1975 (CVCL_1511), H2228 (CVCL_1543)) were originally obtained from ATCC (Rockville, MD) and were grown in DMEM supplemented with 10% heat‐inactivated fetal bovine serum and 1% of penicillin/streptomycin. LUAD cell lines have been authenticated using Eurofins Genomics cell line authentication services (Ebersberg, Germany). Primary human LECs (HMVEC‐dLy, human dermal lymphatic microvascular endothelial cells) and HUVEC (Human Umbilical Vein Endothelial Cells) were obtained from Lonza (Basel, Switzerland). Endothelial cells were grown in EBM‐2 Endothelial Cell Growth Medium containing SingleQuots kit EGM™‐2. All cells were incubated at 37 °C in a hypoxia workstation (Invivo2, Baker Ruskinn) set in normoxic condition at 20% O_2_, 5% CO_2_, or in hypoxic condition at 1%O_2_, 5% CO_2_. All experiments were performed on mycoplasma‐free cells.

Two distinct ON‐Targetplus siRNAs (Dharmacon, Lafayette, CO) were purchased for the LINC01116 gene (#1: GGTAACATCAGAATGGCAA; #2: AAATCTGCCTGTTCGAAAA) and one negative control siRNA (siNC#1: UGGUUUACAUGUCGACUAA). Cells were plated and transfected at 50–70% confluency, with individual siRNAs (10 nm) using RNAiMax® (Invitrogen, Thermo Fisher, Illkirch‐Graffenstaden, France) for LUAD cells or INTERFERin® reagent according to the manufacturer's procedure (Polyplus, Illkirch, France) for endothelial cells.

### 2.3 LINC01116 CRISPRi

CRISPRi was performed as previously described [16]. Briefly, the A549 cell line was transduced with a lentiviral vector construction containing two independent expression cassettes: one for dCas9‐KRAB‐MeCP2 (Addgene, Cambridge), a transcriptional repressor fusion protein, and another one conferring resistance to blasticidin (10 μg·mL^−1^). Bulk blasticidin‐resistant cells were cloned and screened to select the most effective clone for CRISPRi‐mediated lncRNA KD [16]. Then A549 dCas9‐KRAB‐MeCP2+ cells were transduced with different lentiviral CROPseq‐Guide‐Puro constructions that drive the expression of either a single guide RNA (sgRNA) against the promoter of LINC01116 (GGAGCCGCGACGTCCAAACC) or a Negative Control sgRNA (GCGCCAAACGTGCCCTGACGG). The transduced cells were selected for resistance to puromycin (1 μg·mL^−1^) (Sigma, St Quentin Fallavier, France). Lentiviral particles were produced by the viral platform facilities of Montpellier (PVM, Biocampus, CNRS UMS3426, France).

### 2.4 LINC01116 cloning and lentiviral transduction

LINC01116 transcript was amplified by RT‐PCR from LEC total RNAs using specific primers (forward:GACCGGTCTCCGCCTGGAAAAGAA; reverse: ATACCATAAGAATGAATTCTATTCTTC). The amplicon (1121 bp) was digested with Age1 and EcoR1 and ligated in the pLJM1 vector (Addgene, Cambridge). Then, transduced A549 and H1975 cells were selected for resistance to puromycin (1 μg·mL^−1^).

### 2.5 RT‐qPCR

Total RNAs were isolated with TRIzol® reagent (Thermo Fisher Scientific) or RNAeasy kit (Qiagen,Les Ulis, France ) according to the manufacturer's instructions. RNAs (1 μg) were retro‐transcribed with the High‐Capacity cDNA Reverse Transcription Kit (Thermo Fisher Scientific), and qPCR was performed on 10 ng of cDNA with SYBR® Green I Master mix (Roche). Gene expression levels were normalized to *RPLP0* level. All reactions were done in triplicate on a LightCycler® 480 Sequence detection system (Roche, Meylan, France), and expression levels were calculated with the comparative CT method ($2^{-\Delta \Delta C_{T}}$). Primers used in this study correspond to LINC01116 forward (5′‐ACCTTTTCCAGACAAGTCAGC‐3′), LINC01116 reverse (5′‐CCACAGGCCCCAAAATGAAT‐3′), RPLP0 forward (5′‐GCATCAGTACCCCATTCTATCAT‐3′), RPLP0 reverse (5′‐AGGTGTAATCCGTCTCCACAGA‐3′), CA9 forward (5′‐CTTGGAAGAAATCGCTGAGG‐3′), CA9 reverse (5′‐ATTGGAAGTAGCGGCTGAAG‐3′), NEAT1 forward (5′‐ACAGTGGCAGGGTTCAATTC‐3′), NEAT1 reverse (5′‐TCAGATGGGGAAATGGAGAG‐3′), SLC2A1 forward (GTGGCCATCTTTTCTGTTGG), and SLC2A1 reverse (GCAGGTTCATCATCAGCATTG).

### 2.6 Single‐molecule RNA fluorescence *in situ* hybridization (RNA‐FISH)

LncRNA‐FISH was performed using RNAscope Multiplex Fluorescent Assays V2 (Advanced Cell Diagnostics, Hayward, CA, USA) according to the manufacturer's protocol. LINC01116‐C1, NEAT1‐C2, and PROX1‐C3 commercial probes were used to detect these RNAs. Briefly, cells cultured on Lab‐Tek chamber slides were fixed in 3.7% formalin in PBS for 15 min at room temperature, then washed in PBS and digested with 1/10 diluted protease III for 15 min at RT. Cells were hybridized with probes for 2 h at 40 °C in hybridization buffer, then washed twice for 2 min. For tissue cryosections, sections were dehydrated, HRP blocked and digested with protease III for 30 min at 40 °C before probe hybridization. The signal was revealed by sequential incubations with amplifier reagents and dyes according to the manufacturer's protocol. Co‐immunolabeling of VWF (anti‐rabbit, Abcam, ab6994) or E‐Cadherin (anti‐rabbit, cell signaling, 24E10) was performed following RNA‐FISH experiments. Briefly, sections were blocked for 1 h in Animal‐Free blocking solution (Cell Signaling) and incubated overnight at 4 °C with primary antibodies. After washing with PBS, anti‐rabbit Alexa Fluor 488 (A‐11055; Thermo Fisher Scientific) was added for 1 h at room temperature. DAPI was used to counterstain nuclei and coverslips were mounted onto glass slides using Fluoromount‐G mounting medium (Thermo Fisher). Fluorescence was visualized and captured on a Zeiss LSM780 confocal microscope.

### 2.7 Subcellular fractionation

Cytosolic and nuclear fractions were prepared as previously described [11], and cytosolic and nuclear RNAs were extracted with TRIzol® and TRIzol®LS reagents (Thermo Fisher Scientific), respectively.

### 2.8 Cell migration assays

Boyden's chamber migration assays were performed as previously described [17]. Cells were detached, resuspended in serum‐free medium, seeded in the upper chambers of transwell inserts (50 000 cells/insert), and attracted in the lower chambers by medium supplemented with 5% of FCS. After 24 h at 37 °C, the nonmigrating cells on the top of the membrane were removed with a cotton swab and cells on the lower side of the filters were fixed, permeabilized, stained with DAPI, and scored in at least 5 independent microscope fields.

### 2.9 Cell proliferation assay

Cell proliferation was monitored by cell counting, MTT and CYQUANT assays. For counting assays, cells were detached and seeded at 2 × 10^5^ cells per 60 mm petri dish in triplicate for each timepoint and counted in duplicate 48, 72, and 96 h later after detachment using a Malassez hemocytometer. MTT or CYQUANT assays (Sigma‐Aldrich) were done according to the manufacturer protocols in 96 wells seeded at 5000 cells per well.

### 2.10 Whole genome Agilent microarray analyses

Transcriptome analyses of A549 xenografts were performed on SurePrint G3 Human Gene Expression v2 8 × 60K microarrays from Agilent Technology (Loveland, CO, USA). RNA samples were labeled with Cy3 dye using the low RNA input QuickAmp kit (Agilent), and 825 ng of labeled cRNA probes were hybridized on microarrays as recommended by the supplier. Data analyses were performed using R software. The quality control was performed using the Bioconductor package arrayQualityMetrics and custom R scripts. Additional analyses were performed using the Bioconductor package Limma. Briefly, data were normalized using the quantile method. Replicated probes were averaged after normalization and control probes removed. We used a linear modeling approach to calculate log ratios, moderated *t* statistics, log odds ratios of differential expression (B statistic), and *P*‐values for each comparison of interest. *P*‐values were adjusted for multiple testing using the Benjamini and Hochberg method, which controls the false discovery rate.

Previous data from the local cohort (57 LUAD and 11 peritumoral normal lung tissues) and A549 cultivated in normoxic and hypoxic conditions were reused to highlight the expression of LINC01116 [11]. Spearman's rank correlation was applied, and probes were ranked according to *P*‐values. Selection of hypoxia‐associated lncRNAs candidate genes was performed using thresholds of 0.05 for the false discovery rate and 1.6‐fold for the gene expression ratio between tumors and the healthy lung controls, and an adjusted *P*‐value for LUAD hypoxic status pca < 0.05. Additional comparisons between high versus low hypoxic groups were performed in order to confirm our hypothesis.

For A549 xenografts repressed (sgLINC01116) or not (sgNC) for LINC01116, 4 distinct xenografts were compared (Data set 1). Differentially expressed genes were selected based on a log_2_(intensity) above 6, an absolute log_2_(fold change) above 0.5 and an adj. *P*‐value below 0.05. Enrichment in biological processes and biological networks analysis were performed using Ingenuity Pathway Analysis software (Qiagen) (http://www.ingenuity.com/).

### 2.11 RNA sequencing

For siRNA‐transfected LECs, libraries preparation was performed at the GenomEast platform at the Institute of Genetics and Molecular and Cellular Biology (IGBMC, Illkirch, France). Libraries were generated from 200 ng of total RNA using Lexogen 3′ mRNA‐Seq Library Prep Kit (015, QuantSeq 3′ mRNA‐Seq V2 Library Prep Kit FWD with Unique Dual Indices (12 nt)) (Lexogen, Vienna, Austria) according to manufacturer's instructions. Library generation was initiated by oligo(dT) priming. After first strand synthesis, the RNA was removed and second strand synthesis was initiated using a random primer containing an UMI (081, UMI Second Strand Synthesis Module for QuantSeq FWD) (Lexogen). DNA libraries were amplified using 15 cycles of PCR adding Lexogen UDI 12 nt single indexes. Surplus PCR primers were further removed by a first purification using PB beads and a second purification using SPRIselect beads (Beckman Coulter, Villepinte, France). The final libraries were checked for quality and quantified using Bioanalyzer 2100 system (Agilent Technologies, Les Ulis, France). Libraries were sequenced on an Illumina NextSeq 2000 sequencer as single read 100 base reads. Image analysis and base calling were performed using RTA version 2.7.7 and BCL Convert version 3.8.4. Reads were trimmed and aligned with salmon methods (STAR package). Differential expression between si‐CTRL and si‐LINC01116 in normoxia and hypoxia were determined by DEseq2 package.

For A549 in normoxic and hypoxic conditions, libraries were generated from 500 ng of total RNAs using TruSeq Stranded Total RNA Library Prep kit with Ribo‐Zero (Illumina, Paris, France) according to the manufacturer's instructions. Libraries were then quantified with KAPA library quantification kit (Kapa Biosystems) and pooled. 4nmoles of this pool were loaded on a high output flowcell and sequenced on a NextSeq500 sequencer (Illumina) with 2 × 75 bp paired‐end chemistry. Reads were aligned to the human genome release hg38 using STAR v2.4.0a with default parameters.

### 2.12 Animal experiments

All animal care and experimental protocols were conducted according to European, national and institutional regulations (Protocol number APAFIS#24742, IPMC approval E061525). Laboratory staff carried out all experimental protocols under strict guidelines to ensure careful and consistent handling of the mice. The animals were maintained under a 12‐h light–dark cycle with free access to food and water. NMRI‐*Foxn1* ^*nu*^ mice (female 6–8 weeks old) from Charles River® were used throughout this study. For LUAD xenograft assays, NMRI‐*Foxn1* ^*nu*^ mice (*n* = 20) were anesthetized using 2% isoflurane and injected subcutaneously as previously described [18] with A549 cells (10 × 10^6^ cells per mouse in 50% Matrigel (Corning Life science, Amsterdam, The Netherlands)) transduced with sgNC or sgLINC01116. The growth tumor curves were determined by measuring the tumor size with calipers and calculating their volume using the equation V = (LxW^2^)/2. Mice were killed by cervical dislocation.

### 2.13 Single‐cell RNA‐seq dataset processing and cell clustering

The scRNA‐seq datasets were downloaded from GEO for GSE136831 [19], GSE253013 [20], and GSE164829 [21] datasets. Preprocessed data were processed and analyzed using the R package Seurat (v.4.1.0) on RStudio (v.4.1.2). After removing ribosomal genes, genes expressed in fewer than 3 cells and cells expressing fewer than 200 genes, we filtered out cells with fewer than 200 unique feature counts (low‐quality cells). Cells with unique feature counts greater than 7500 genes (more than twice the median number which can correspond to possible doublets) and cells with more than 5 percent of mitochondrial genes (apoptotic or lysed cells) were also removed. We then normalized data using the NormalizeData function and extracted highly variable features using the FindVariableFeatures function. Normalized data underwent a linear transformation for GSE136831/GSE164829 and logarithm transformation for GSE253013 (scaling, ScaleData function) and principal component analysis (PCA) based on variable features using the RunPCA function. Graph‐based clustering was then performed according to gene expression profiles using the FindNeighbors (dims = 1 : 50 for GSE136831; 1 : 30 for GSE253013; 1 : 50 for GSE164829) and FindClusters (resolution = 0.5 for GSE136831; 0.3 for GSE253013, 0.5 for GSE164829) functions, and results were visualized using a nonlinear dimensional reduction Uniform Manifold Approximation and Projection (UMAP) technique running RunUMAP (dims = 1 : 30 for GSE253013; 1 : 50 for GSE164829), dimensional reduction Harmony technique for GSE136831 (1 : 50) and DimPlot functions. Cells were clustered with their published clustering, and lymphatic endothelial cells were highlighted by several gene markers (*PROX1, PDPN, CDH5*, and *VWF*). Gene expressions were visualized using FeaturePlot functions and Violin Plot functions.

### 2.14 Statistical analyses

Graph and statistical analysis were performed with GraphPad (v.9) and R (v.4.1.0). For parametric tests, Student's *t*‐test (*n* = 2 groups) and ANOVA one‐way test with Bonferroni correction (*n* > 2 groups) were used. For nonparametric tests, Mann–Whitney (*n* = 2) and Kruskal‐Wallis test with Holm's multiple comparisons test (*n* > 2 groups) were used. Data were considered significant at a *P*‐value inferior to 0.05. Statistically significant differences are indicated (*P < 0.05, **P < 0.01, ***P < 0.001, ****P < 0.0001).

## 3 Results

### 3.1 Characterization of LINC01116 expression and regulation in LUAD tumor cells

**Fig. 1:**
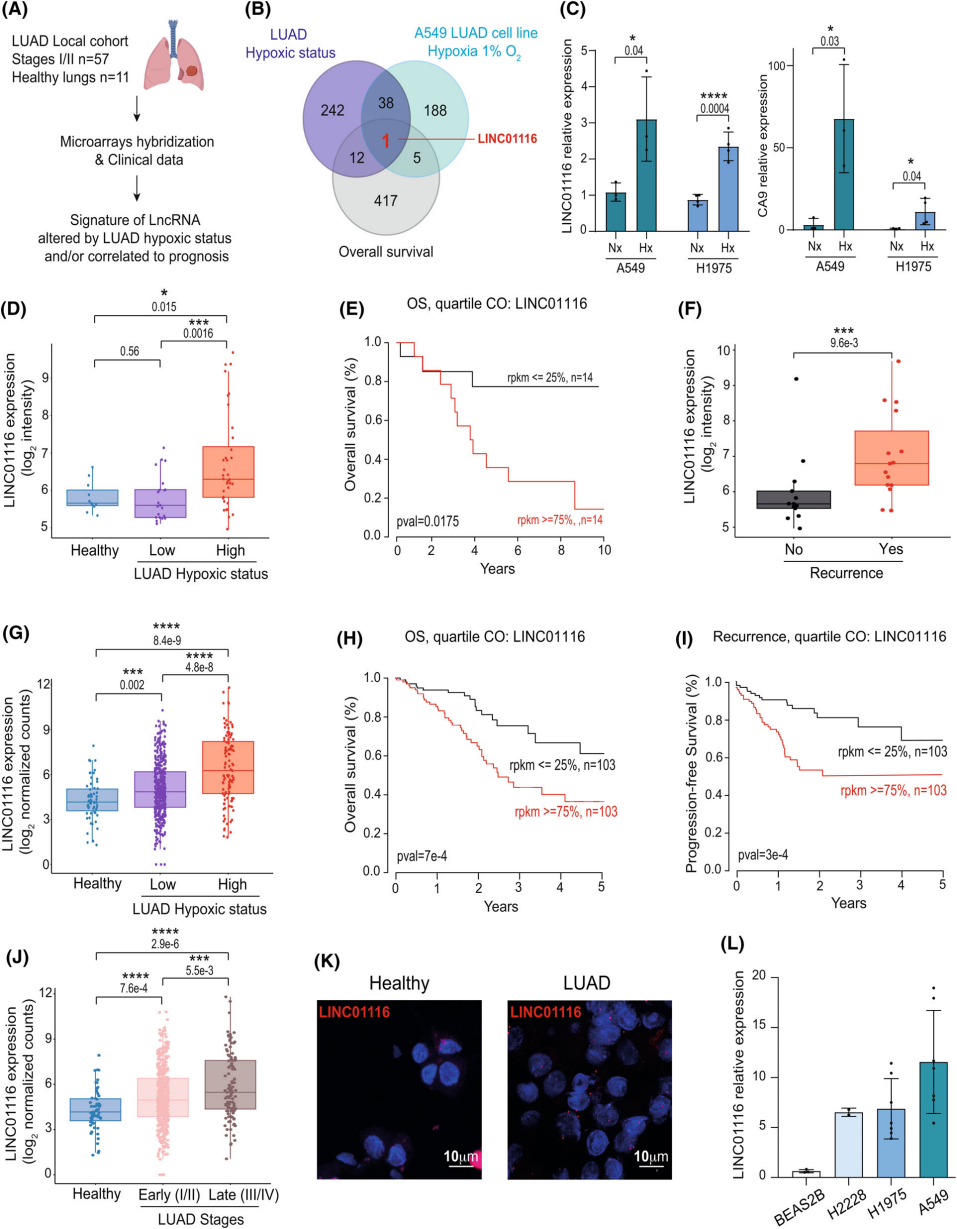
LINC01116 expression is correlated with high hypoxic status and bad prognosis in LUADs. (A,B,D–F) Local Lung Adenocarcinoma (LUAD) cohort: (A) Schematic representation of analyses performed on early stage tumor (*n* = 57) and healthy lung (*n* = 11) samples. (B) Venn diagram showing lncRNA candidates whose expression is correlated to LUAD hypoxic status (log_2_(intensity) > 6, Hx.pca.p.1‐value < 0.001) and/or bad prognosis in the local cohort (OS.chi2.pval < 0.05) and/or modulation in A549 LUAD cells cultured in 1% O_2_ for 24 h (absolute log_2_(hypoxia/normoxia) > 0.5, log_2_(intensity) > 6 and adj. *P*‐value< 0.05). (C) RT‐qPCR analysis of LINC01116 and CA9 (hypoxia‐regulated gene) expression in A549 and H1975 cell lines cultured in normoxic (Nx, 20% O_2_) or hypoxic conditions (Hx, 1% O_2_) for 24 h. Data are means ± SD of at least 3 independent experiments. (D) Boxplot representation of LINC01116 expression in safe lungs (*n* = 11) and in LUAD sample subsets according to their hypoxic status (Low, *n* = 12, High, *n* = 20). The results are displayed on a log_2_ scale. Statistical relevance of the comparisons was determined with the Wilcoxon's test. (E) Kaplan–Meier analyses of correlations between the LINC01116 expression level and overall survival of LUAD patients by quartiles. *P*‐values were calculated using the log‐rank test. (F) Boxplot representation of LINC01116 expression in LUAD sample subsets according to patient recurrence status (No, *n* = 26, Yes, *n* = 31). The results are displayed on a log_2_ scale. Statistical relevance of the comparisons was determined with the Wilcoxon's test. (G–J) TCGA (The Cancer Genome Atlas) LUAD cohort: (G) Distribution of LINC01116 expression in the LUAD samples according to their subset hypoxic status (Healthy, *n* = 59, Low, *n* = 428, High, *n* = 103). (H,I) Kaplan–Meier analyses of correlations between the LINC01116 expression level and overall survival (H) or recurrence (I) of the LUAD patients by quartiles. (J) Boxplots showing the distribution of LINC01116 expression in the TCGA LUAD cohort according to tumor stage (Healthy, *n* = 59, Early (I/II), *n* = 418, Late (III/IV), *n* = 113). (K) RNA‐Fluorescence *In Situ* Hybridization for LINC01116 (in red) in peritumoral (Healthy) and tumor samples (LUAD). Nuclei were counterstained with DAPI (in blue). Scale bar = 10 μm. (L) Analysis of LINC01116 expression by RT‐qPCR in the BEAS2B (*n* = 3), H2228 (*n* = 2), H1975 (*n* = 7), and A549 (*n* = 7) cell lines. Data are means ± SD. Each dot represents a tissue sample or an independent experiment. Statistical analyses were done using: an unpaired Student's *t‐*test for *n* = 2 groups, one‐way ANOVA for *n* > 2 groups with Bonferroni's correction for multiple comparisons. Box plot are medians ± quartiles.

We used clinical data on patients' survival and transcriptomic profiling data that we previously performed using gene expression microarrays on 57 early stage tumors and 11 healthy peritumoral lungs from a local LUAD patient cohort, to identify lncRNAs that were modulated by hypoxia and associated with a poor prognosis (Fig. 1A) [11]. As previously mentioned, tumor samples were *a posteriori* classified according to their hypoxic status using a metagene signature [11]. *In silico* analyses highlighted a signature of 13 lncRNAs dysregulated by hypoxia in tumor biopsies (8 lncRNAs were positively correlated with the hypoxic status of tumors, whereas 5 lncRNAs were negatively correlated with the hypoxic status) and associated with a poor prognosis of patients (Fig. 1B and Table S1). Among these transcripts, only LINC01116 was also induced by hypoxia *in vitro* in the A549 LUAD cell line (Fig. 1B,C) as well as in the H1975 LUAD cell line (Fig. 1C). In‐depth analyses performed on LINC01116 expression confirmed that it is preferentially upregulated in LUAD with a high hypoxic status (Fig. 1D), and that its high expression is associated with a decrease in patients' overall survival and conversely a stronger recurrence rate (Fig. 1E,F).

These data were confirmed using a LUAD public dataset from the TCGA (all tumor stages, *n* = 522) (Fig. 1G–I). In this larger cohort, LINC01116 was found more expressed in late stage (III/IV) LUADs compared to early stages (I/II) (Fig. 1J). In addition, its expression was significantly increased in tumors harboring a low hypoxic status compared to healthy lung samples (Fig. 1G) which was not the case in our small local cohort (Fig. 1D). Accordingly, RNA‐FISH confirmed a stronger expression of LINC01116 in tumors compared to peritumoral tissues (Fig. 1K). A higher expression of LINC01116 was also observed in different LUAD cell lines (A549, H1975, and H2228) than in the human Beas‐2B immortalized normal bronchial epithelial cell line (Fig. 1L).

**Fig. 2:**
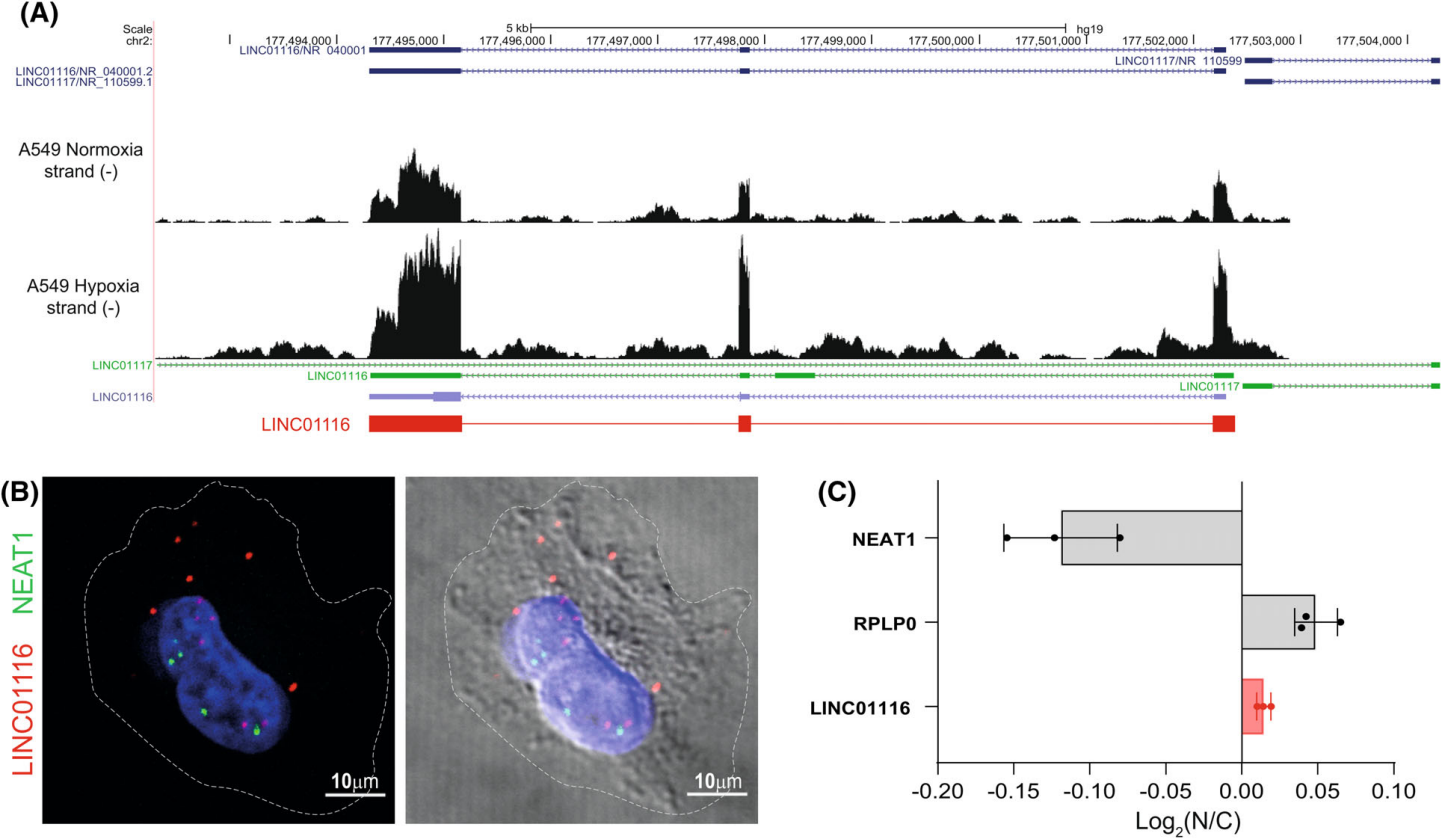
LINC01116 structure and subcellular localization. (A) Screenshot from UCSC genome browser displaying raw RNA sequencing (RNA‐seq) reads (NextSeq500 Illumina 150 bp paired‐end reads) for normoxic (20% O_2_) or hypoxic (1.2% O_2_, 24 h) A549 cells plotted on LINC01116 genomic region. (B) RNA‐Fluorescence *In Situ* Hybridization: A549 cells were fixed, hybridized for LINC01116 (in red) and NEAT1 (in green) probes, and nuclei were counterstained with DAPI (in blue). Cells were visualized by confocal microscopy. The right panel shows brightfield imaging to visualize the cell membrane. Scale bar = 10 μm. C. RT‐qPCR analysis of LINC01116 expression in nuclear and cytosolic subcellular fractions of A549 cells. Horizontal bars represent the ratio of the expression of the indicated RNAs between the nucleus and the cytosol. NEAT1 is a specific marker of the nuclear fraction, whereas RPLP0 is mainly cytoplasmic. Data are means ± SD of 3 independent experiments.

Short and long‐reads RNA sequencing data were used to confirm that LINC01116 is a divergent lncRNA with a sequence of 1121 nucleotides composed of 3 exons (Fig. 2A). In agreement with previously published data, RNA‐FISH and subcellular fractionation experiments demonstrated that LINC01116 is predominantly a cytosolic transcript (Fig. 2B,C) [13, 22].

### 3.2 Evaluation of LINC01116 biological function in LUAD tumor cells

**Fig. 3:**
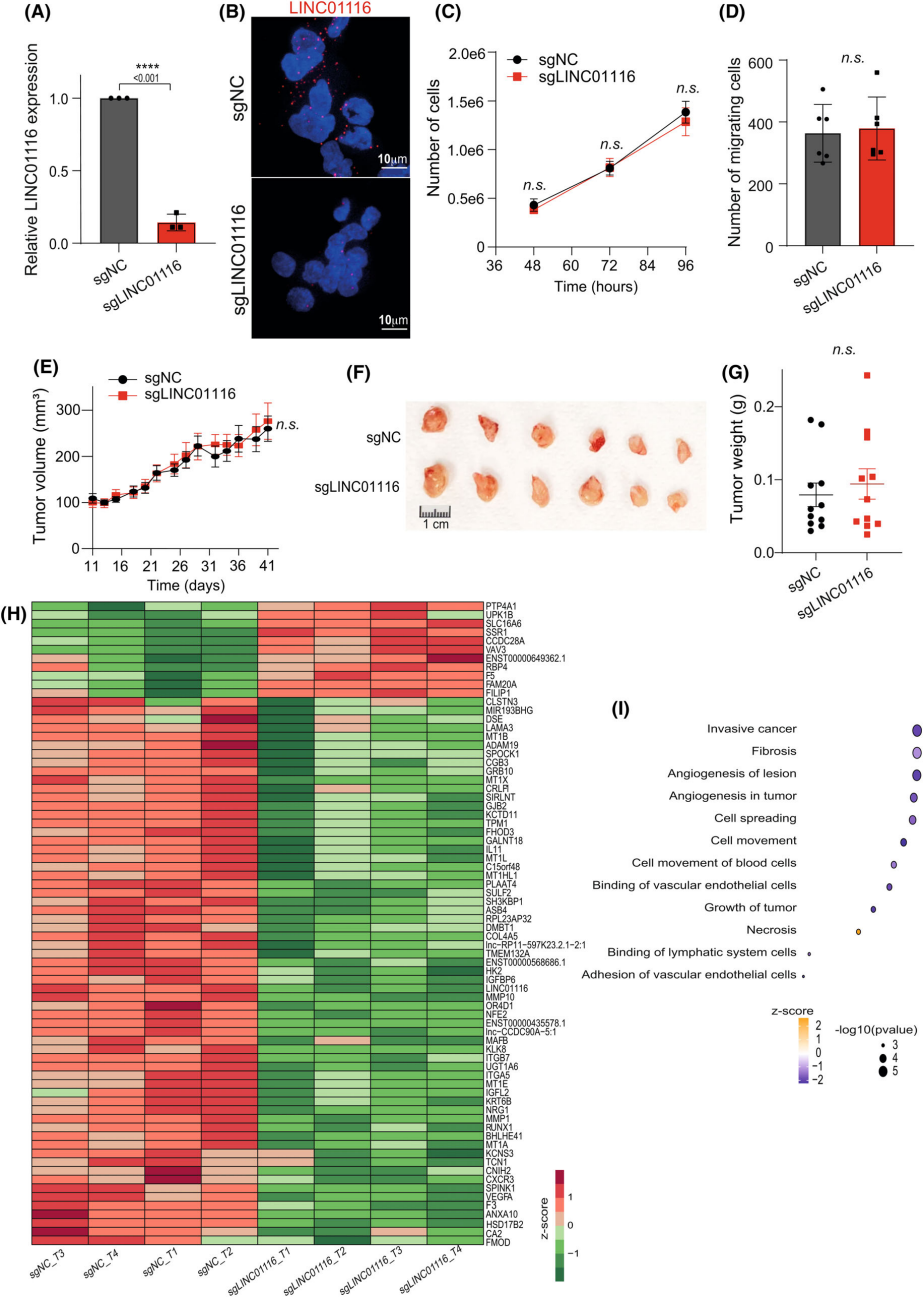
Impact of CRISPRi‐mediated LINC01116‐knockdown on LUAD tumor cells.(A,B) LINC01116 knockdown was analyzed by RT‐qPCR (A) or RNA‐Fluorescence *In Situ* Hybridization (B) in A549 cells transduced with single guide RNA (sgRNA), sgNC or sgLINC01116. Nuclei were counterstained with DAPI (blue). Scale bar = 10 μm. (C) A549 cells stably expressing the indicated sgRNA were plated (250 000/well) in triplicate and counted at the indicated times. (D) Comparative analysis of migration of transduced cells (50 000), 24 h after seeding in the upper chambers of Transwell inserts and attraction in the lower chambers. Each dot represents an insert. (A,C,D) Data are means ± SD of three independent experiments. (E–G) Tumor engraftment experiments: (E) A549 cells (10^7^ per mouse in 50% Matrigel) transduced with sgNC or sgLINC01116 were injected subcutaneously on the flank of Nude mice, and tumor size was measured at the indicated times. Data are means ± SEM of 11 tumors in each condition. (F,G) Pictures and weight of xenografts at 46 days after engraftment. (H) Unsupervised hierarchical clustering of DEGs between sgNC‐ and sgLINC01116‐transduced A549 cells tumor xenografts (*n* = 4) at 46 days after engraftment. The heat map shows the 74 genes for which expression is statistically modulated by LINC01116 KD based on a log_2_(intensity) > 6, an absolute log_2_(fold change) > 0.5 and an adj. *P*‐value < 0.05. Expression corresponds to normalized log_2_ intensity followed by median centering. Clustering was performed using an Euclidian distance metric and average linkage. (I) Function enrichment analysis on differentially expressed genes (DEGs) was done using IPA™. For all graphs of the Fig. 3, statistical analyses: Student's *t*‐test for *n* = 2 groups, and two‐way ANOVA for *n* > 2 groups.

To decipher the role of LINC01116 in LUADs, we developed loss‐ and gain‐of‐function approaches to characterize the consequences of the modulation of its expression in the A549 LUAD cell line. First, we used a CRISPRi approach that efficiently repressed by 90% the expression of LINC01116 in the A549 cell line, as shown both by qPCR and RNA‐FISH analyses (Fig. 3A,B). We found that LINC01116 knockdown failed to affect cell proliferation and cell migration *in vitro* (Fig. 3C,D). Similar results were obtained when knockdown of LINC01116 was done using RNA interference. Indeed, transient transfection of A549 or H1975 LUAD cells with siRNAs markedly inhibited the expression of the transcript (Fig. S1A,D,G) but had no effect on their proliferative and migrating properties (Fig. S1B,C,E,F).

Accordingly, the ectopic overexpression of LINC01116 in these cell lines (Fig. S1H,K,N) did not alter these cellular processes (Fig. S1I,J,L,M).

We next tested whether LINC01116 could affect the three‐dimensional growth capacity of LUAD cells. When A549 cells, stably repressed or not for LINC01116 expression by CRISPRi, were injected subcutaneously in nude mice to generate tumor xenografts, we observed similar growth curves (Fig. 3E). Assessment of tumor size after excision confirmed that LINC01116 knockdown had no effect on tumor growth (Fig. 3F,G). In order to evaluate the potential regulatory role of LINC01116 in A549 cells, we performed a bulk RNA‐seq on tumor xenograft samples at 46 days after engraftment (*n* = 4). The comparative transcriptomic analysis of LUAD cell line xenografts revealed a slight significant alteration of the expression of 74 genes, that were mainly downregulated (*n* = 63) in tumors in which the expression of LINC01116 was repressed by 90% (sgLINC01116) compared to control tumors (sgNC) (Fig. 3H, Fig. S2). Their annotation using Ingenuity Pathway Analysis™ indicated a significant enrichment of Gene Ontology Disease or functions terms such as invasive cancer, fibrosis, tumor angiogenesis, and cell movement (Fig. 3I), suggesting a potential alteration of pathways related to interaction with stromal components. It should be noted, that none of the deregulated genes were located in the region adjacent to the LINC01116 locus, ruling out local cis‐regulation as an explanation for these expression modulations. Altogether, loss‐ and gain‐of‐function studies of LINC01116 in LUAD cell lines mainly indicated mild repression of a short subset of genes related to stromal communication, with no significant effect on tumor cell properties, both *in vitro* and in the context of a xenograft model.

### 3.3 Characterization of LINC01116 expression in LUAD vascular stromal components

Based on these data, we further investigated the precise cellular source of LINC01116 in LUAD using several approaches. Interestingly, we first observed in LUAD samples that the LINC01116 RNA‐FISH probe signal was expressed in cells that were positive for E‐cadherin (CDH1) immunofluorescence signal, but also in cells expressing the endothelial cell marker von Willebrand Factor (VWF) (Fig. S3), indicating that in addition to epithelial cancer cells, LINC01116 is also expressed in some vascular vessels' components of the tumor stroma and notably in vessels devoid of red blood cells.

**Fig. 4:**
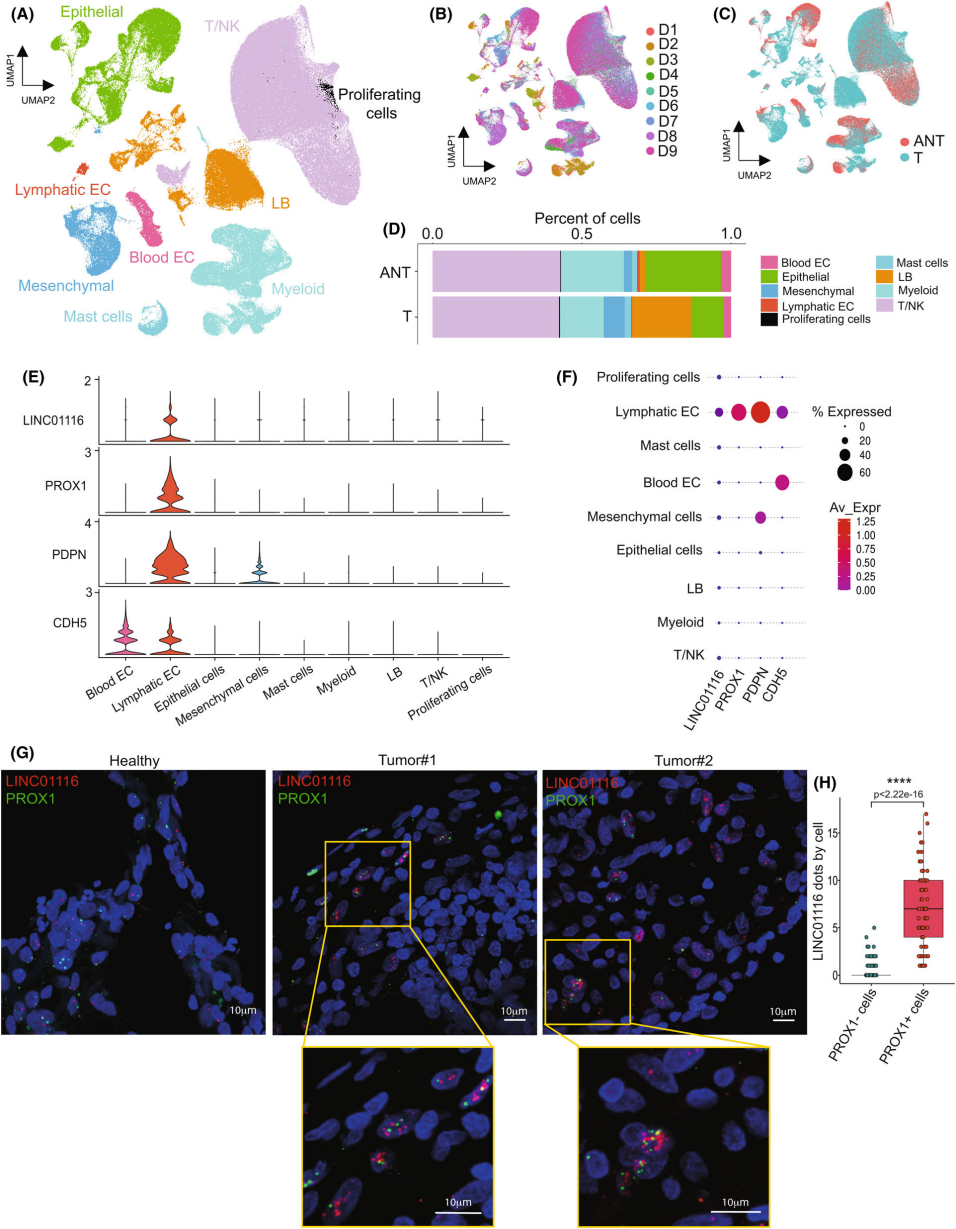
LINC01116 is expressed in lymphatic endothelial cells in the stromal vasculature of LUAD. (A) Uniform Manifold Approximation and Projection (UMAP) plots of single‐cell RNA sequencing (scRNA‐seq) expression data from LUAD tumor (T) and adjacent nontumor (ANT) stromal cells from the GSE253013 public dataset clustered into Blood EC, lymphatic EC, mast cells, B lymphocytes (LB), T/NK, epithelial, mesenchymal, myeloid and proliferating cells. (B, C) UMAP plots of the GSE253013 dataset colored by donors (B, D1‐D9) or tissue types (C, ANT, T). D. Bar plots of subclusters repartition in function of tissue type (ANT or T). E. Violin plots showing the expression of LINC01116, PROX1, PDPN, and CDH5 in each cluster. F. Dot plots showing the expression and repartition of LINC01116, PROX1, PDPN, and CDH5 in each cluster. G. RNA‐Fluorescence *In Situ* Hybridization for LINC01116 (in red) and PROX1 (in green) in peritumoral lung tissue and in LUAD tumor samples from the local cohort. DAPI counterstaining of nuclei in blue. Scale bar = 10 μm. H. Boxplot representation of LINC01116 dots quantification by PROX1^−^ (*n* = 229) and PROX1^+^ (*n* = 65) cells. Box plot are medians ± quartiles. Statistical analysis: Mann–Whitney test.

We then analyzed a publicly available single‐cell RNA sequencing (scRNA‐seq) dataset describing the stroma of both LUAD and adjacent nontumoral tissues (from Xiang *et al*. [20]). Results showed that LINC01116 was strictly limited to podoplanin (PDPN)^+^, VE‐cadherin (CDH5)^+^, and prospero homeobox 1 (PROX1)^+^ cells, which are classic markers of lymphatic endothelial cells (LEC), while it was not detected in other types of stromal cells, notably blood endothelial cells, as well as mesenchymal or immune cells (Fig. 4A–F). This result was corroborated by RNA‐FISH on LUAD biopsies showing a colabeling of the LINC01116 probe in PROX1^+^ cells both in peritumoral and in tumoral tissues, while the LINC01116‐specific signal was hardly detectable in PROX1^−^ cells (Fig. 4G,H). Moreover, by analyzing global gene expression profiling data from our local and TCGA LUAD cohorts, we found a positive correlation between the expression level of LINC01116 and that of PROX1 in both cohorts (Fig. S4A,B). We also evaluated the association between LINC01116 expression and stromal and immune scores of LUAD samples, defined by specific published gene signatures [15]. We observed no correlation with the stromal/mesenchymal signature, which does not contain any specific lymphatic endothelial cell markers (Fig. S4C,D). Conversely, we found that higher expression of LINC01116 in tumors was associated with a significant decrease in immune cell infiltration (Fig. S4E,F).

**Fig. 5:**
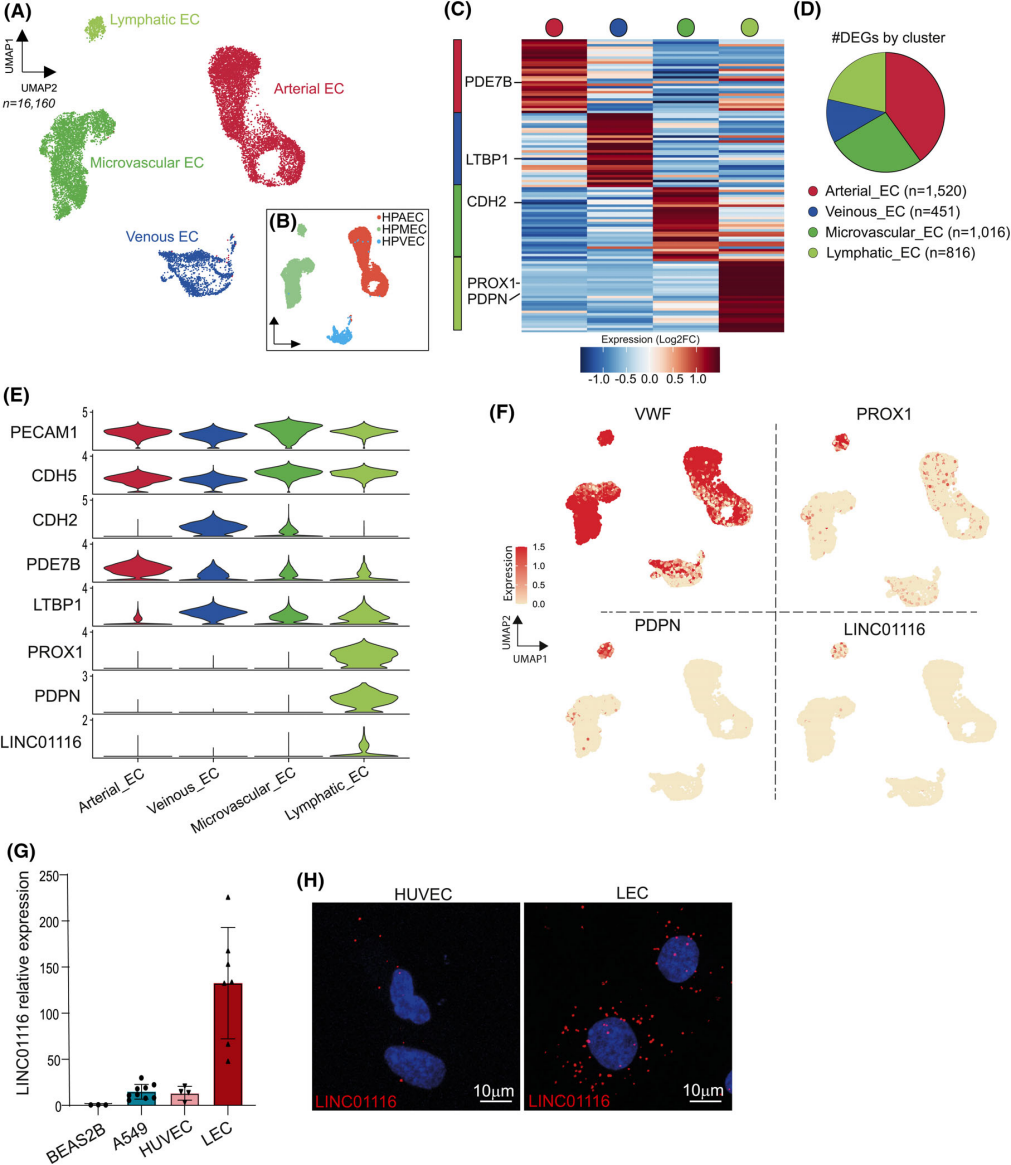
LINC01116 is expressed in primary lymphatic endothelial cells. (A) UMAP plots of scRNA‐seq expression data from 16 160 primary pulmonary endothelial cells from GSE164829 public dataset containing human pulmonary arterial endothelial cells (HPAEC), human pulmonary microvascular endothelial cells (HPMEC), human pulmonary veinous endothelial cells (HPVEC) from Lonza clusterized into Arterial, Veinous, Microvascular and Lymphatic EC. (B) UMAP plots of cells from GSE164829 dataset colored by sample source. (C) Unsupervised hierarchical clustering of DEGs between the four endothelial cell subtypes. The heatmap shows the 30 top‐ranking marker genes specific of each cluster. Key genes are indicated on the left. (D) Number of DEGs in each cluster. (E) Violin plots showing the expression of LINC01116 and key marker genes in each pulmonary endothelial cell subtypes. (F) UMAP plots of VWF, PROX1, PDPN, and LINC01116 expression in each endothelial cell subtypes. (G) Analysis of LINC01116 expression by RT‐qPCR in BEAS2B (*n* = 3) and A549 (*n* = 9) cell lines, and in primary HUVEC (blood large vessel endothelial cell, *n* = 4) and LEC (Lymphatic endothelial cell, *n* = 7) cultures. Data are means ± SD. Statistical analyses: Student's *t*‐test unpaired. (H) RNA‐Fluorescence *In Situ* Hybridization of LINC01116 in HUVEC and LEC primary cultures. Nuclei were counterstained with DAPI (in blue). Scale bar = 10 μm.

To emphasize the selectivity of expression of LINC01116 in LECs, we also revisited two other public scRNA‐seq datasets that respectively document (i) the composition of normal lung tissue [19] (Fig. S5), and (ii) the in‐depth molecular description of the different endothelial cell types present in human lung tissue using primary cultures of endothelial cells of various origins, namely arterial, venous, microvascular, and lymphatic (GSE164829, Fig. 5A–F). These analyses, together with qPCR and RNA‐FISH experiments performed on our primary cultures of HUVEC (human umbilical vein endothelial cells) and LEC, Beas‐2B (normal bronchial epithelial) and A549 (LUAD) cell lines (Fig. 5G,H), confirmed that LINC01116 expression is at least 10 times more expressed in LECs compared to other cell types.

We therefore hypothesized that LINC01116 could be a marker of lymphangiogenesis that could be used to highlight tumor lymphangiogenesis. These results suggest that, although expressed in tumor cells, LINC01116 is largely enriched in lymphatic endothelial cells, and that the latter are the main source of its expression in healthy and LUAD‐bearing human lungs.

### 3.4 Impact of LINC01116 knockdown in LEC

**Fig. 6:**
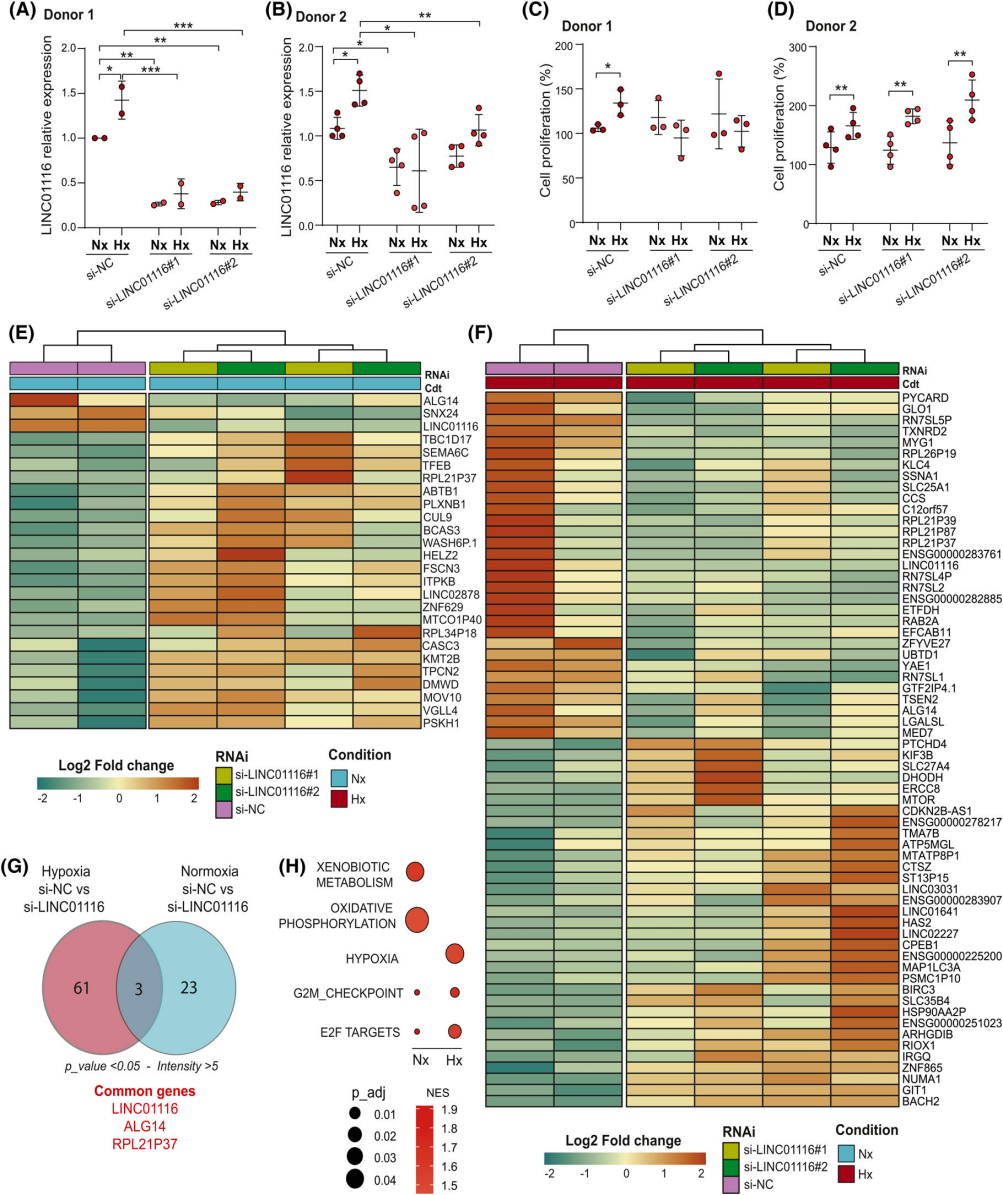
Impact of RNAi‐mediated LINC01116 knockdown in lymphatic endothelial cells. (A–H) Primary LECs from two donors were transfected with si‐LINC01116#1, si‐LINC01116#2 or control si‐NC and then cultured in normoxic (Nx) or hypoxic (Hx) conditions for 48 h (A–D) or 24 h (E–H), respectively. (A, C) Knockdown of LINC01116 was monitored through RT‐qPCR. Data are means ± SD of 2 and 4 independent experiments respectively. (B, D) Cyquant proliferation assay was done after 48 h of culture in normoxic or hypoxic conditions. Data are means ± SD of 3 and 4 independent experiments, respectively. (E, F) Heatmaps of DEGs expressed in *z*‐score in LECs silenced or not for LINC01116 and cultured in normoxic (F) or hypoxic (G) conditions. (G) Venn diagram showing DEGs (absolute log_2_(fold change) > 0.5, intensity > 5 and *P*_value < 0.05) in LEC silenced or not for LINC01116 and cultured in normoxic or hypoxic conditions. (H) Functional annotations of RNA sequencing data using IPA software. Statistical analyses: ANOVA two‐way with Bonferroni correction for (A) and Multiple student's test for (B–D).

Since hypoxia promotes lymphangiogenesis in solid tumors [23], we used an RNA interference approach to elucidate the role of LINC01116 in LEC primary cultures from two donors in normoxic or hypoxic conditions. First, we observed an induction of LINC01116 expression by hypoxia in LECs (Fig. 6A,B), albeit of a lesser magnitude than that measured in LUAD cell lines (Fig. 1C). The two siRNAs targeting LINC01116 efficiently repressed by 75–80% the expression of the transcript in LECs from donor#1 (Fig. 6A), while this interference was much less efficient in cells from donor #2 (20–30%) (Fig. 6B). In these conditions, the inhibition of LINC01116 using siRNAs clearly impacted the induction of the proliferation of LECs from donor #1 under hypoxia (Fig. 6C), which was not the case in LECs from donor#2 (Fig. 6D).

To go further, we decided to characterize the effects of LINC01116 KD on the transcriptomic signature of LECs from donor #1 cultured in normoxia or hypoxia through RNA‐seq analyses (Fig. 6E–H). In normoxic conditions, 26 genes were dysregulated in si‐LINC01116‐treated cells compared to si‐NC conditions including LINC01116 (Fig. 6E,G). Under hypoxic stimulation, the expression of 64 differentially expressed genes (DEGs) was altered by LINC01116 KD (Fig. 6F,G). Among the set of deregulated genes, some are involved in cell division like NUMA1 (nuclear mitotic apparatus protein 1), or sensing of hypoxia like RIOX1 (ribosomal oxygenase 1). Interestingly, these genes were repressed under hypoxia in siNC‐expressing LECs while their expression was maintained or upregulated, respectively, in cells repressed for LINC01116 expression (Fig. S6A,B). Conversely, the induction of PYCARD in response to hypoxia was abolished by LINC01116 KD (Fig. S6E). Other genes, including YAE1 and MYG1, were repressed in hypoxia in LECs treated with LINC01116 siRNAs, while not in control (siNC) cells (Fig. S6C,D). Finally, Gene Set Enrichment Analysis (GSEA) of this gene signature highlighted molecular hallmarks of pathways like hypoxia, oxidative phosphorylation, and regulation of cell cycle progression with the terms G2M checkpoint and E2F targets (Fig. 6H).

These results are in line with the antiproliferative effect observed under hypoxia and also suggest a role for LINC01116 in hypoxia sensing in LECs.

## 4 Discussion

In the present work, we demonstrate that LINC01116 is a cytosolic long noncoding RNA whose expression is induced by hypoxia and associated with a poor prognosis and high recurrence in LUAD patients. Growing evidence supports the dysregulation of numerous lncRNAs in response to hypoxic stress and their hypoxia‐regulated contribution to tumor hallmarks [24]. In this context, LINC01116 has previously been reported to be upregulated by hypoxia in esophageal cancer cell lines *in vitro* [25]. Our data clearly bring evidence for the first time, that hypoxia is one of the main environment factors driving the increased expression of this transcript in LUAD tumors. Furthermore, we clarify the cellular origin of this dysregulation, showing that LINC01116 is indeed expressed in tumor epithelial cancer cells but also at a higher level in LECs within the tumor stroma.

Concerning the functional role of LINC01116 in NSCLC, previous reports suggested that this transcript had a direct pro‐tumoral role in LUAD cancer cells, showing alteration of cellular processes involved in cancer development such as cell proliferation, cell migration, cell survival, or subcutaneous tumor cell xenograft growth *in vivo* [12]. Nevertheless, it appears essential that these data be replicated, given the design limitations of this type of functional experiment. In particular, these studies used only siRNAs to repress LINC01116 expression, without specifying the concentration at which the siRNAs were used in their experiments. Given the proven high rate of off‐target effects associated with RNA interference due notably to ‘miRNA‐like’‐mediated effects [26, 27], we decided to use CRISPRi to perform loss‐of‐function studies of LINC01116, as this approach produces far fewer side effects than siRNAs [28]. We optimized the CRISPRi approach in a number of ways [16], selecting the most efficient clone of dCas9‐KRAB‐MeCP2‐transduced A549 cells and the most effective guides that target the lncRNA promoter. In parallel, we also selected two siRNAs out of seven tested, capable of knocking down LINC01116 by 80% at a concentration as low as 10 nm. Both loss‐of‐function approaches failed to demonstrate statistically significant effects on *in vitro* cellular processes involved in tumor development such as cell migration or cell proliferation as well as on *in vivo* growth of LUAD cell xenografts in immune‐compromised mice. Furthermore, gain‐of‐function studies confirmed the lack of effects of LINC01116 overexpression on LUAD cell proliferation and migration. Overall, with the exception of the dysregulation of a subset of 74 genes in LUAD xenografts repressed for LINC01116 expression, we conclude that LINC01116 has no marked direct effect on LUAD cancer cell aggressiveness. Further work will be required to determine a possibly more subtle regulatory function, as is suggested for a large number of noncoding RNAs [29, 30].

In other cancers, several reports have highlighted the implication of LINC01116 in tumor development notably in the context of gliomas. LINC01116 has been proposed to act as an enhancer‐associated lncRNA and has been renamed HOXDeRNA in gliomas, in which it plays a role in astrocyte transformation through a nuclear mechanism that modifies chromatin topology with the formation of loops that derepress the distant HOXD/miR‐10b locus [31, 32]. Another study showed that the repression of LINC01116 in glioma cell lines led to a decrease in VEGFA production and, consequently, supernatants from these cells exhibited a strong negative effect on tubule formation of HUVECs, compared with control supernatants [33], suggesting the existence of a dialog between tumor cells and the vascular counterpart of the tumor stroma. Interestingly, we also observed inhibition of VEGFA among the set of DEGs in tumor xenografts repressed for LINC01116 expression. In addition, functional annotation of DEGs suggests that these genes are significantly associated with Gene Ontology Disease or function terms consistent with decreased invasion and angiogenesis.

Our results reorient the function of this lncRNA by showing that its expression in LUADs is not localized predominantly in tumor epithelial cells. Accordingly, in normal lung tissues, several publicly available scRNA‐seq datasets show that the expression of LINC01116 is largely restricted to LECs. In addition, RNA‐FISH performed on LUAD biopsies indicated that the lymphatic endothelium is an important source of LINC01116 expression in tumors, and primary LEC cultures express higher levels of LINC01116 when compared to LUAD cell lines. Finally, we found a positive correlation between the expression level of LINC01116 and that of PROX1 in both local and TCGA cohorts. It is therefore tempting to hypothesize that the association of LINC01116 high expression with poor prognosis and high recurrence rate in LUAD could be linked, at least in part, to a higher lymphatic vascularization in the tumors. Interestingly, lymphatic vessels can play a dual role in the tumor microenvironment. Indeed, LECs have unique immunomodulatory properties that can promote or inhibit antitumor immune responses. On one hand, a high density of lymphatic vessels and a high incidence of lymphovascular invasion are typically associated with lymph node metastasis favoring immunoevasion and reduced patient survival [34, 35, 36, 37]. Indeed, prior to nodal dissemination, the primary tumor modulates the microenvironment of the sentinel lymph node by secreting soluble factors or releasing extracellular vesicles that are transported by lymphatic vessels [38, 39]. Subsequently, lymph node metastases might have the potential to seed distant organs and be responsible for systemic metastases that current treatments fail to control [40, 41]. On the other hand, different molecular mechanisms involved in LEC‐mediated improvement of antitumor immunity have been reported [42].

In solid tumors, the hypoxic microenvironment induces translocation of HIF into nuclei that control expression of VEGF family members and of the lymphatic transcription factor PROX1 [23, 43, 44, 45, 46]. Hypoxia induces the proliferation of lymphatic endothelial cells and promotes pathological lymphangiogenesis [47, 48]. Therefore, our data indicating that efficient LINC01116 KD is associated with reduced proliferation potential in primary LECs cultured under hypoxic condition suggest a possible role for this lncRNA in pathological lymphangiogenesis in solid tumors. In addition, we observed, in two independent LUAD cohorts, that a strong expression of LINC01116 is associated with a decrease in tumor immune infiltrate score. In this context, understanding the potential role played by LINC01116 in the crosstalk between LUAD cancer cells, LECs and immune cells and how this communication may shape the immune landscape in the tumor microenvironment is of particular interest and will require further investigations. Rather than subcutaneous xenografts of LUAD cells, consideration should be given to using a lung orthotopic implantation model that better reflects pathophysiological conditions and has already been used to study the dialog between LUAD cells, tumor‐associated neutrophils and lymphangiogenesis [49]. Such a model could be useful in deciphering the functional role of LINC01116 in this crosstalk, provided that the existence of a murine ortholog of this transcript would be demonstrated.

Our study has several limitations, in particular due to difficulties in obtaining primary LECs, their degree of purity as well as variations in the efficacy of siRNA‐mediated gene knockdown depending on the donor, which could be related to the high variability in the doubling time of LEC cultures. These constraints prevented us from further functional studies on LEC cultures to better understand the function and mechanism of action of LINC01116, which should be studied in more complex *in vivo* settings as discussed above.

## 5 Conclusions

In conclusion, our data provide strong evidence for the association of LINC01116 with LECs. The function of this lncRNA is still to be further clarified, particularly *in vivo*, but our results suggest that the correlation between increased LINC01116 expression and high recurrence rate in LUAD could be explained by the specificity of expression of this lncRNA as a marker of lymphangiogenesis in tumors.

## Conflict of interest

The authors declare no conflict of interest.

## Author contributions

R.R. and B.M. conceived and designed the study and supervised the entire work. M.G.‐I. and R.R. designed and conducted the experiments. M.G.‐I., T.M., and B.S. conducted the TCGA, local cohort, and single‐cell RNA sequencing datasets analysis. L.C., S.C., C.H., G.L., and L.G.R. provided technical support for *in vitro* and *in vivo* experiments. I.M. and H.P. managed and provided the sample collection of patients' local cohort from Nice University Hospital. M.G.‐I., V.G., B.M., and R.R. wrote the manuscript. R.R., B.M., and V.G. provided the financial support. All authors revised the manuscript.

## Supporting information


**Table S1.** Selection of lncRNA candidates.

**Fig. S1.** Altering LINC01116 expression does not affect the proliferative and migratory properties of LUAD tumor cells.
**Fig. S2.** LINC01116 CRISPRi efficiency in LUAD xenografts.
**Fig. S3.** LINC01116 is expressed in tumor‐associated endothelial cells in LUADs.
**Fig. S4.** Association between tumor stroma composition and LINC01116 expression in LUAD cohorts.
**Fig. S5.** LINC01116 is expressed in lymphatic endothelial cells in the human lung.
**Fig. S6.** Differentially Expressed Genes in LEC repressed for LINC01116 expression.

## Data Availability

All transcriptomic datasets (bulk RNA‐seq and microarrays) will be submitted to the Gene Expression Omnibus (GEO) database prior to publication and will be available upon request. Previous data of LUAD local cohort microarray are accessible in GEO at GSE117049 reference number. The public single‐cell RNA sequencing datasets are accessible in GEO at GSE136831 [19], GSE253013 [20], and GSE164829 [21] reference numbers.
