# Supplementary material for: LINC01116, a hypoxia‐lncRNA marker of pathological lymphangiogenesis and poor prognosis in lung adenocarcinoma
Source: Mol Oncol. 2025 Dec 9;20(4):962–80. doi: 10.1002/1878-0261.70175 (PMC13060655; doi:10.1002/1878-0261.70175)
Supplement: Supplementary file 1 — Table S1. Selection of lncRNA candidates. [file MOL2-20-962-s002.pdf]

Supplemental Table S1: selection of lncRNA candidates. Selection was based on the following criteria: LUAD hypoxic status (log2(intensity) > 6, Hx.pca.p.1-value < 0.001), prognosis in the local cohort (OS.chi2.pval < 0.05) and significant modulation in A549 LUAD cells cultured in 1% O2 for 24h (absolute log2(hypoxia/normoxia) > 0.5, log2(intensity) > 6 and adj. P-value<0.05).

					**Local cohort (57 LUAD & 11 healthy lungs)**				**A549: Hx *vs* Nx**		
**GeneName**	**SystematicName**	**Description**	**chr_coord**	**ProbeName**	**AveExpr**	**hypoxia.pca.p.1**	**hypoxia.pca.e.1**	**OS.chi2.pval**	**AveExpr**	**M_Hx-Nx**	**AdP.Val_Hx-Nx**
LINC01116	NR_040001	ref|Homo sapiens long intergenic non-protein (	hs|chr2:177494727-177494668	A_23_P302787	630	674E-06	-056	106E-02	615	053	293E-02
LOC645166	NR_027355	ref|Homo sapiens lymphocyte-specific protein	hs|chr1:148951509-148951568	A_32_P217655	1049	497E-06	-057	357E-01	865	-051	398E-02
LOC101929475	ENST00000450890	ref|PREDICTED: Homo sapiens uncharacterized	hs|chr10:33370678-33370737	A_19_P00321617	856	294E-06	-058	357E-01	750	077	157E-02
C14orf132	NM_001282463	ref|Homo sapiens chromosome 14 open readir	hs|chr14:96556334-96556393	A_19_P00321588	814	208E-05	054	477E-01	650	062	402E-02
VPS9D1-AS1	NR_036480	ref|Homo sapiens VPS9D1 antisense RNA 1 (VP	hs|chr16:89784514-89784573	A_33_P3294372	669	606E-08	-066	100E+00	746	-099	701E-03
LUCAT1	NR_103548	ref|Homo sapiens lung cancer associated trans	hs|chr5:90598902-90598843	A_21_P0004421	726	761E-05	-051	867E-01	794	167	245E-04
LOC100422737	ENST00000602934	gb|Homo sapiens cDNA FLJ41422 fis, clone BRH	hs|chr6:107218599-107218540	A_19_P00316479	658	310E-04	047	477E-01	703	071	196E-02
STAG3L4	NR_040585	ref|Homo sapiens stromal antigen 3-like 4 (pse	hs|chr7:66785323-66785382	A_33_P3373298	849	997E-06	-055	357E-01	686	-058	250E-02
LOC100506990	NR_040092	ref|Homo sapiens uncharacterized LOC100506	hs|chr8:12388477-12388536	A_21_P0013533	774	475E-06	057	477E-01	636	054	392E-02
lnc-PRAGMIN.1-3	lnc-PRAGMIN.1-3:3	linc|LNCipedia lincRNA (lnc-PRAGMIN.1-3), linc	hs|chr8:8085049-8084990	A_21_P0005794	803	894E-05	050	867E-01	670	064	293E-02
LINC00894	NR_027456	ref|Homo sapiens long intergenic non-protein (	hs|chrX:149115124-149115183	A_19_P00320989	706	312E-04	047	719E-02	684	056	445E-02
XLOC_l2_015760	TCONS_l2_00030536	linc|BROAD Institute lincRNA (XLOC_l2_01576(	hs|chrX:73498033-73497974	A_19_P00315601	1168	204E-04	047	209E-01	988	085	106E-02
DNAJA1P5	AF395440	gb|Homo sapiens HEJ1 mRNA, complete cds. [A	hs|chr1:102359662-102359720	A_33_P3807268	1091	249E-04	-047	143E-01	709	-061	273E-02
SERTAD4-AS1	NR_024337	ref|Homo sapiens SERTAD4 antisense RNA 1 (S	hs|chr1:210404928-210404869	A_32_P207169	859	342E-05	053	867E-01	690	059	272E-02
SNHG1	NR_003098	ref|Homo sapiens small nucleolar RNA host ger	hs|chr11:62619632-62619573	A_24_P785293	1112	268E-09	-070	706E-01	852	-071	185E-02
DCAF13P3	NR_027642	ref|Homo sapiens DDB1 and CUL4 associated f	hs|chr15:51238166-51238225	A_33_P3420426	898	000E+00	-072	357E-01	676	-100	130E-02
PEAK1	ENST00000560626	ens|pseudopodium-enriched atypical kinase 1	hs|chr15:77657560-77638950	A_21_P0008856	850	227E-04	047	867E-01	669	059	166E-02
LOC727751	NR_102747	ref|Homo sapiens golgin A2 pseudogene (LOC7	hs|chr15:82765395-82765336	A_33_P3407618	778	624E-05	051	209E-01	767	055	284E-02
ATP2A1-AS1	NR_046287	ref|Homo sapiens ATP2A1 antisense RNA 1 (AT	hs|chr16:28890338-28890279	A_21_P0014321	785	659E-04	-044	867E-01	788	-136	378E-04
SLC6A10P	NR_003083	ref|Homo sapiens solute carrier family 6 (neuro	hs|chr16:32890762-32890621	A_24_P15621	756	216E-04	-048	706E-01	609	088	862E-03
UBE2MP1	NR_002837	ref|Homo sapiens ubiquitin-conjugating enzym	hs|chr16:34404117-34404058	A_24_P239017	872	122E-05	-055	357E-01	702	-054	259E-02
lnc-CLEC18B-1	lnc-CLEC18B-1:1	linc|LNCipedia lincRNA (lnc-CLEC18B-1), lincRN	hs|chr16:74411482-74411423	A_21_P0009134	620	319E-04	046	867E-01	685	055	380E-02
UNK	NR_038131	ref|Homo sapiens unkempt family zinc finger (U	hs|chr17:73790093-73790152	A_21_P0000715	917	779E-07	-061	100E+00	763	-059	151E-02
ENST00000431702	ENST00000431702	ens|phosphoglycerate kinase 1, pseudogene 2	hs|chr19:12671224-12671283	A_21_P0011675	1099	355E-08	-067	100E+00	918	226	442E-05
CEBPA-AS1	NR_026887	ref|Homo sapiens CEBPA antisense RNA 1 (hea	hs|chr19:33795725-33795784	A_21_P0009711	766	353E-04	046	477E-01	628	065	381E-02
LOC389033	NR_026740	ref|Homo sapiens placenta-specific 9 pseudoge	hs|chr2:130680494-130680435	A_33_P3283601	1077	291E-08	067	100E+00	960	-073	212E-02
LOC728323	ENST00000416103	Unknown	hs|chr2:243037063-243037122	A_32_P180971	1174	626E-05	051	209E-01	1014	069	791E-03
LINC00152	NR_024204	ref|Homo sapiens long intergenic non-protein (	hs|chr2:87820743-87820802	A_24_P273143	1073	418E-06	-058	706E-01	972	079	444E-03
FLJ16779	NR_024389	ref|Homo sapiens uncharacterized LOC100192	hs|chr20:61892908-61892967	A_19_P00322224	653	611E-04	-044	100E+00	641	081	851E-03
ENST00000450472	ENST00000450472	ens|glyceraldehyde-3-phosphate dehydrogena	hs|chr21:30595041-30595100	A_21_P0012174	1212	000E+00	-084	100E+00	1158	103	142E-03
MIF-AS1	NR_038911	ref|Homo sapiens MIF antisense RNA 1 (MIF-A	hs|chr22:24236690-24236631	A_33_P3351894	926	441E-04	-045	100E+00	978	147	378E-04
ENST00000512915	ENST00000512915	Unknown	hs|chr4:103340480-103340421	A_19_P00321461	730	111E-04	-050	357E-01	782	111	130E-03
ENST00000612111	ENST00000612111	Unknown	hs|chr6:161178501-161176480	A_21_P0013117	761	239E-04	047	477E-01	780	054	251E-02
XLOC_l2_013460	TCONS_l2_00025976	linc|BROAD Institute lincRNA (XLOC_l2_01346(	hs|chr7:065224954-065225013	A_21_P0013234	805	000E+00	-072	357E-01	691	-075	989E-03
TPI1P2	NR_002187	ref|Homo sapiens triosephosphate isomerase 1	hs|chr7:128696929-128696988	A_24_P358328	1209	000E+00	-077	143E-01	939	092	648E-03
LOC641746	NR_033245	ref|Homo sapiens glycine cleavage system pro	hs|chr7:64043343-64043402	A_32_P57702	1024	143E-05	-055	100E+00	805	-063	295E-02
ENST00000403118	ENST00000403118	Unknown	hs|chr7:75465240-75465299	A_21_P0013239	750	000E+00	-072	143E-01	701	-055	404E-02
XLOC_l2_015451	TCONS_l2_00030147	linc|BROAD Institute lincRNA (XLOC_l2_015451	hs|chrX:12844957-12845016	A_21_P0013753	1378	153E-07	-063	100E+00	1086	-092	336E-03
LOC101928803	XR_251551	ref|PREDICTED: Homo sapiens uncharacterized	unmapped	A_21_P0012654	1078	420E-06	058	867E-01	928	144	503E-04
LOC115110	NR_037844	ref|Homo sapiens uncharacterized LOC115110	hs|chr1:2481539-2481480	A_33_P3242820	869	266E-04	047	598E-04	620	-006	848E-01
ZEB1-AS1	NR_024284	ref|Homo sapiens ZEB1 antisense RNA 1 (ZEB1-	hs|chr10:31605526-31605467	A_19_P00811048	646	508E-04	-045	444E-02	601	-006	851E-01
FLJ46906	NR_033896	ref|Homo sapiens uncharacterized LOC441172	hs|chr6:139018042-139018101	A_19_P00320665	672	751E-06	-056	444E-02	594	-001	971E-01
MAPKAPK5-AS1	NR_015404	ref|Homo sapiens MAPKAPK5 antisense RNA 1	hs|chr12:112278006-112277947	A_23_P99204	793	790E-04	-044	444E-02	593	038	343E-01
lnc-C15orf2-2	lnc-C15orf2-2:9	linc|LNCipedia lincRNA (lnc-C15orf2-2), lincRNA	hs|chr15:24779473-24779532	A_21_P0008595	601	597E-04	044	191E-02	581	-002	967E-01
CIRBP	ENST00000621399	ens|cold inducible RNA binding protein [Source	hs|chr19:1274007-1274066	A_23_P377616	916	934E-04	043	191E-02	601	025	311E-01
TRAPPC13	NR_003545	ref|Homo sapiens trafficking protein particle co	hs|chr5:64926285-64926344	A_33_P3251821	692	696E-04	-044	444E-02	573	000	997E-01
TOB2P1	NR_002936	ref|Homo sapiens transducer of ERBB2, 2 pseu	hs|chr6:28183182-28183123	A_33_P3348011	634	444E-04	-045	444E-02	575	-018	440E-01
LOC100128317	NR_126025	ref|Homo sapiens uncharacterized LOC100128	hs|chr7:81205823-81205764	A_19_P00318978	675	759E-05	-051	444E-02	579	008	777E-01
XLOC_l2_015033	TCONS_l2_00029054	linc|BROAD Institute lincRNA (XLOC_l2_01503	hs|chr9:132049335-132049394	A_21_P0013601	755	869E-06	056	598E-04	585	-005	896E-01
LOC389765	NR_029410	ref|Homo sapiens kinesin family member 27 ps	hs|chr9:88454999-88455058	A_23_P9280	877	615E-05	051	387E-03	651	-015	623E-01
LINC00086	NR_024359	ref|Homo sapiens long intergenic non-protein (	hs|chrX:134556222-134556281	A_33_P3294868	664	103E-05	-056	444E-02	648	026	244E-01
ENST00000560500	ENST00000560500	ens|nucleolar protein interacting with the FHA	hs|chr15:90827697-90827638	A_21_P0011387	987	160E-05	-055	477E-01	808	-047	436E-02
GMDS-AS1	NR_046229	ref|Homo sapiens GMDS antisense RNA 1 (hea	hs|chr6:2413216-2413275	A_21_P0004819	723	102E-04	050	477E-01	615	046	428E-02
LINC01138	NR_027468	ref|Homo sapiens long intergenic non-protein (	hs|chr1:143718585-143718526	A_21_P0000539	607	189E-05	-054	706E-01	587	032	139E-01
LINC00982	NR_015440	ref|Homo sapiens long intergenic non-protein (	hs|chr1:2977709-2977148	A_21_P0001460	643	386E-05	052	477E-01	561	010	721E-01
SFTA1P	NR_027082	ref|Homo sapiens surfactant associated 1, pse	hs|chr10:10834404-10834345	A_21_P0006968	912	267E-08	067	477E-01	573	008	786E-01
LINC00959	NR_034125	ref|Homo sapiens long intergenic non-protein (	hs|chr10:131862240-131862181	A_21_P0000642	830	720E-06	056	867E-01	587	-012	649E-01

FAM21EP	NR_038275	ref|Homo sapiens family with sequence similar	hs|chr10:51826458-51826399	A_24_P677642	629	365E-04	-046	706E-01	575	-004	892E-01
MIR4697HG	NR_024344	ref|Homo sapiens MIR4697 host gene (non-pro	hs|chr11:133766698-133766639	A_19_P00809440	672	931E-05	050	100E+00	606	-008	808E-01
LOC729732	NR_047662	ref|Homo sapiens uncharacterized LOC729732	hs|chr12:8543558-8543499	A_33_P3211793	735	223E-04	048	100E+00	632	047	634E-02
ARHGAP5-AS1	NR_027263	ref|Homo sapiens ARHGAP5 antisense RNA 1 (	hs|chr14:32545243-32545184	A_33_P3348362	720	220E-06	-058	357E-01	624	011	663E-01
lnc-MBIP-1	lnc-MBIP-1:1	linc|LNCipedia lincRNA (lnc-MBIP-1), lincRNA [l	hs|chr14:36738340-36738281	A_21_P0008422	668	149E-04	048	100E+00	571	-004	905E-01
KTN1-AS1	NR_027123	ref|Homo sapiens KTN1 antisense RNA 1 (KTN1	hs|chr14:56043938-56043879	A_19_P00808852	609	000E+00	-070	357E-01	651	-016	502E-01
LOC100288637	NR_038253	ref|Homo sapiens OTU deubiquitinase 7A pseu	hs|chr15:31065023-31065082	A_21_P0000739	698	000E+00	-074	100E+00	609	003	949E-01
GOLGA2P7	NR_027001	ref|Homo sapiens golgin A2 pseudogene 7 (GO	hs|chr15:84867734-84867675	A_23_P398275	1143	439E-05	052	477E-01	965	018	514E-01
LOC100128770	NR_047572	ref|Homo sapiens uncharacterized LOC100128	hs|chr16:3089072-3089131	A_33_P3376031	670	981E-05	050	867E-01	588	-006	850E-01
FENDRR	NR_036444	ref|Homo sapiens FOXF1 adjacent non-coding	hs|chr16:86521807-86521748	A_19_P00322655	743	333E-06	058	209E-01	574	-007	795E-01
lnc-NR1D1-1	lnc-NR1D1-1:1	linc|LNCipedia lincRNA (lnc-NR1D1-1), lincRNA	hs|chr17:38276890-38276831	A_21_P0009323	674	122E-04	-049	357E-01	606	-002	949E-01
TBX2-AS1	NR_125749	ref|Homo sapiens TBX2 antisense RNA 1 (TBX2	hs|chr17:59470886-59470827	A_21_P0009343	912	103E-04	050	867E-01	791	-005	910E-01
LINC00673	NR_036488	ref|Homo sapiens long intergenic non-protein	hs|chr17:70399580-70399521	A_19_P00319646	848	165E-04	-048	143E-01	730	-042	142E-01
SNHG20	NR_027058	ref|Homo sapiens small nucleolar RNA host ge	hs|chr17:75090443-75090502	A_32_P8529	700	368E-04	-046	100E+00	617	-004	943E-01
ANKRD20A5P	NR_040113	ref|Homo sapiens ankyrin repeat domain 20 fa	hs|chr18:14225703-14225762	A_32_P150086	759	271E-05	053	477E-01	657	000	999E-01
MIR4435-1HG	NR_024373	ref|Homo sapiens MIR4435-1 host gene (non-p	hs|chr2:112186945-112186886	A_21_P0012079	1264	172E-04	-048	867E-01	1189	016	494E-01
LOC285043	ENST00000421976	gb|Homo sapiens cDNA FLJ41940 fis, clone PEF	hs|chr2:30575212-30575271	A_19_P00320998	841	000E+00	072	209E-01	573	-004	896E-01
CYP1B1-AS1	NR_027252	ref|Homo sapiens CYP1B1 antisense RNA 1 (CY	hs|chr2:38408930-38408989	A_19_P00316135	632	602E-05	051	100E+00	568	-004	923E-01
BOLA3-AS1	NR_045637	ref|Homo sapiens BOLA3 antisense RNA 1 (hea	hs|chr2:74376098-74376157	A_21_P0001775	680	104E-06	-061	706E-01	559	042	891E-02
LINC01296	NR_122112	ref|Homo sapiens long intergenic non-protein	hs|chr22:16159014-16158955	A_21_P0014928	609	261E-04	-047	706E-01	677	013	667E-01
TTC28-AS1	NR_026962	ref|Homo sapiens TTC28 antisense RNA 1 (TTC	hs|chr22:28320848-28320907	A_19_P00323375	685	678E-05	-051	143E-01	562	-014	577E-01
XLOC_l2_009539	TCONS_l2_00018033	linc|BROAD Institute lincRNA (XLOC_l2_00953	hs|chr22:35590675-35590616	A_21_P0012298	689	451E-05	-052	357E-01	565	-005	908E-01
MIRLET7BHG	NR_027033	ref|Homo sapiens MIRLET7B host gene (non-p	hs|chr22:46509748-46509807	A_33_P3328360	976	263E-05	053	719E-02	930	-026	329E-01
CHKB-AS1	NR_021492	ref|Homo sapiens CHKB antisense RNA 1 (head	hs|chr22:51022107-51022166	A_33_P3394489	714	326E-04	-046	477E-01	628	007	835E-01
LINC00888	NR_038301	ref|Homo sapiens long intergenic non-protein	hs|chr3:183173698-183173757	A_21_P0000758	846	191E-04	-048	706E-01	698	-014	558E-01
XLOC_l2_010511	TCONS_l2_00019725	linc|BROAD Institute lincRNA (XLOC_l2_01051	hs|chr3:197339521-197339462	A_21_P0012487	621	725E-05	-051	357E-01	564	017	550E-01
lnc-CD83-3	lnc-CD83-3:3	linc|LNCipedia lincRNA (lnc-CD83-3), lincRNA [l	hs|chr6:14512375-14512434	A_21_P0004843	648	985E-05	050	719E-02	573	016	619E-01
CMAHP	NR_002174	ref|Homo sapiens cytidine monophospho-N-ac	hs|chr6:25081857-25081798	A_33_P3281572	833	488E-08	067	100E+00	581	-006	870E-01
LINC00472	NR_121612	ref|Homo sapiens long intergenic non-protein	hs|chr6:72126221-72126162	A_19_P00321203	701	727E-06	056	209E-01	679	006	867E-01
LHFPL3-AS2	NR_027374	ref|Homo sapiens LHFPL3 antisense RNA 2 (LH	hs|chr7:104535162-104535103	A_33_P3692756	809	853E-08	066	477E-01	612	000	996E-01
LOC100506860	NR_109780	ref|Homo sapiens uncharacterized LOC100506	hs|chr7:130606643-130606702	A_19_P00316340	649	586E-05	-051	143E-01	706	-075	628E-02
LINC-PINT	NR_109854	ref|Homo sapiens long intergenic non-protein	hs|chr7:130737303-130737244	A_19_P00322977	962	561E-05	051	209E-01	734	042	251E-01
LOC441204	NR_015364	ref|Homo sapiens uncharacterized LOC441204	hs|chr7:26533624-26533683	A_19_P00318694	659	730E-05	051	867E-01	582	004	937E-01
MGC72080	NR_002822	ref|Homo sapiens MGC72080 pseudogene (M	hs|chr7:97596037-97595978	A_33_P3280400	834	132E-04	-049	867E-01	646	-039	818E-02
SNHG6	NR_002599	ref|Homo sapiens small nucleolar RNA host ge	hs|chr8:67834908-67834849	A_19_P00322948	1284	343E-05	-053	100E+00	913	018	453E-01
XLOC_l2_000339	TCONS_l2_00000470	linc|BROAD Institute lincRNA (XLOC_l2_00033	hs|chr1:107383778-107407857	A_21_P0010540	611	476E-05	052	867E-01	570	-002	952E-01
ATP1A1-AS1	NR_027645	ref|Homo sapiens ATP1A1 antisense RNA 1 (AT	hs|chr1:116941681-116941622	A_21_P0000543	764	356E-04	046	477E-01	783	026	265E-01
ENST00000441809	ENST00000441809	Unknown	hs|chr1:13986185-13986126	A_21_P0001100	758	716E-04	044	100E+00	575	023	338E-01
ANKRD20A12P	NR_046228	ref|Homo sapiens ankyrin repeat domain 20 fa	hs|chr1:142713108-142713049	A_32_P109604	737	491E-04	045	477E-01	679	020	410E-01
LINC00869	NR_111950	ref|Homo sapiens long intergenic non-protein	hs|chr1:144465067-144465008	A_33_P3282740	772	360E-04	046	209E-01	590	022	497E-01
ENST00000427962	ENST00000427962	Unknown	hs|chr1:150853956-150853897	A_21_P0010655	1212	841E-05	-050	143E-01	869	045	959E-02
lnc-MIB2-1	lnc-MIB2-1:5	linc|LNCipedia lincRNA (lnc-MIB2-1), lincRNA [l	hs|chr1:1542657-1542716	A_21_P0000910	870	352E-04	-046	357E-01	707	045	977E-02
lnc-SHE-1	lnc-SHE-1:1	ref|PREDICTED: Homo sapiens Src homology 2	hs|chr1:154442176-154442117	A_21_P0001742	804	313E-08	067	477E-01	587	-018	675E-01
MST1P2	NR_027504	ref|Homo sapiens macrophage stimulating 1 (h	hs|chr1:16974401-16974460	A_21_P0010692	710	810E-05	050	209E-01	621	025	297E-01
MST1L	NM_001271733	ref|Homo sapiens macrophage stimulating 1-li	hs|chr1:17082031-17081972	A_23_P340376	637	690E-04	044	706E-01	572	-038	410E-01
MROH3P	XR_171243	ref|PREDICTED: Homo sapiens maestro heat-li	hs|chr1:200935733-200935792	A_33_P3242793	700	604E-04	044	867E-01	592	-028	330E-01
LOC148709	NR_002929	ref|Homo sapiens actin pseudogene (LOC1487	hs|chr1:202843170-202843229	A_24_P84880	835	169E-05	-054	357E-01	715	043	815E-02
SNRPD2P2	NR_033826	ref|Homo sapiens small nuclear ribonucleopro	hs|chr1:231611762-231611703	A_33_P3384900	783	574E-07	-062	357E-01	614	-012	646E-01
ZNF436-AS1	NR_033690	ref|Homo sapiens ZNF436 antisense RNA 1 (ZN	hs|chr1:23698088-23698147	A_23_P362228	751	302E-04	-047	143E-01	610	041	265E-01
TCEB3-AS1	NR_038280	ref|Homo sapiens TCEB3 antisense RNA 1 (TCE	hs|chr1:24086931-24086872	A_21_P0000748	826	302E-05	053	867E-01	642	022	426E-01
lnc-AKT3-2	lnc-AKT3-2:1	linc|LNCipedia lincRNA (lnc-AKT3-2), lincRNA [l	hs|chr1:244210793-244210583	A_21_P0001652	619	151E-04	049	867E-01	585	004	916E-01
HNRNPU-AS1	NR_026778	ref|Homo sapiens HNRNPU antisense RNA 1 (H	hs|chr1:245004000-245003941	A_33_P3380161	1053	300E-04	047	706E-01	713	-014	754E-01
KDM4A-AS1	NR_033827	ref|Homo sapiens KDM4A antisense RNA 1 (KD	hs|chr1:44168387-44168328	A_33_P3334384	600	118E-04	-049	357E-01	596	006	849E-01
XLOC_l2_000217	TCONS_l2_00000291	linc|BROAD Institute lincRNA (XLOC_l2_00021	hs|chr1:55354884-55354943	A_21_P0010529	608	223E-05	054	477E-01	587	002	943E-01
LOC101926944	NR_110627	ref|Homo sapiens uncharacterized LOC101926	hs|chr1:60254330-60254389	A_21_P0000913	637	320E-05	052	209E-01	575	010	755E-01
ENO1-AS1	NR_038351	ref|Homo sapiens ENO1 antisense RNA 1 (ENO	hs|chr1:8939881-8939940	A_21_P0000775	601	188E-04	-048	706E-01	557	005	887E-01
PERM1	NM_001291366	ref|Homo sapiens PPARGC1 and ESRR induced	hs|chr1:911288-911229	A_23_P135742	695	596E-04	044	706E-01	591	011	711E-01
LINC01057	NR_104131	ref|Homo sapiens long intergenic non-protein	hs|chr1:95145116-95145057	A_21_P0010633	694	107E-04	049	867E-01	583	-005	881E-01
LOC101928241	NR_110693	ref|Homo sapiens uncharacterized LOC101928	hs|chr1:96719691-96719632	A_21_P0001158	648	804E-04	044	719E-02	586	008	796E-01
lnc-NDST2-3	lnc-NDST2-3:1	linc|LNCipedia lincRNA (lnc-NDST2-3), lincRNA	hs|chr10:075540630-075540571	A_21_P0007004	682	277E-06	-059	100E+00	606	-009	774E-01
SH3PXD2A-AS1	NR_038940	ref|Homo sapiens SH3PXD2A antisense RNA 1	hs|chr10:105514963-105515022	A_21_P0000846	618	879E-04	-043	100E+00	579	003	927E-01
lnc-C10orf31-7	lnc-C10orf31-7:2	linc|LNCipedia lincRNA (lnc-C10orf31-7), lincRN	hs|chr10:10828618-10826877	A_21_P0006971	640	545E-04	044	706E-01	554	-005	867E-01

ENST00000420825	ENST00000420825	Unknown	hs|chr10:11012652-11012711	A_21_P0006653	651	151E-05	054	477E-01	564	-003	924E-01
CASC2	NR_026939	ref|Homo sapiens cancer susceptibility candida	hs|chr10:119969564-119969623	A_33_P3213362	648	289E-04	047	867E-01	555	004	904E-01
LOC399815	NR_027282	ref|Homo sapiens chromosome 10 open readir	hs|chr10:124648065-124648124	A_33_P3607359	616	000E+00	-079	357E-01	625	-016	530E-01
CAMK1D	ENST00000619168	ens|calcium/calmodulin-dependent protein kir	hs|chr10:12875863-12875922	A_33_P3464555	1032	102E-04	050	100E+00	801	028	386E-01
LINC00202-1	NR_026795	ref|Homo sapiens long intergenic non-protein	hs|chr10:27220209-27220150	A_21_P0010860	704	763E-05	051	209E-01	679	009	745E-01
ENST00000432530	ENST00000432530	ref|PREDICTED: Homo sapiens zinc finger prote	hs|chr10:38064440-38064381	A_21_P0006748	658	480E-05	052	719E-02	567	006	841E-01
ZNF37BP	NR_026777	ref|Homo sapiens zinc finger protein 37B, pseu	hs|chr10:43009403-43009344	A_33_P3296858	788	332E-05	053	477E-01	711	016	550E-01
XLOC_l2_001448	TCONS_l2_00002856	linc|BROAD Institute lincRNA (XLOC_l2_001448	hs|chr10:5668390-5668449	A_21_P0010802	616	406E-04	-045	357E-01	604	-009	733E-01
lnc-BICC1-1	lnc-BICC1-1:1	linc|LNCipedia lincRNA (lnc-BICC1-1), lincRNA [	hs|chr10:60227284-60227343	A_21_P0006874	657	461E-04	045	209E-01	565	-002	951E-01
BMS1P4	NR_026592	ref|Homo sapiens BMS1 pseudogene 4 (BMS1F	hs|chr10:75477833-75477774	A_33_P3308497	628	851E-07	-061	706E-01	605	-010	720E-01
XLOC_l2_002033	TCONS_l2_00003643	linc|BROAD Institute lincRNA (XLOC_l2_002033	hs|chr10:81526056-81505786	A_21_P0010890	603	984E-04	-043	357E-01	553	000	996E-01
MBL1P	NR_002724	ref|Homo sapiens mannose-binding lectin (pro	hs|chr10:81682676-81682735	A_24_P169634	760	659E-08	066	477E-01	580	-013	587E-01
HECTD2-AS1	NR_024467	ref|Homo sapiens HECTD2 antisense RNA 1 (HI	hs|chr10:93067120-93067061	A_33_P3222788	823	261E-06	059	477E-01	737	044	286E-01
COLCA1	NM_207429	ref|Homo sapiens colorectal cancer associated	hs|chr11:111164321-111164262	A_33_P3356935	768	150E-05	055	100E+00	561	-003	924E-01
ST3GAL4-AS1	NR_033839	ref|Homo sapiens ST3GAL4 antisense RNA 1 (h	hs|chr11:126211673-126211614	A_21_P0000607	716	117E-04	-049	867E-01	621	-036	110E-01
LOC283177	NR_033852	ref|Homo sapiens uncharacterized LOC283177	hs|chr11:134352564-134352623	A_21_P0007307	648	276E-04	047	100E+00	561	-014	577E-01
LOC283177	NR_033852	ref|Homo sapiens uncharacterized LOC283177	hs|chr11:134374887-134374946	A_19_P00320614	628	626E-04	044	357E-01	616	027	499E-01
lnc-CDKN1C-1	lnc-CDKN1C-1:1	linc|LNCipedia lincRNA (lnc-CDKN1C-1), lincRN	hs|chr11:2900693-2900634	A_21_P0007313	613	154E-04	049	209E-01	577	012	669E-01
ENST00000511677	ENST00000511677	gb|Homo sapiens cDNA FLJ34677 fis, clone LIV	hs|chr11:67658752-67658811	A_21_P0007126	702	500E-04	045	867E-01	584	014	577E-01
LOC101928837	NR_120561	ref|Homo sapiens uncharacterized LOC101928	hs|chr11:76493751-76493692	A_32_P28223	683	108E-04	-050	867E-01	734	046	505E-02
lnc-ODZ4-2	lnc-ODZ4-2:1	linc|LNCipedia lincRNA (lnc-ODZ4-2), lincRNA [l	hs|chr11:79828024-79827965	A_21_P0007377	629	121E-05	055	477E-01	560	-005	872E-01
RAB30-AS1	NR_038903	ref|Homo sapiens RAB30 antisense RNA 1 (hea	hs|chr11:82783779-82783838	A_21_P0007250	841	612E-04	-044	357E-01	639	006	842E-01
LOC440028	NR_033972	ref|Homo sapiens uncharacterized LOC440028	hs|chr11:9776384-9776325	A_33_P3833256	605	000E+00	-076	706E-01	608	022	484E-01
XLOC_l2_003293	TCONS_l2_00006125	linc|BROAD Institute lincRNA (XLOC_l2_003293	hs|chr12:009552468-009552409	A_21_P0011084	730	158E-04	-049	143E-01	592	-029	216E-01
EP400NL	NR_003290	ref|Homo sapiens EP400 N-terminal like (EP40	hs|chr12:132610650-132610709	A_33_P3258953	660	717E-04	-044	100E+00	571	-012	642E-01
RHNO1	NM_001257097	ref|Homo sapiens RAD9-HUS1-RAD1 interactin	hs|chr12:2998466-2998525	A_33_P3263193	925	876E-04	-043	706E-01	647	-033	154E-01
DDX11-AS1	NR_038927	ref|Homo sapiens DDX11 antisense RNA 1 (DD	hs|chr12:31226147-31213664	A_21_P0007523	642	838E-08	-066	100E+00	580	-019	510E-01
lnc-C12orf68-1	lnc-C12orf68-1:1	linc|LNCipedia lincRNA (lnc-C12orf68-1), lincRN	hs|chr12:48615810-48619721	A_21_P0007551	621	823E-04	043	477E-01	573	002	965E-01
TMEM198B	NR_036476	ref|Homo sapiens transmembrane protein 198	hs|chr12:56229970-56230029	A_33_P3259557	1025	104E-06	061	477E-01	729	026	351E-01
ENST00000551082	ENST00000551082	Unknown	hs|chr12:76964495-77009090	A_21_P0011051	602	641E-04	-044	143E-01	587	000	992E-01
LINC00346	NR_027701	ref|Homo sapiens long intergenic non-protein	hs|chr13:111521859-111521800	A_23_P368909	657	716E-04	-044	143E-01	565	-020	485E-01
XLOC_l2_003877	TCONS_l2_00007042	linc|BROAD Institute lincRNA (XLOC_l2_003877	hs|chr13:114965887-114988000	A_21_P0011166	616	200E-04	047	477E-01	578	009	746E-01
ANKRD20A9P	NR_027995	ref|Homo sapiens ankyrin repeat domain 20 fa	hs|chr13:19415809-19415750	A_21_P0012182	913	441E-04	045	867E-01	666	016	502E-01
ENST00000441430	ENST00000441430	ens|long intergenic non-protein coding RNA 36	hs|chr13:39152937-39152996	A_21_P0008051	718	796E-04	044	100E+00	615	023	303E-01
UBAC2-AS1	NR_036531	ref|Homo sapiens UBAC2 antisense RNA 1 (UB	hs|chr13:99848687-99848628	A_21_P0000659	659	295E-04	-047	357E-01	619	-014	685E-01
SLC25A21-AS1	NR_033240	ref|Homo sapiens SLC25A21 antisense RNA 1 (	hs|chr14:37642565-37642624	A_32_P84454	621	108E-04	-049	143E-01	574	003	935E-01
ENST00000605130	ENST00000605130	Unknown	hs|chr14:69329176-69329235	A_21_P0011252	753	000E+00	-074	357E-01	599	-038	193E-01
LINC01220	ENST00000558575	ens|long intergenic non-protein coding RNA 12	hs|chr14:75763169-75763228	A_33_P3388080	658	673E-04	-044	357E-01	575	020	362E-01
lnc-GALC-3	lnc-GALC-3:4	linc|LNCipedia lincRNA (lnc-GALC-3), lincRNA [l	hs|chr14:88039919-88039860	A_21_P0008473	694	360E-04	046	477E-01	577	-015	676E-01
GOLGA8M	NM_001282468	ref|Homo sapiens golgin A8 family, member M	hs|chr15:28634538-28634597	A_24_P264166	651	911E-04	-043	706E-01	587	-016	654E-01
lnc-ZNF609-4	lnc-ZNF609-4:1	linc|LNCipedia lincRNA (lnc-ZNF609-4), lincRNA	hs|chr15:65016842-65016901	A_21_P0008644	602	509E-04	045	209E-01	589	-001	982E-01
UBL7-AS1	NR_038448	ref|Homo sapiens UBL7 antisense RNA 1 (head	hs|chr15:74773446-74773505	A_21_P0000808	694	124E-06	-060	357E-01	585	002	949E-01
LINC00597	NR_026813	ref|Homo sapiens long intergenic non-protein	hs|chr15:77516360-77516301	A_23_P54447	780	574E-05	051	867E-01	629	004	910E-01
lnc-PEAK1.1-1	lnc-PEAK1.1-1:3	linc|LNCipedia lincRNA (lnc-PEAK1.1-1), lincRN	hs|chr15:77657555-77649433	A_21_P0008857	833	657E-05	050	209E-01	648	039	132E-01
LOC440300	NR_033738	ref|Homo sapiens chondroitin sulfate proteogl	hs|chr15:84868369-84868428	A_21_P0011336	709	816E-04	043	867E-01	759	007	847E-01
LINC00924	NR_027133	ref|Homo sapiens long intergenic non-protein	hs|chr15:95997006-95997065	A_21_P0008684	615	203E-04	048	100E+00	589	-016	590E-01
LMF1	NR_036442	ref|Homo sapiens lipase maturation factor 1 (L	hs|chr16:1025821-1025762	A_33_P3883985	747	197E-05	054	867E-01	608	-017	611E-01
ENST00000566787	ENST00000566787	ens|long intergenic non-protein coding RNA 12	hs|chr16:10608890-10608831	A_21_P0009008	842	136E-05	055	477E-01	586	-013	599E-01
ENST00000443373	ENST00000443373	Unknown	hs|chr16:26596144-26596085	A_21_P0011460	679	468E-04	-045	706E-01	552	018	481E-01
CLUHP3	NR_024034	ref|Homo sapiens clustered mitochondria (clu	hs|chr16:31718232-31718291	A_23_P89062	863	279E-05	053	477E-01	639	040	699E-02
FTO-IT1	NR_103838	ref|Homo sapiens FTO intronic transcript 1 (no	hs|chr16:54074060-54074119	A_21_P0014327	805	289E-05	053	209E-01	635	002	961E-01
CRNDE	ENST00000501177	ens|colorectal neoplasia differentially expresse	hs|chr16:54957531-54954215	A_21_P0008884	868	356E-04	046	100E+00	648	012	632E-01
lnc-WFDC1-1	lnc-WFDC1-1:1	linc|LNCipedia lincRNA (lnc-WFDC1-1), lincRNA	hs|chr16:84376878-84376937	A_21_P0008980	653	267E-04	047	719E-02	567	020	578E-01
LOC101928710	NR_110848	ref|Homo sapiens uncharacterized LOC101928	hs|chr17:076613698-076613639	A_23_P250516	692	594E-04	-044	867E-01	564	-016	527E-01
FLJ35934	NR_104343	ref|Homo sapiens FLJ35934 (FLJ35934), long no	hs|chr17:18317599-18317658	A_23_P107432	608	196E-06	-059	706E-01	643	-008	765E-01
LOC100507002	NR_110801	ref|Homo sapiens uncharacterized LOC100507	hs|chr17:63106959-63107018	A_21_P0009265	770	924E-05	-050	100E+00	608	-003	939E-01
LINC01482	NR_110825	ref|Homo sapiens long intergenic non-protein	hs|chr17:66638824-66638883	A_21_P0009267	674	499E-05	-052	357E-01	731	-006	863E-01
XLOC_l2_005921	TCONS_l2_00011008	linc|BROAD Institute lincRNA (XLOC_l2_005921	hs|chr17:77801461-77801520	A_21_P0011560	642	692E-04	-044	143E-01	585	018	577E-01
BAIAP2-AS1	NR_026857	ref|Homo sapiens BAIAP2 antisense RNA 1 (he	hs|chr17:79003098-79003039	A_33_P3285156	1055	118E-04	049	867E-01	724	017	481E-01
lnc-PPP4R1-2	lnc-PPP4R1-2:4	linc|LNCipedia lincRNA (lnc-PPP4R1-2), lincRNA	hs|chr18:9318734-9318675	A_21_P0009521	648	764E-04	-044	100E+00	574	-005	881E-01
CIRBP-AS1	NR_027271	ref|Homo sapiens CIRBP antisense RNA 1 (CIRE	hs|chr19:1267531-1267472	A_33_P3851788	653	269E-04	-047	143E-01	648	-016	703E-01
SIGLEC17P	ENST00000614626	ens|sialic acid binding Ig-like lectin 17, pseudog	hs|chr19:51675776-51675835	A_33_P3309621	684	734E-04	044	209E-01	572	020	442E-01

<table>
<tr><td>ENST00000606966</td><td>ENST00000606966</td><td>ref|PREDICTED: Homo sapiens uncharacterized</td><td>hs|chr19:781073-781014</td><td>A_21_P0009734</td><td>701</td><td>735E-06</td><td>056</td><td>477E-01</td><td>794</td><td>048</td><td>136E-01</td></tr>
<tr><td>ENST00000431506</td><td>ENST00000431506</td><td>ens|CD8b molecule [Source:HGNC Symbol;Acc</td><td>hs|chr2:107119010-107121069</td><td>A_21_P0012018</td><td>634</td><td>264E-04</td><td>-047</td><td>100E+00</td><td>574</td><td>003</td><td>928E-01</td></tr>
<tr><td>lnc-SLC35F5-4</td><td>lnc-SLC35F5-4:1</td><td>linc|LNCipedia lincRNA (lnc-SLC35F5-4), lincRN</td><td>hs|chr2:114727135-114727076</td><td>A_19_P00316778</td><td>620</td><td>466E-04</td><td>045</td><td>706E-01</td><td>593</td><td>000</td><td>993E-01</td></tr>
<tr><td>LOC440910</td><td>NR_030728</td><td>ref|Homo sapiens uncharacterized LOC440910</td><td>hs|chr2:132056758-132056817</td><td>A_33_P3688869</td><td>609</td><td>235E-04</td><td>-047</td><td>706E-01</td><td>541</td><td>020</td><td>386E-01</td></tr>
<tr><td>XLOC_l2_009441</td><td>TCONS_l2_00018295</td><td>linc|BROAD Institute lincRNA (XLOC_l2_00944</td><td>hs|chr2:132440479-132440538</td><td>A_21_P0012328</td><td>615</td><td>102E-04</td><td>-050</td><td>867E-01</td><td>808</td><td>045</td><td>685E-02</td></tr>
<tr><td>ENST00000419201</td><td>ENST00000419201</td><td>ens|adenosylhomocysteinase pseudogene 4 [S</td><td>hs|chr2:139659396-139659337</td><td>A_21_P0011971</td><td>644</td><td>230E-04</td><td>-047</td><td>706E-01</td><td>603</td><td>015</td><td>517E-01</td></tr>
<tr><td>lnc-AC007405.7.1-1</td><td>lnc-AC007405.7.1-1:1</td><td>linc|LNCipedia lincRNA (lnc-AC007405.7.1-1), li</td><td>hs|chr2:171634855-171634914</td><td>A_21_P0002313</td><td>739</td><td>203E-04</td><td>048</td><td>477E-01</td><td>557</td><td>-003</td><td>926E-01</td></tr>
<tr><td>HAGLR</td><td>NR_110458</td><td>ref|Homo sapiens HOXD antisense growth-ass</td><td>hs|chr2:177038003-177037944</td><td>A_33_P3609033</td><td>883</td><td>117E-05</td><td>055</td><td>100E+00</td><td>585</td><td>008</td><td>777E-01</td></tr>
<tr><td>LOC101927027</td><td>NR_110205</td><td>ref|Homo sapiens uncharacterized LOC101927</td><td>hs|chr2:179298745-179298804</td><td>A_21_P0014089</td><td>781</td><td>253E-04</td><td>-047</td><td>867E-01</td><td>615</td><td>-003</td><td>935E-01</td></tr>
<tr><td>UBE2E3</td><td>ENST00000602837</td><td>ens|ubiquitin-conjugating enzyme E2E 3 [Sour</td><td>hs|chr2:181941111-181941170</td><td>A_19_P00323103</td><td>633</td><td>204E-08</td><td>-068</td><td>357E-01</td><td>604</td><td>-008</td><td>784E-01</td></tr>
<tr><td>IDH1-AS1</td><td>NR_046452</td><td>ref|Homo sapiens IDH1 antisense RNA 1 (IDH1</td><td>hs|chr2:209120795-209120854</td><td>A_21_P0014098</td><td>759</td><td>866E-04</td><td>-043</td><td>706E-01</td><td>627</td><td>-005</td><td>877E-01</td></tr>
<tr><td>ECEL1P2</td><td>NR_028501</td><td>ref|Homo sapiens endothelin converting enzyr</td><td>hs|chr2:233250541-233250482</td><td>A_33_P3610406</td><td>760</td><td>107E-05</td><td>055</td><td>867E-01</td><td>580</td><td>020</td><td>435E-01</td></tr>
<tr><td>XLOC_l2_008130</td><td>TCONS_l2_00014690</td><td>linc|BROAD Institute lincRNA (XLOC_l2_00813</td><td>hs|chr2:91910371-91910312</td><td>A_33_P3331426</td><td>659</td><td>368E-04</td><td>-046</td><td>143E-01</td><td>599</td><td>021</td><td>548E-01</td></tr>
<tr><td>ANKRD20A12P</td><td>NR_046228</td><td>ref|Homo sapiens ankyrin repeat domain 20 fa</td><td>hs|chr2:95461681-95461622</td><td>A_21_P0010769</td><td>759</td><td>329E-04</td><td>046</td><td>719E-02</td><td>950</td><td>006</td><td>837E-01</td></tr>
<tr><td>LOC442028</td><td>NR_037597</td><td>ref|Homo sapiens uncharacterized LOC442028</td><td>hs|chr2:95552708-95552649</td><td>A_21_P0011933</td><td>639</td><td>123E-05</td><td>-055</td><td>867E-01</td><td>590</td><td>-028</td><td>272E-01</td></tr>
<tr><td>LINC00342</td><td>NR_103734</td><td>ref|Homo sapiens long intergenic non-protein</td><td>hs|chr2:98088832-98088774</td><td>A_21_P0011934</td><td>976</td><td>152E-04</td><td>049</td><td>477E-01</td><td>594</td><td>-010</td><td>754E-01</td></tr>
<tr><td>ENST00000448494</td><td>ENST00000448494</td><td>ens|long intergenic non-protein coding RNA 34</td><td>hs|chr2:98088895-98088836</td><td>A_33_P3884179</td><td>871</td><td>782E-04</td><td>043</td><td>867E-01</td><td>614</td><td>029</td><td>312E-01</td></tr>
<tr><td>LINC01125</td><td>NR_038386</td><td>ref|Homo sapiens long intergenic non-protein</td><td>hs|chr2:98319403-98319462</td><td>A_24_P456723</td><td>805</td><td>122E-06</td><td>060</td><td>867E-01</td><td>582</td><td>004</td><td>895E-01</td></tr>
<tr><td>XLOC_l2_008599</td><td>TCONS_l2_00016224</td><td>linc|BROAD Institute lincRNA (XLOC_l2_00859</td><td>hs|chr20:25990491-26031378</td><td>A_21_P0012118</td><td>700</td><td>617E-04</td><td>044</td><td>477E-01</td><td>643</td><td>030</td><td>209E-01</td></tr>
<tr><td>lnc-KIAA1755-5</td><td>lnc-KIAA1755-5:7</td><td>gb|Homo sapiens hypothetical LOC388796, mR</td><td>hs|chr20:37049947-37049888</td><td>A_24_P608302</td><td>622</td><td>398E-06</td><td>-058</td><td>357E-01</td><td>568</td><td>011</td><td>750E-01</td></tr>
<tr><td>SNHG17</td><td>NR_027241</td><td>ref|Homo sapiens small nucleolar RNA host ge</td><td>hs|chr20:37055134-37055075</td><td>A_19_P00317793</td><td>856</td><td>575E-04</td><td>-045</td><td>357E-01</td><td>754</td><td>001</td><td>988E-01</td></tr>
<tr><td>ENST00000427691</td><td>ENST00000427691</td><td>ens|MIR646 host gene (non-protein coding) [S</td><td>hs|chr20:58729517-58755911</td><td>A_21_P0012155</td><td>603</td><td>656E-04</td><td>044</td><td>867E-01</td><td>570</td><td>-023</td><td>390E-01</td></tr>
<tr><td>ENST00000458422</td><td>ENST00000458422</td><td>ens|MIR646 host gene (non-protein coding) [S</td><td>hs|chr20:58859610-58859669</td><td>A_21_P0012156</td><td>731</td><td>130E-04</td><td>049</td><td>477E-01</td><td>584</td><td>-009</td><td>751E-01</td></tr>
<tr><td>lnc-C20orf197-3</td><td>lnc-C20orf197-3:13</td><td>linc|LNCipedia lincRNA (lnc-C20orf197-3), lincR</td><td>hs|chr20:58899541-58899597</td><td>A_21_P0009984</td><td>871</td><td>149E-05</td><td>055</td><td>706E-01</td><td>583</td><td>-001</td><td>988E-01</td></tr>
<tr><td>ANKRD20A11P</td><td>ENST00000429521</td><td>ens|ankyrin repeat domain 20 family, member</td><td>hs|chr21:15335073-15323443</td><td>A_21_P0012220</td><td>995</td><td>555E-05</td><td>051</td><td>209E-01</td><td>747</td><td>001</td><td>989E-01</td></tr>
<tr><td>LINC00649</td><td>NR_038883</td><td>ref|Homo sapiens long intergenic non-protein</td><td>hs|chr21:35303958-35304017</td><td>A_21_P0010308</td><td>775</td><td>519E-04</td><td>-045</td><td>357E-01</td><td>567</td><td>-005</td><td>886E-01</td></tr>
<tr><td>DSCR9</td><td>NR_026719</td><td>ref|Homo sapiens Down syndrome critical regi</td><td>hs|chr21:38592834-38592893</td><td>A_33_P3254996</td><td>607</td><td>817E-08</td><td>-066</td><td>357E-01</td><td>658</td><td>015</td><td>543E-01</td></tr>
<tr><td>MCM3AP-AS1</td><td>NR_002776</td><td>ref|Homo sapiens MCM3AP antisense RNA 1 (</td><td>hs|chr21:47671312-47671371</td><td>A_23_P256694</td><td>699</td><td>218E-04</td><td>-048</td><td>706E-01</td><td>602</td><td>003</td><td>938E-01</td></tr>
<tr><td>PRODH</td><td>ENST00000609229</td><td>ens|proline dehydrogenase (oxidase) 1 [Source</td><td>hs|chr22:20299201-20299260</td><td>A_21_P0012313</td><td>750</td><td>993E-04</td><td>043</td><td>706E-01</td><td>583</td><td>001</td><td>983E-01</td></tr>
<tr><td>GUSBP11</td><td>NR_024448</td><td>ref|Homo sapiens glucuronidase, beta pseudo</td><td>hs|chr22:24002114-24002055</td><td>A_24_P181998</td><td>1168</td><td>370E-04</td><td>046</td><td>867E-01</td><td>771</td><td>002</td><td>973E-01</td></tr>
<tr><td>CRYBB2P1</td><td>NR_033734</td><td>ref|Homo sapiens crystallin, beta B2 pseudoge</td><td>hs|chr22:25844171-25844230</td><td>A_21_P0012264</td><td>681</td><td>158E-05</td><td>-054</td><td>706E-01</td><td>621</td><td>000</td><td>998E-01</td></tr>
<tr><td>EMC3-AS1</td><td>NR_103821</td><td>ref|Homo sapiens EMC3 antisense RNA 1 (EM</td><td>hs|chr3:10046661-10046720</td><td>A_23_P384698</td><td>723</td><td>627E-04</td><td>-044</td><td>357E-01</td><td>609</td><td>004</td><td>907E-01</td></tr>
<tr><td>lnc-CHL1-1</td><td>lnc-CHL1-1:1</td><td>linc|LNCipedia lincRNA (lnc-CHL1-1), lincRNA [l</td><td>hs|chr3:109185-109357</td><td>A_21_P0003214</td><td>1122</td><td>634E-04</td><td>044</td><td>719E-02</td><td>1007</td><td>046</td><td>117E-01</td></tr>
<tr><td>ENST00000437591</td><td>ENST00000437591</td><td>ens|Fanconi anemia, complementation group</td><td>hs|chr3:11925567-11926470</td><td>A_21_P0012366</td><td>751</td><td>247E-07</td><td>-062</td><td>706E-01</td><td>705</td><td>-006</td><td>842E-01</td></tr>
<tr><td>XLOC_l2_009883</td><td>TCONS_l2_00018854</td><td>linc|BROAD Institute lincRNA (XLOC_l2_00988</td><td>hs|chr3:129839533-129839592</td><td>A_21_P0012393</td><td>881</td><td>236E-05</td><td>054</td><td>719E-02</td><td>645</td><td>072</td><td>154E-01</td></tr>
<tr><td>RNF7</td><td>NR_037702</td><td>ref|Homo sapiens ring finger protein 7 (RNF7),</td><td>hs|chr3:141461507-141461566</td><td>A_33_P3252915</td><td>626</td><td>278E-05</td><td>-052</td><td>357E-01</td><td>597</td><td>-017</td><td>511E-01</td></tr>
<tr><td>lnc-MECOM-1</td><td>lnc-MECOM-1:1</td><td>linc|LNCipedia lincRNA (lnc-MECOM-1), lincRN</td><td>hs|chr3:168746815-168746756</td><td>A_21_P0003189</td><td>615</td><td>967E-04</td><td>043</td><td>209E-01</td><td>585</td><td>-029</td><td>287E-01</td></tr>
<tr><td>XLOC_l2_010433</td><td>TCONS_l2_00019618</td><td>linc|BROAD Institute lincRNA (XLOC_l2_01043</td><td>hs|chr3:172143366-172143307</td><td>A_33_P3334575</td><td>987</td><td>178E-06</td><td>-060</td><td>100E+00</td><td>822</td><td>005</td><td>913E-01</td></tr>
<tr><td>MAP3K13</td><td>NR_038322</td><td>ref|Homo sapiens mitogen-activated protein k</td><td>hs|chr3:185009751-185009810</td><td>A_19_P00804711</td><td>739</td><td>329E-04</td><td>-046</td><td>143E-01</td><td>559</td><td>-004</td><td>900E-01</td></tr>
<tr><td>LINC01063</td><td>XR_246052</td><td>ref|PREDICTED: Homo sapiens long intergenic</td><td>hs|chr3:196358453-196358394</td><td>A_21_P0002923</td><td>654</td><td>444E-05</td><td>-052</td><td>706E-01</td><td>622</td><td>-043</td><td>992E-02</td></tr>
<tr><td>ENST00000493276</td><td>ENST00000493276</td><td>Unknown</td><td>hs|chr3:75440798-75436010</td><td>A_21_P0012448</td><td>654</td><td>163E-04</td><td>048</td><td>209E-01</td><td>642</td><td>-029</td><td>237E-01</td></tr>
<tr><td>LINC00312</td><td>NR_024065</td><td>ref|Homo sapiens long intergenic non-protein</td><td>hs|chr3:8615322-8615381</td><td>A_23_P166779</td><td>749</td><td>372E-05</td><td>052</td><td>477E-01</td><td>595</td><td>006</td><td>837E-01</td></tr>
<tr><td>LOC100507053</td><td>NR_037884</td><td>ref|Homo sapiens uncharacterized LOC100507</td><td>hs|chr4:100222364-100222423</td><td>A_21_P0000700</td><td>627</td><td>625E-04</td><td>044</td><td>100E+00</td><td>542</td><td>-008</td><td>793E-01</td></tr>
<tr><td>ENST00000509548</td><td>ENST00000509548</td><td>Unknown</td><td>hs|chr4:171293403-171293462</td><td>A_21_P0003432</td><td>953</td><td>667E-04</td><td>044</td><td>209E-01</td><td>832</td><td>006</td><td>873E-01</td></tr>
<tr><td>lnc-RP11-487E13.1.1-1</td><td>lnc-RP11-487E13.1.1-1:1</td><td>linc|LNCipedia lincRNA (lnc-RP11-487E13.1.1-1</td><td>hs|chr4:178222259-178141829</td><td>A_21_P0003803</td><td>620</td><td>723E-04</td><td>044</td><td>477E-01</td><td>578</td><td>-010</td><td>695E-01</td></tr>
<tr><td>SLED1</td><td>NR_003542</td><td>ref|Homo sapiens proteoglycan 3 pseudogene</td><td>hs|chr4:185719599-185719540</td><td>A_24_P927716</td><td>617</td><td>836E-04</td><td>-043</td><td>143E-01</td><td>566</td><td>-009</td><td>773E-01</td></tr>
<tr><td>lnc-SEL1L3-2</td><td>lnc-SEL1L3-2:1</td><td>linc|LNCipedia lincRNA (lnc-SEL1L3-2), lincRNA</td><td>hs|chr4:25554633-25554574</td><td>A_21_P0003731</td><td>732</td><td>185E-05</td><td>054</td><td>867E-01</td><td>557</td><td>003</td><td>919E-01</td></tr>
<tr><td>KLF3-AS1</td><td>NR_026804</td><td>ref|Homo sapiens KLF3 antisense RNA 1 (KLF3-</td><td>hs|chr4:38614426-38614367</td><td>A_23_P30163</td><td>696</td><td>270E-04</td><td>047</td><td>477E-01</td><td>582</td><td>020</td><td>438E-01</td></tr>
<tr><td>USP46-AS1</td><td>NR_125363</td><td>ref|Homo sapiens USP46 antisense RNA 1 (US</td><td>hs|chr4:53527427-53527486</td><td>A_32_P12327</td><td>666</td><td>113E-04</td><td>-049</td><td>100E+00</td><td>577</td><td>025</td><td>336E-01</td></tr>
<tr><td>FAM13A-AS1</td><td>NR_002806</td><td>ref|Homo sapiens FAM13A antisense RNA 1 (F</td><td>hs|chr4:89651092-89651151</td><td>A_33_P3218905</td><td>665</td><td>674E-04</td><td>044</td><td>477E-01</td><td>567</td><td>009</td><td>793E-01</td></tr>
<tr><td>ENST00000511422</td><td>ENST00000511422</td><td>Unknown</td><td>hs|chr5:120126414-120126473</td><td>A_21_P0004054</td><td>601</td><td>721E-04</td><td>-044</td><td>477E-01</td><td>558</td><td>002</td><td>949E-01</td></tr>
<tr><td>LOC729080</td><td>NR_033244</td><td>ref|Homo sapiens glycine cleavage system pro</td><td>hs|chr5:141276021-141275962</td><td>A_24_P66932</td><td>715</td><td>228E-05</td><td>-054</td><td>100E+00</td><td>569</td><td>-011</td><td>701E-01</td></tr>
<tr><td>XLOC_l2_012319</td><td>TCONS_l2_00023251</td><td>linc|BROAD Institute lincRNA (XLOC_l2_01231</td><td>hs|chr5:175344898-175344839</td><td>A_21_P0012929</td><td>614</td><td>472E-04</td><td>-045</td><td>706E-01</td><td>558</td><td>006</td><td>846E-01</td></tr>
<tr><td>LOC153684</td><td>NR_015447</td><td>ref|Homo sapiens uncharacterized LOC153684</td><td>hs|chr5:43045012-43045071</td><td>A_33_P3323760</td><td>911</td><td>348E-04</td><td>046</td><td>719E-02</td><td>757</td><td>017</td><td>512E-01</td></tr>
<tr><td>lnc-AC114947.1.1-2</td><td>lnc-AC114947.1.1-2:3</td><td>linc|LNCipedia lincRNA (lnc-AC114947.1.1-2), li</td><td>hs|chr5:43082098-43082157</td><td>A_21_P0004246</td><td>659</td><td>503E-04</td><td>045</td><td>477E-01</td><td>580</td><td>011</td><td>754E-01</td></tr>
<tr><td>ENST00000479830</td><td>ENST00000479830</td><td>ref|PREDICTED: Homo sapiens uncharacterized</td><td>hs|chr5:68339337-68339278</td><td>A_21_P0004139</td><td>753</td><td>790E-04</td><td>044</td><td>477E-01</td><td>578</td><td>-012</td><td>623E-01</td></tr>
<tr><td>LOC729506</td><td>NR_039984</td><td>ref|Homo sapiens uncharacterized LOC729506</td><td>hs|chr5:8457564-8452337</td><td>A_21_P0012891</td><td>826</td><td>203E-04</td><td>048</td><td>867E-01</td><td>597</td><td>000</td><td>995E-01</td></tr>
<tr><td>XLOC_l2_012870</td><td>TCONS_l2_00024608</td><td>linc|BROAD Institute lincRNA (XLOC_l2_01287</td><td>hs|chr6:010460190-010460131</td><td>A_21_P0013060</td><td>933</td><td>137E-06</td><td>-060</td><td>143E-01</td><td>657</td><td>-011</td><td>705E-01</td></tr>
<tr><td>BRD7P3</td><td>NR_002730</td><td>ref|Homo sapiens bromodomain containing 7</td><td>hs|chr6:118824846-118824905</td><td>A_33_P3313660</td><td>792</td><td>298E-07</td><td>063</td><td>867E-01</td><td>684</td><td>026</td><td>362E-01</td></tr>
<tr><td>LOC643623</td><td>NR_038906</td><td>ref|Homo sapiens uncharacterized LOC643623</td><td>hs|chr6:126039179-126039238</td><td>A_21_P0004682</td><td>694</td><td>398E-04</td><td>046</td><td>209E-01</td><td>588</td><td>006</td><td>865E-01</td></tr>
<tr><td>XLOC_l2_013131</td><td>TCONS_l2_00024933</td><td>linc|BROAD Institute lincRNA (XLOC_l2_01313</td><td>hs|chr6:137865379-137865118</td><td>A_21_P0013100</td><td>704</td><td>525E-04</td><td>-045</td><td>357E-01</td><td>625</td><td>000</td><td>100E+00</td></tr>
<tr><td>OSTCP1</td><td>NR_028496</td><td>ref|Homo sapiens oligosaccharyltransferase co</td><td>hs|chr6:159262350-159262291</td><td>A_24_P317450</td><td>875</td><td>316E-05</td><td>-053</td><td>706E-01</td><td>626</td><td>-021</td><td>375E-01</td></tr>
</table>

XLOC_l2_013233	TCONS_l2_00025055	linc|BROAD Institute lincRNA (XLOC_l2_013233	hs|chr6:170483849-170483790	A_21_P0013119	737	322E-04	046	719E-02	614	036	233E-01
ZNF204P	NR_002722	ref|Homo sapiens zinc finger protein 204, pseu	hs|chr6:27325667-27325608	A_33_P3221458	862	000E+00	072	477E-01	545	-010	742E-01
lnc-FARS2-2	lnc-FARS2-2:1	gb|Homo sapiens cDNA clone IMAGE:4523522	hs|chr6:5986231-5986290	A_19_P00801917	924	781E-04	-043	357E-01	681	-006	851E-01
ENST00000436672	ENST00000436672	Unknown	hs|chr6:75303976-75303917	A_21_P0013143	759	314E-05	053	867E-01	638	003	925E-01
lnc-AC009365.3.1-1	lnc-AC009365.3.1-1:2	linc|LNCipedia lincRNA (lnc-AC009365.3.1-1), li	hs|chr7:132344143-132344202	A_21_P0005576	627	654E-04	044	867E-01	580	-018	492E-01
lnc-LRRC61-2	lnc-LRRC61-2:2	linc|LNCipedia lincRNA (lnc-LRRC61-2), lincRNA	hs|chr7:150040405-150040464	A_21_P0005442	635	358E-05	-053	143E-01	595	006	849E-01
PAXIP1-AS2	NR_024477	ref|Homo sapiens PAXIP1 antisense RNA 2 (PA	hs|chr7:154738429-154738488	A_33_P3231076	828	878E-05	050	477E-01	658	-009	756E-01
lnc-EVX1-5	lnc-EVX1-5:3	linc|LNCipedia lincRNA (lnc-EVX1-5), lincRNA [l	hs|chr7:27226401-27226460	A_21_P0005552	642	626E-05	-051	357E-01	723	031	426E-01
ENST00000326391	ENST00000326391	gb|Homo sapiens polymerase (RNA) II (DNA di	hs|chr7:44056055-44054346	A_24_P332341	874	579E-06	-057	357E-01	650	-011	691E-01
lnc-RADIL-2	lnc-RADIL-2:1	linc|LNCipedia lincRNA (lnc-RADIL-2), lincRNA [	hs|chr7:4645505-4645446	A_21_P0005467	724	836E-04	043	477E-01	690	040	349E-01
ENST00000453012	ENST00000453012	gb|HY188447 RIKEN full-length enriched huma	hs|chr7:62672075-62672134	A_21_P0013202	672	407E-04	-046	100E+00	595	006	873E-01
MAGI2-AS3	NR_038344	ref|Homo sapiens MAGI2 antisense RNA 3 (MA	hs|chr7:79100338-79100397	A_19_P00316709	771	323E-04	046	209E-01	589	001	982E-01
RHPN1-AS1	NR_026785	ref|Homo sapiens RHPN1 antisense RNA 1 (hea	hs|chr8:144448997-144448938	A_23_P71503	649	752E-04	-043	867E-01	553	-012	639E-01
XLOC_l2_014182	TCONS_l2_00027727	linc|BROAD Institute lincRNA (XLOC_l2_014182	hs|chr8:39275181-39275240	A_21_P0013472	615	771E-04	-044	357E-01	601	028	246E-01
XLOC_l2_014504	TCONS_l2_00028188	linc|BROAD Institute lincRNA (XLOC_l2_014504	hs|chr8:47505181-47505122	A_21_P0013514	708	371E-09	-070	357E-01	577	-027	244E-01
ENST00000511103	ENST00000511103	ens|asparagine synthetase pseudogene 4 [Sou	hs|chr8:47610171-47610230	A_21_P0013475	816	710E-08	-066	706E-01	657	-004	944E-01
XLOC_l2_014217	TCONS_l2_00027777	linc|BROAD Institute lincRNA (XLOC_l2_014217	hs|chr8:58125772-58125831	A_21_P0013484	651	867E-04	043	100E+00	582	-006	864E-01
lnc-ASPH-6	lnc-ASPH-6:1	linc|LNCipedia lincRNA (lnc-ASPH-6), lincRNA [l	hs|chr8:61822136-61822077	A_21_P0005843	625	771E-04	-044	706E-01	630	029	205E-01
RRS1-AS1	NR_040434	ref|Homo sapiens RRS1 antisense RNA 1 (head	hs|chr8:67332026-67331967	A_21_P0000183	640	138E-04	049	706E-01	581	013	732E-01
PTTG3P	NR_002734	ref|Homo sapiens pituitary tumor-transformin	hs|chr8:67680130-67680071	A_23_P60016	616	000E+00	-077	143E-01	624	-003	929E-01
LOC286189	NR_038877	ref|Homo sapiens uncharacterized LOC286189	hs|chr8:69216152-69216093	A_21_P0000822	701	790E-04	044	477E-01	545	-008	769E-01
XLOC_l2_014098	TCONS_l2_00028458	linc|BROAD Institute lincRNA (XLOC_l2_014098	hs|chr8:7010257-7010316	A_21_P0013532	749	149E-04	049	477E-01	621	046	611E-02
lnc-ERI1-3	lnc-ERI1-3:1	linc|LNCipedia lincRNA (lnc-ERI1-3), lincRNA [ln	hs|chr8:8780210-8784509	A_21_P0005649	676	560E-04	044	209E-01	585	010	734E-01
ENST00000437852	ENST00000437852	Unknown	hs|chr9:116613134-116613075	A_21_P0013685	892	279E-05	-053	100E+00	659	009	834E-01
lnc-AGPAT2-2	lnc-AGPAT2-2:4	linc|LNCipedia lincRNA (lnc-AGPAT2-2), lincRNA	hs|chr9:139506605-139506546	A_21_P0006336	671	799E-04	044	209E-01	577	016	626E-01
ARRDC1-AS1	NR_122035	ref|Homo sapiens ARRDC1 antisense RNA 1 (A	hs|chr9:140510538-140510479	A_24_P120970	685	215E-04	-048	100E+00	625	-030	162E-01
SUGT1P1	NR_003667	ref|Homo sapiens SUGT1 pseudogene 1 (SUGT	hs|chr9:33508370-33504540	A_21_P0005985	805	461E-04	-045	100E+00	604	-037	223E-01
LINC01251	NR_109755	ref|Homo sapiens long intergenic non-protein	hs|chr9:33733032-33732973	A_33_P3302320	609	435E-06	-058	357E-01	590	005	863E-01
LINC00950	NR_024006	ref|Homo sapiens long intergenic non-protein	hs|chr9:35865315-35865374	A_32_P47157	634	278E-05	053	867E-01	566	005	891E-01
FAM27E3	NR_103833	ref|Homo sapiens family with sequence similar	hs|chr9:44998270-44998329	A_21_P0013919	686	276E-04	047	209E-01	639	-003	920E-01
AQP7P1	NR_002817	ref|Homo sapiens aquaporin 7 pseudogene 1 (	hs|chr9:67270274-67270215	A_33_P3245290	797	198E-06	059	719E-02	574	-020	456E-01
lnc-APBA1-2	lnc-APBA1-2:1	linc|LNCipedia lincRNA (lnc-APBA1-2), lincRNA	hs|chr9:72009284-72009225	A_21_P0006274	1028	133E-04	-049	867E-01	913	-034	133E-01
CTSLP8	NR_033405	ref|Homo sapiens cathepsin L pseudogene 8 (C	hs|chr9:90460801-90460860	A_21_P0000597	915	649E-05	-051	357E-01	681	-010	723E-01
FLJ43315	NR_033856	ref|Homo sapiens asparagine synthetase pseu	hs|chrUn_gl000211:91828-91887	A_33_P3363188	612	118E-04	-049	867E-01	579	-014	625E-01
XLOC_l2_015561	TCONS_l2_00030253	linc|BROAD Institute lincRNA (XLOC_l2_015561	hs|chrX:073231905-073231964	A_19_P00324814	811	666E-06	057	867E-01	712	012	654E-01
LOC286437	NR_039980	ref|Homo sapiens uncharacterized LOC286437	hs|chrX:103367139-103367080	A_33_P3807593	994	854E-07	061	867E-01	722	009	800E-01
XLOC_l2_015821	TCONS_l2_00030598	linc|BROAD Institute lincRNA (XLOC_l2_015821	hs|chrX:130877216-130877157	A_21_P0013831	611	184E-04	-048	100E+00	575	-020	425E-01
LINC00087	NR_024493	ref|Homo sapiens long intergenic non-protein	hs|chrX:134232498-134232439	A_33_P3413188	715	507E-05	-052	706E-01	658	019	395E-01
XLOC_l2_015632	TCONS_l2_00030371	linc|BROAD Institute lincRNA (XLOC_l2_015632	hs|chrX:136103899-136103958	A_21_P0013794	622	610E-04	044	209E-01	565	-001	988E-01
lnc-FAM156B-1	lnc-FAM156B-1:1	linc|LNCipedia lincRNA (lnc-FAM156B-1), lincRN	hs|chrX:52942198-52944043	A_21_P0006497	683	988E-04	043	477E-01	648	-090	212E-01
XLOC_l2_015738	TCONS_l2_00030495	linc|BROAD Institute lincRNA (XLOC_l2_015738	hs|chrX:53188147-53188088	A_21_P0013815	600	257E-08	-068	357E-01	570	010	702E-01
LOC100132741	NR_034004	ref|Homo sapiens uncharacterized LOC100132	hs|chrX:70922723-70922782	A_33_P3357097	774	971E-05	050	209E-01	593	-011	677E-01
lnc-CHIC1-2	lnc-CHIC1-2:1	linc|LNCipedia lincRNA (lnc-CHIC1-2), lincRNA [	hs|chrX:73072144-73072203	A_19_P00327297	685	956E-04	043	706E-01	607	009	761E-01
XLOC_l2_015762	TCONS_l2_00030539	linc|BROAD Institute lincRNA (XLOC_l2_015762	hs|chrX:74959136-74959077	A_21_P0013820	1205	626E-04	044	100E+00	793	035	196E-01
XLOC_l2_015894	TCONS_l2_00030903	linc|BROAD Institute lincRNA (XLOC_l2_015894	hs|chrY:16020161-16021154	A_21_P0013882	698	939E-04	043	100E+00	644	015	716E-01
TTTY5	NR_001541	ref|Homo sapiens testis-specific transcript, Y-li	hs|chrY:24443312-24443253	A_23_P34209	615	223E-05	053	719E-02	581	016	541E-01
IQCH-AS1	NR_040051	ref|Homo sapiens IQCH antisense RNA 1 (IQCH	unmapped	A_21_P0000893	756	137E-06	060	867E-01	592	012	640E-01
lnc-PCF11-1	lnc-PCF11-1:12	linc|LNCipedia lincRNA (lnc-PCF11-1), lincRNA [	unmapped	A_21_P0007248	742	394E-05	-052	357E-01	592	-003	926E-01
XLOC_l2_003050	TCONS_l2_00005758	linc|BROAD Institute lincRNA (XLOC_l2_003050	unmapped	A_21_P0011049	664	410E-05	-052	143E-01	565	008	793E-01
XLOC_l2_013931	TCONS_l2_00026769	linc|BROAD Institute lincRNA (XLOC_l2_013931	unmapped	A_21_P0013339	697	921E-07	-061	100E+00	600	020	514E-01
WFDC21P	NR_030732	ref|Homo sapiens WAP four-disulfide core dom	hs|chr17:58160989-58160930	A_21_P0009341	983	913E-03	034	191E-02	858	-089	135E-02
LINC00963	ENST00000444184	ens|long intergenic non-protein coding RNA 96	hs|chr9:132265982-132266041	A_19_P00321303	1019	891E-03	034	191E-02	794	100	355E-03
lnc-ZRANB1-2	lnc-ZRANB1-2:1	linc|LNCipedia lincRNA (lnc-ZRANB1-2), lincRNA	hs|chr10:126922521-126922580	A_21_P0006933	628	101E-03	-042	444E-02	749	079	303E-02
lnc-KATNAL2-4	lnc-KATNAL2-4:1	linc|LNCipedia lincRNA (lnc-KATNAL2-4), lincRN	hs|chr18:45194660-45206397	A_21_P0009476	703	787E-02	-023	444E-02	675	073	853E-03
ASMTL-AS1	NR_026710	ref|Homo sapiens ASMTL antisense RNA 1 (ASI	hs|chrY:1481698-1481757	A_23_P60793	725	661E-03	036	191E-02	701	053	382E-02
LINC01347	NR_029401	ref|Homo sapiens long intergenic non-protein	hs|chr1:243242350-243242291	A_21_P0010675	965	880E-02	023	209E-01	833	082	402E-03
LINC00999	NR_024497	ref|Homo sapiens long intergenic non-protein	hs|chr10:38717161-38717220	A_21_P0007057	790	856E-01	002	706E-01	736	054	236E-02
LINC00707	NR_038291	ref|Homo sapiens long intergenic non-protein	hs|chr10:6823381-6823440	A_21_P0006829	551	850E-01	-003	100E+00	697	-078	155E-02
NEAT1	NR_028272	ref|Homo sapiens nuclear paraspeckle assemb	hs|chr11:65192209-65192268	A_19_P00318409	1001	745E-01	004	867E-01	1041	137	599E-04
LOC100288798	NR_125381	ref|Homo sapiens uncharacterized LOC100288	hs|chr12:46781651-46781710	A_21_P0007544	808	128E-02	-033	867E-01	725	088	375E-03
C1RL-AS1	NR_026947	ref|Homo sapiens C1RL antisense RNA 1 (C1RL	hs|chr12:7264142-7264201	A_33_P3308585	691	655E-01	006	867E-01	741	101	143E-03
SMIM2-AS1	NR_104064	ref|Homo sapiens SMIM2 antisense RNA 1 (SM	hs|chr13:44722107-44722166	A_19_P00319971	691	949E-01	001	706E-01	694	066	163E-02

MEG3	NR_002766	ref|Homo sapiens maternally expressed 3 (non	hs|chr14:101327286-101327345	A_33_P3255434	1050	114E-01	021	477E-01	880	081	404E-03
DNM1P46	NR_003260	ref|Homo sapiens DNM1 pseudogene 46 (DNM	hs|chr15:100340038-100339979	A_33_P3276142	760	139E-02	033	719E-02	799	172	272E-04
WHAMMP1	NR_036650	ref|Homo sapiens WAS protein homolog assoc	hs|chr15:32822058-32821999	A_33_P3317553	1093	371E-03	038	867E-01	843	092	299E-03
LINC01583	NR_120367	ref|Homo sapiens long intergenic non-protein	hs|chr15:82387963-82388022	A_21_P0011334	531	304E-03	-039	706E-01	666	083	396E-03
UBE2Q2P1	NR_003661	ref|Homo sapiens ubiquitin-conjugating enzym	hs|chr15:85098273-85098214	A_24_P816384	926	596E-02	025	477E-01	754	084	390E-03
ENST00000492522	ENST00000492522	Unknown	hs|chr17:46782865-46782924	A_19_P00317046	541	904E-01	002	357E-01	726	149	376E-04
lnc-SIK1-2	lnc-SIK1-2:1	linc|LNCipedia lincRNA (lnc-SIK1-2), lincRNA [ln	hs|chr21:044808883-044808824	A_19_P00801752	812	680E-01	-006	477E-01	650	065	251E-02
LINC00313	NR_026863	ref|Homo sapiens long intergenic non-protein	hs|chr21:44891533-44891474	A_23_P381489	585	991E-01	000	100E+00	661	080	116E-02
lnc-AC069257.9.1-4	lnc-AC069257.9.1-4:16	linc|LNCipedia lincRNA (lnc-AC069257.9.1-4), li	hs|chr3:195437351-195437410	A_19_P00320047	841	656E-03	036	867E-01	877	064	120E-02
XLOC_l2_010831	TCONS_l2_00020758	linc|BROAD Institute lincRNA (XLOC_l2_01083	hs|chr4:129404613-129411223	A_21_P0012611	608	547E-01	-008	477E-01	629	075	576E-03
GUSBP1	NR_027028	ref|Homo sapiens glucuronidase, beta pseudog	hs|chr5:21491451-21491510	A_24_P84822	1025	100E-02	034	209E-01	930	108	158E-03
LOC401286	NR_117091	ref|Homo sapiens uncharacterized LOC401286	hs|chr6:168067630-168067571	A_33_P3409245	1152	257E-01	015	867E-01	1439	109	166E-02
PSORS1C3	NR_026816	ref|Homo sapiens psoriasis susceptibility 1 can	hs|chr6:31141572-31141513	A_33_P3297020	640	457E-02	027	209E-01	687	158	194E-04
LOC401320	NR_038889	ref|Homo sapiens uncharacterized LOC401320	hs|chr7:30590371-30590312	A_19_P00322938	925	335E-03	038	867E-01	704	063	137E-02
CCAT1	NR_108049	ref|Homo sapiens colon cancer associated tran	hs|chr8:128221174-128221115	A_21_P0005628	563	389E-01	-012	100E+00	703	-165	237E-04
PVT1	NR_003367	ref|Homo sapiens Pvt1 oncogene (non-protein	hs|chr8:129001472-129001531	A_21_P0005949	843	398E-01	-011	477E-01	872	061	268E-02
XLOC_l2_000791	TCONS_l2_00001041	linc|BROAD Institute lincRNA (XLOC_l2_00079	hs|chr1:016944434-016944375	A_21_P0010599	821	230E-01	-016	706E-01	846	066	262E-02
lnc-APITD1-1	lnc-APITD1-1:1	linc|LNCipedia lincRNA (lnc-APITD1-1), lincRNA	hs|chr1:10519717-10519776	A_21_P0001665	693	116E-02	-033	143E-01	601	-051	345E-02
lnc-PRMT6-2	lnc-PRMT6-2:2	linc|LNCipedia lincRNA (lnc-PRMT6-2), lincRNA	hs|chr1:106995051-106995110	A_21_P0001360	590	575E-02	-025	143E-01	746	058	415E-02
LOC729737	NR_039983	ref|Homo sapiens uncharacterized LOC729737	hs|chr1:140265-140206	A_21_P0013194	786	964E-01	-001	706E-01	683	052	350E-02
lnc-ZBTB17-2	lnc-ZBTB17-2:1	linc|LNCipedia lincRNA (lnc-ZBTB17-2), lincRNA	hs|chr1:16126629-16123409	A_21_P0001471	606	893E-02	-023	706E-01	666	059	209E-02
XLOC_l2_001206	TCONS_l2_00001638	linc|BROAD Institute lincRNA (XLOC_l2_00120	hs|chr1:178459243-178459184	A_21_P0010663	985	387E-01	012	867E-01	869	058	431E-02
LINC00083	XR_241155	ref|PREDICTED: Homo sapiens long intergenic	hs|chr1:178463608-178463549	A_21_P0014061	831	622E-01	007	706E-01	729	058	266E-02
C1orf220	NR_033186	ref|Homo sapiens chromosome 1 open reading	hs|chr1:178517965-178518024	A_33_P3259865	652	195E-01	-017	477E-01	662	-058	171E-02
FLJ23867	NR_026900	ref|Homo sapiens uncharacterized protein FLJ2	hs|chr1:180169795-180169854	A_33_P3220475	808	258E-01	015	100E+00	644	057	256E-02
LINC00862	ENST00000367356	ens|long intergenic non-protein coding RNA 86	hs|chr1:200343203-200343144	A_33_P3268129	588	109E-01	-021	209E-01	643	052	498E-02
LINC00467	NR_026761	ref|Homo sapiens long intergenic non-protein	hs|chr1:211565258-211565317	A_33_P3223097	966	183E-01	018	209E-01	813	065	151E-02
DUSP5P1	NR_002834	ref|Homo sapiens dual specificity phosphatase	hs|chr1:228788013-228788072	A_24_P367602	684	169E-03	-041	100E+00	638	069	146E-02
XLOC_l2_000123	TCONS_l2_00000181	linc|BROAD Institute lincRNA (XLOC_l2_00012	hs|chr1:27976175-27976234	A_21_P0010525	1429	451E-01	010	706E-01	1226	-085	474E-03
SNHG3	NR_036473	ref|Homo sapiens small nucleolar RNA host ge	hs|chr1:28836041-28836100	A_33_P3280945	1177	482E-01	-009	100E+00	906	-071	966E-03
SNHG12	NR_024127	ref|Homo sapiens small nucleolar RNA host ge	hs|chr1:28906090-28905179	A_23_P74581	1153	376E-01	012	706E-01	934	065	228E-02
MTMR9LP	NR_026850	ref|Homo sapiens myotubularin related protei	hs|chr1:32697338-32697279	A_24_P160413	970	775E-02	024	867E-01	700	081	863E-03
ENST00000437410	ENST00000437410	ens|heterogeneous nuclear ribonucleoprotein	hs|chr1:54441151-54441092	A_21_P0010617	758	168E-02	-032	100E+00	825	-057	234E-02
LOC100288069	NR_033908	ref|Homo sapiens uncharacterized LOC100288	hs|chr1:708428-708370	A_33_P3232624	963	150E-01	019	719E-02	799	078	532E-03
XLOC_l2_000727	TCONS_l2_00000966	linc|BROAD Institute lincRNA (XLOC_l2_00072	hs|chr1:741187-709610	A_21_P0010591	809	374E-01	012	209E-01	763	070	129E-02
LOC646626	NR_045484	ref|Homo sapiens uncharacterized LOC646626	hs|chr1:85743396-85743455	A_32_P703	813	226E-01	016	100E+00	617	067	101E-02
lnc-DR1-1	lnc-DR1-1:1	linc|LNCipedia lincRNA (lnc-DR1-1), lincRNA [ln	hs|chr1:93804415-93804474	A_19_P00330507	581	380E-01	-012	100E+00	660	050	367E-02
lnc-BNIP3-2	lnc-BNIP3-2:1	linc|LNCipedia lincRNA (lnc-BNIP3-2), lincRNA [	hs|chr10:133730253-133730194	A_21_P0007038	730	657E-01	-006	100E+00	796	061	165E-02
VIM-AS1	NR_108061	ref|Homo sapiens VIM antisense RNA 1 (VIM-A	hs|chr10:17271753-17271694	A_21_P0014785	691	279E-01	-015	706E-01	1066	180	378E-04
SVIL-AS1	NR_110923	ref|Homo sapiens SVIL antisense RNA 1 (SVIL-A	hs|chr10:29711188-29711247	A_33_P3257503	840	842E-03	-035	100E+00	696	-060	181E-02
GOLGA2P6	NR_120609	ref|Homo sapiens golgin A2 pseudogene 6 (GO	hs|chr10:30653375-30653316	A_33_P3326733	1149	164E-03	041	719E-02	832	066	963E-03
LINC00842	NR_033957	ref|Homo sapiens long intergenic non-protein	hs|chr10:47151178-47151119	A_23_P359214	842	953E-02	022	719E-02	1224	076	564E-03
LINC01468	NR_120641	ref|Homo sapiens long intergenic non-protein	hs|chr10:54211136-54211077	A_21_P0006762	547	138E-02	-032	143E-01	607	-064	270E-02
ENST00000440192	ENST00000440192	ens|syndecan binding protein (syntenin) pseud	hs|chr10:6335196-6335137	A_21_P0010857	858	907E-01	-002	100E+00	753	050	474E-02
PRKCQ-AS1	NR_036502	ref|Homo sapiens PRKCQ antisense RNA 1 (PR	hs|chr10:6626088-6626147	A_32_P232559	822	856E-01	-002	706E-01	620	-067	970E-03
NUDT9P1	NR_002779	ref|Homo sapiens nudix (nucleoside diphospha	hs|chr10:92911824-92911765	A_32_P40463	632	392E-01	012	209E-01	602	053	423E-02
lnc-ST3GAL1-1	lnc-ST3GAL1-1:1	linc|LNCipedia lincRNA (lnc-ST3GAL1-1), lincRN	hs|chr11:094646541-094646482	A_19_P00802390	623	191E-01	-018	100E+00	728	051	366E-02
CAND1.11	NR_103765	ref|Homo sapiens uncharacterized LOC100130	hs|chr11:10330136-10330195	A_33_P3216938	601	820E-03	-035	143E-01	644	093	126E-02
NCAM1-AS1	NR_034101	ref|Homo sapiens NCAM1 antisense RNA1 (NC	hs|chr11:113140324-113140265	A_21_P0000635	746	842E-01	003	706E-01	726	056	374E-02
lnc-LGALS12-2	lnc-LGALS12-2:3	linc|LNCipedia lincRNA (lnc-LGALS12-2), lincRN	hs|chr11:63263016-63263075	A_21_P0007217	765	446E-01	-010	100E+00	678	061	221E-02
MALAT1	NR_002819	ref|Homo sapiens metastasis associated lung a	hs|chr11:65268619-65268678	A_21_P0010982	1240	317E-02	029	477E-01	1205	052	274E-02
ENST00000625158	ENST00000625158	ens|Metastasis-associated lung adenocarcinon	hs|chr11:65269831-65269772	A_21_P0007464	881	500E-02	026	100E+00	1423	074	102E-02
lnc-LRP5-1	lnc-LRP5-1:2	linc|LNCipedia lincRNA (lnc-LRP5-1), lincRNA [ln	hs|chr11:68050282-68050341	A_21_P0007225	809	587E-01	007	706E-01	695	054	495E-02
SHANK2-AS3	NR_073536	ref|Homo sapiens SHANK2 antisense RNA 3 (SH	hs|chr11:70709606-70709665	A_33_P3397161	875	175E-01	018	477E-01	746	065	499E-02
lnc-DHCR7-1	lnc-DHCR7-1:1	linc|LNCipedia lincRNA (lnc-DHCR7-1), lincRNA	hs|chr11:71213359-71213300	A_21_P0007367	572	216E-01	-017	143E-01	672	-064	257E-02
lnc-P2RY2-2	lnc-P2RY2-2:1	linc|LNCipedia lincRNA (lnc-P2RY2-2), lincRNA [	hs|chr11:72916265-72916324	A_21_P0007235	688	570E-01	-008	477E-01	634	-056	245E-02
THRIL	NR_110375	ref|Homo sapiens TNF and HNRNPL related im	hs|chr12:125510560-125510501	A_21_P0007509	557	946E-02	-022	706E-01	670	-069	975E-03
LOC100130238	NR_024563	ref|Homo sapiens uncharacterized LOC100130	hs|chr12:132857351-132857410	A_19_P00803351	1028	627E-01	-007	477E-01	917	-052	275E-02
lnc-STAT6-1	lnc-STAT6-1:1	linc|LNCipedia lincRNA (lnc-STAT6-1), lincRNA [	hs|chr12:57478129-57478070	A_21_P0007712	613	170E-01	-018	867E-01	625	061	283E-02
KRT19P2	NR_036685	ref|Homo sapiens keratin 19 pseudogene 2 (KF	hs|chr12:95228736-95228795	A_33_P3846653	1087	612E-01	-007	867E-01	786	066	116E-02
XLOC_l2_003705	TCONS_l2_00006816	linc|BROAD Institute lincRNA (XLOC_l2_00370	hs|chr13:044561475-044561534	A_21_P0011141	766	902E-01	002	357E-01	779	066	380E-02
lnc-DLK1-6	lnc-DLK1-6:9	linc|LNCipedia lincRNA (lnc-DLK1-6), lincRNA [ln	hs|chr14:101402717-101402776	A_21_P0008390	676	210E-01	017	477E-01	740	107	136E-03

XLOC_l2_004315	TCONS_l2_00007925	linc|BROAD Institute lincRNA (XLOC_l2_00431	hs|chr14:103556285-103556344	A_21_P0011258	639	887E-01	-002	100E+00	659	068	471E-02
RPPH1	NR_002312	ref|Homo sapiens ribonuclease P RNA compon	hs|chr14:20811297-20811238	A_33_P3389394	1124	686E-02	024	867E-01	1457	065	164E-02
lnc-NEMF-1	lnc-NEMF-1:1	linc|LNCipedia lincRNA (lnc-NEMF-1), lincRNA [	hs|chr14:50328939-50328880	A_21_P0008435	586	774E-01	004	867E-01	646	081	175E-02
GOLGA8O	NM_001277308	ref|Homo sapiens golgin A8 family, member O	hs|chr15:23609518-23609577	A_23_P140614	731	221E-02	030	477E-01	740	053	347E-02
ENST00000568033	ENST00000568033	ref|PREDICTED: Homo sapiens uncharacterized	hs|chr15:28964938-28964879	A_21_P0011359	995	172E-03	041	477E-01	887	061	134E-02
XLOC_l2_004771	TCONS_l2_00008795	linc|BROAD Institute lincRNA (XLOC_l2_00477	hs|chr15:84946753-84946694	A_21_P0011337	693	186E-01	018	719E-02	677	091	278E-03
ENST00000572466	ENST00000572466	Unknown	hs|chr16:011318439-011318498	A_21_P0009093	906	591E-01	007	706E-01	823	079	171E-02
LOC101927334	NR_110913	ref|Homo sapiens uncharacterized LOC101927	hs|chr16:051069611-051069670	A_21_P0008936	666	940E-01	-001	100E+00	775	111	986E-03
LOC100190986	NR_024456	ref|Homo sapiens uncharacterized LOC100190	hs|chr16:21876568-21876509	A_24_P693321	1002	244E-02	030	477E-01	866	071	729E-03
MT1L	NR_001447	ref|Homo sapiens metallothionein 1L (gene/ps	hs|chr16:56652580-56652639	A_23_P427703	1273	553E-03	-036	100E+00	1299	141	103E-03
AFG3L1P	NR_003226	ref|Homo sapiens AFG3-like AAA ATPase 1, pse	hs|chr16:90062393-90062452	A_23_P355289	1078	739E-01	004	477E-01	882	-052	419E-02
lnc-C17orf97-7	lnc-C17orf97-7:1	linc|LNCipedia lincRNA (lnc-C17orf97-7), lincRN	hs|chr17:000056542-000056601	A_21_P0009373	636	150E-02	032	477E-01	685	052	413E-02
LRRC75A-AS1	NR_045024	ref|Homo sapiens LRRC75A antisense RNA 1 (L	hs|chr17:16344470-16344529	A_21_P0000530	901	745E-02	-024	477E-01	846	-052	277E-02
LINC01563	NR_110895	ref|Homo sapiens long intergenic non-protein	hs|chr17:20979250-20988112	A_21_P0009382	1210	478E-01	010	867E-01	1221	-080	976E-03
PYY2	NR_003064	ref|Homo sapiens peptide YY, 2 (pseudogene)	hs|chr17:26554908-26554967	A_24_P233078	1196	136E-02	033	477E-01	971	098	275E-02
KRT18P55	NR_028334	ref|Homo sapiens keratin 18 pseudogene 55 (K	hs|chr17:26603822-26603763	A_23_P373708	1208	434E-02	-027	100E+00	1227	-060	208E-02
SH3GL1P1	NR_033412	ref|Homo sapiens SH3-domain GRB2-like 1 pse	hs|chr17:30369620-30369679	A_33_P3545065	915	260E-01	-015	100E+00	762	068	961E-03
XLOC_l2_006101	TCONS_l2_00011289	linc|BROAD Institute lincRNA (XLOC_l2_00610	hs|chr17:43665719-43665660	A_21_P0011595	765	211E-03	040	477E-01	760	106	920E-03
ENST00000575202	ENST00000575202	Unknown	hs|chr17:46781374-46781433	A_21_P0009251	539	703E-02	-024	143E-01	653	133	250E-03
ENST00000478824	ENST00000478824	gb|Homo sapiens, clone IMAGE:3897156, mRN	hs|chr17:46784473-46784532	A_21_P0009168	688	971E-02	-022	143E-01	716	069	266E-02
MTVR2	NR_027025	ref|Homo sapiens mouse mammary tumor viru	hs|chr17:54962202-54962143	A_33_P3332302	605	461E-01	010	706E-01	627	077	162E-02
TBC1D3P1-DHX40P1	NR_002924	ref|Homo sapiens TBC1D3P1-DHX40P1 readthr	hs|chr17:58091718-58079727	A_21_P0014921	646	805E-01	003	477E-01	671	066	126E-02
LINC00482	NR_038080	ref|Homo sapiens long intergenic non-protein	hs|chr17:79276696-79276637	A_24_P323084	910	735E-02	024	477E-01	713	077	129E-02
LINC01540	NR_110429	ref|Homo sapiens long intergenic non-protein	hs|chr18:3478877-3478936	A_21_P0009442	787	969E-01	001	867E-01	603	-087	580E-03
LINC01478	NR_110792	ref|Homo sapiens long intergenic non-protein	hs|chr18:42098273-42098214	A_21_P0009629	827	611E-01	007	100E+00	890	111	357E-02
lnc-CCDC68-1	lnc-CCDC68-1:1	linc|LNCipedia lincRNA (lnc-CCDC68-1), lincRNA	hs|chr18:52558293-52558234	A_21_P0009558	735	131E-02	033	477E-01	756	-070	167E-02
GACAT2	NR_120598	ref|Homo sapiens gastric cancer associated tra	hs|chr18:8695924-8695865	A_21_P0014365	558	536E-02	-026	100E+00	995	191	276E-03
LOC729218	NR_109983	ref|Homo sapiens uncharacterized LOC729218	hs|chr19:197296-197237	A_33_P3238777	1203	591E-01	007	209E-01	853	069	932E-03
XLOC_l2_006548	TCONS_l2_00012221	linc|BROAD Institute lincRNA (XLOC_l2_00654	hs|chr19:304083-304142	A_21_P0011665	597	507E-01	009	209E-01	631	052	321E-02
UPK1A-AS1	NR_046420	ref|Homo sapiens UPK1A antisense RNA 1 (UPI	hs|chr19:36159480-36159421	A_23_P131036	569	754E-01	004	100E+00	607	052	335E-02
LINC01535	NR_110718	ref|Homo sapiens long intergenic non-protein	hs|chr19:37754265-37754324	A_21_P0011770	997	477E-01	010	867E-01	1354	139	126E-02
lnc-IGFL3-1	lnc-IGFL3-1:1	linc|LNCipedia lincRNA (lnc-IGFL3-1), lincRNA [	hs|chr19:46677222-46676750	A_21_P0009771	584	373E-02	-028	143E-01	837	-072	107E-02
lnc-EMP3-1	lnc-EMP3-1:1	linc|LNCipedia lincRNA (lnc-EMP3-1), lincRNA [	hs|chr19:48826387-48826446	A_21_P0009727	629	231E-01	-016	100E+00	647	077	107E-02
RAB11B-AS1	NR_038237	ref|Homo sapiens RAB11B antisense RNA 1 (RA	hs|chr19:8439374-8439315	A_21_P0000732	957	831E-03	035	209E-01	674	076	112E-02
lnc-NPAS2-1	lnc-NPAS2-1:2	linc|LNCipedia lincRNA (lnc-NPAS2-1), lincRNA	hs|chr2:101356411-101356470	A_21_P0002259	671	489E-02	-026	867E-01	752	062	181E-02
XLOC_l2_008221	TCONS_l2_00014844	linc|BROAD Institute lincRNA (XLOC_l2_00822	hs|chr2:114618877-114600898	A_21_P0011956	706	221E-01	016	706E-01	641	069	239E-02
XLOC_l2_008285	TCONS_l2_00014931	linc|BROAD Institute lincRNA (XLOC_l2_00828	hs|chr2:137087011-137086952	A_33_P3330683	1483	815E-01	-003	100E+00	1242	-095	308E-03
ENST00000423925	ENST00000423925	Unknown	hs|chr2:16946342-16948291	A_21_P0001837	869	754E-01	-004	706E-01	825	068	114E-02
LINC01126	NR_027251	ref|Homo sapiens long intergenic non-protein	hs|chr2:43455024-43455083	A_23_P348911	674	327E-01	-013	143E-01	732	064	467E-02
ANKRD36BP2	NR_015424	ref|Homo sapiens ankyrin repeat domain 36B	hs|chr2:89100699-89100758	A_24_P341089	1136	839E-03	035	209E-01	902	062	196E-02
LOC149950	NR_034152	ref|Homo sapiens uncharacterized LOC149950	hs|chr20:31196447-31196506	A_33_P3290235	756	555E-01	008	867E-01	820	115	846E-03
HMGB3P1	NR_002165	ref|Homo sapiens high mobility group box 3 ps	hs|chr20:33421521-33421462	A_23_P373119	1022	620E-03	-036	706E-01	916	-059	243E-02
FER1L4	NR_119376	ref|Homo sapiens fer-1-like family member 4,	hs|chr20:34146579-34146520	A_23_P80048	1034	275E-02	-029	477E-01	791	213	110E-04
LINC00494	NR_026958	ref|Homo sapiens long intergenic non-protein	hs|chr20:46999255-46999314	A_33_P3391275	723	586E-01	007	477E-01	818	071	212E-02
lnc-BIRC7-1	lnc-BIRC7-1:2	linc|LNCipedia lincRNA (lnc-BIRC7-1), lincRNA [	hs|chr20:61750484-61750543	A_21_P0009990	641	556E-01	-008	706E-01	669	068	310E-02
TMEM191A	NR_026815	ref|Homo sapiens transmembrane protein 191	hs|chr22:21058825-21058884	A_23_P166336	1042	487E-01	009	477E-01	806	093	608E-03
ENST00000510244	ENST00000510244	Unknown	hs|chr3:125627056-125626997	A_21_P0005657	791	312E-03	039	867E-01	600	056	257E-02
LINC00884	NR_033929	ref|Homo sapiens long intergenic non-protein	hs|chr3:194209094-194209153	A_33_P3390177	733	273E-01	-015	706E-01	894	-087	230E-02
lnc-AC069257.9.1-5	lnc-AC069257.9.1-5:2	linc|LNCipedia lincRNA (lnc-AC069257.9.1-5), li	hs|chr3:195377532-195377591	A_21_P0003090	587	149E-03	-041	706E-01	674	069	348E-02
NCBP2-AS2	NR_024388	ref|Homo sapiens NCBP2 antisense RNA 2 (hea	hs|chr3:196670329-196670388	A_23_P6708	1202	367E-03	-038	867E-01	855	-064	199E-02
XLOC_l2_010082	TCONS_l2_00019116	linc|BROAD Institute lincRNA (XLOC_l2_01008	hs|chr3:197366275-197366334	A_33_P3262028	681	544E-01	008	867E-01	730	055	475E-02
XLOC_l2_010139	TCONS_l2_00019209	linc|BROAD Institute lincRNA (XLOC_l2_01013	hs|chr3:27674957-27674898	A_21_P0012436	568	304E-02	-029	867E-01	780	081	433E-03
PDCD6IP	NM_001256192	ref|Homo sapiens programmed cell death 6 int	hs|chr3:33868584-33868643	A_21_P0000548	760	644E-02	025	719E-02	662	058	164E-02
ITPR1-AS1	NR_108075	ref|Homo sapiens ITPR1 antisense RNA 1 (head	hs|chr3:4532637-4532578	A_21_P0002837	739	530E-01	008	357E-01	752	060	441E-02
lnc-EPHA6-1	lnc-EPHA6-1:1	linc|LNCipedia lincRNA (lnc-EPHA6-1), lincRNA	hs|chr3:96336291-96336350	A_21_P0003242	789	791E-02	023	100E+00	870	107	581E-03
LOC100507487	NR_125882	ref|Homo sapiens uncharacterized LOC100507	hs|chr4:129440318-129440377	A_21_P0012739	707	651E-01	006	100E+00	623	066	130E-02
lnc-GYPA-1	lnc-GYPA-1:1	linc|LNCipedia lincRNA (lnc-GYPA-1), lincRNA [l	hs|chr4:145122682-145122623	A_21_P0003780	617	931E-01	-001	357E-01	626	065	143E-02
ANXA2P1	NR_001562	ref|Homo sapiens annexin A2 pseudogene 1 (A	hs|chr4:154228642-154228621	A_24_P204244	1108	866E-01	-002	100E+00	1067	069	139E-02
RPL21P44	NR_027153	ref|Homo sapiens ribosomal protein L21 pseud	hs|chr4:54853065-54853006	A_32_P94087	1381	205E-01	017	719E-02	1106	069	127E-02
EPB41L4A-AS1	NR_015370	ref|Homo sapiens EPB41L4A antisense RNA 1 (	hs|chr5:111497889-111497948	A_23_P58538	1211	932E-02	022	477E-01	914	061	231E-02
ENST00000523154	ENST00000523154	ref|PREDICTED: Homo sapiens uncharacterized	hs|chr5:139547328-139547387	A_19_P00322583	900	302E-03	039	100E+00	933	082	292E-02
LOC100268168	NR_026682	ref|Homo sapiens uncharacterized LOC100268	hs|chr5:172382521-172382462	A_33_P3391387	665	482E-01	-009	100E+00	729	077	105E-02

FAM153C	NR_038353	ref|Homo sapiens family with sequence similar	hs|chr5:175542646-175542705	A_32_P84369	729	281E-01	014	477E-01	632	120	126E-03
ENST00000511579	ENST00000511579	Unknown	hs|chr5:33520050-33519991	A_21_P0004121	1233	363E-02	028	209E-01	1260	-081	589E-03
lnc-AC091435.2-1	lnc-AC091435.2-1:1	linc|LNCipedia lincRNA (lnc-AC091435.2-1), linc	hs|chr5:38793086-38793145	A_21_P0004241	677	860E-02	-023	143E-01	783	073	193E-02
FLJ32255	NR_104643	ref|Homo sapiens uncharacterized LOC643977	hs|chr5:42991256-42991197	A_19_P00320314	828	550E-02	-026	100E+00	852	066	307E-02
NR2F1-AS1	NR_109818	ref|Homo sapiens NR2F1 antisense RNA 1 (NR2	hs|chr5:92878726-92878667	A_19_P00811286	600	127E-01	-020	706E-01	697	-053	258E-02
LOC100133050	NR_027503	ref|Homo sapiens glucuronidase, beta pseudog	hs|chr5:99717080-99717021	A_32_P39003	1030	797E-03	035	867E-01	934	104	172E-03
LOC441081	NR_073404	ref|Homo sapiens POM121 membrane glycopr	hs|chr6:026865219-026865278	A_24_P349207	835	128E-01	020	477E-01	734	056	392E-02
lnc-CCHCR1-1	lnc-CCHCR1-1:1	tc|Q6H1K9_HUMAN (Q6H1K9) Psoriasis suscep	hs|chr6:031145185-031145126	A_21_P0005161	584	509E-02	026	100E+00	660	142	447E-03
lnc-HACE1-3	lnc-HACE1-3:1	linc|LNCipedia lincRNA (lnc-HACE1-3), lincRNA	hs|chr6:104910862-104910803	A_21_P0005047	567	837E-01	-003	706E-01	603	067	114E-02
HLA-J	NR_024240	ref|Homo sapiens major histocompatibility cor	hs|chr6:29977388-29977447	A_24_P418044	1510	243E-01	016	477E-01	668	055	300E-02
lnc-CCHCR1-1	lnc-CCHCR1-1:4	linc|LNCipedia lincRNA (lnc-CCHCR1-1), lincRNA	hs|chr6:31139616-31139557	A_21_P0005160	534	469E-03	037	100E+00	601	055	360E-02
XLOC_l2_012953	TCONS_l2_00024711	linc|BROAD Institute lincRNA (XLOC_l2_01295	hs|chr6:31349758-31349699	A_21_P0013078	1551	243E-01	016	477E-01	703	059	167E-02
GGNBP1	NR_028361	ref|Homo sapiens gametogenetin binding prot	hs|chr6:33553482-33554489	A_24_P606538	577	461E-01	010	357E-01	622	-057	196E-02
LOC730101	NR_024403	ref|Homo sapiens uncharacterized LOC730101	hs|chr6:52531318-52531377	A_19_P00803360	825	347E-01	-013	357E-01	719	-093	514E-03
XLOC_l2_013031	TCONS_l2_00024809	linc|BROAD Institute lincRNA (XLOC_l2_01303	hs|chr6:69339910-69339851	A_21_P0013085	1217	789E-01	-004	706E-01	938	069	385E-02
lnc-FAM46A-2	lnc-FAM46A-2:3	linc|LNCipedia lincRNA (lnc-FAM46A-2), lincRN	hs|chr6:82553560-82553501	A_21_P0005036	784	872E-02	-023	706E-01	774	064	323E-02
XLOC_l2_013383	TCONS_l2_00025857	linc|BROAD Institute lincRNA (XLOC_l2_01338	hs|chr7:056438547-056438488	A_33_P3292130	1368	583E-02	025	477E-01	1366	-056	196E-02
KMT2E-AS1	NR_024586	ref|Homo sapiens KMT2E antisense RNA 1 (he	hs|chr7:104653832-104653773	A_21_P0005521	943	319E-01	013	706E-01	964	075	251E-02
lnc-CCDC71L-1	lnc-CCDC71L-1:3	linc|LNCipedia lincRNA (lnc-CCDC71L-1), lincRN	hs|chr7:106102571-106102512	A_19_P00321722	1062	195E-01	017	477E-01	1072	116	130E-02
LINC01000	NR_024368	ref|Homo sapiens long intergenic non-protein	hs|chr7:128300724-128300783	A_33_P3278881	794	990E-03	034	100E+00	780	070	127E-02
LOC155060	NR_036573	ref|Homo sapiens AI894139 pseudogene (LOC	hs|chr7:148994188-148994247	A_23_P254442	821	765E-01	-004	706E-01	655	060	398E-02
ATP6V0E2-AS1	NR_027040	ref|Homo sapiens ATP6V0E2 antisense RNA 1 (	hs|chr7:149564956-149564897	A_32_P146659	671	961E-03	-034	706E-01	602	-058	265E-02
LINC00689	ENST00000417032	ens|long intergenic non-protein coding RNA 68	hs|chr7:158799418-158799477	A_33_P3281018	734	194E-01	017	357E-01	803	089	251E-02
lnc-BRAT1-1	lnc-BRAT1-1:2	linc|LNCipedia lincRNA (lnc-BRAT1-1), lincRNA	hs|chr7:2514419-2514360	A_21_P0013410	740	596E-01	-007	357E-01	706	085	178E-02
LOC729218	NR_109983	ref|Homo sapiens uncharacterized LOC729218	hs|chr7:39807047-39807106	A_21_P0013267	823	318E-01	013	477E-01	767	075	200E-02
EGFR-AS1	NR_047551	ref|Homo sapiens EGFR antisense RNA 1 (EGFF	hs|chr7:55249544-55249485	A_32_P133395	546	283E-01	-014	867E-01	603	056	443E-02
lnc-GUSB-2	lnc-GUSB-2:1	linc|LNCipedia lincRNA (lnc-GUSB-2), lincRNA [l	hs|chr7:65564698-65564639	A_21_P0005605	678	503E-01	-009	100E+00	737	057	348E-02
ENST00000412091	ENST00000412091	Unknown	hs|chr7:65957296-65957237	A_21_P0013324	791	879E-01	002	209E-01	654	054	452E-02
RPL13AP17	NR_003680	ref|Homo sapiens ribosomal protein L13a pseu	hs|chr7:77988714-77988773	A_33_P3312802	963	198E-03	040	867E-01	778	072	417E-02
DMTF1	NR_024549	ref|Homo sapiens cyclin D binding myb-like tra	hs|chr7:86811421-86811480	A_33_P3381943	876	324E-02	028	209E-01	740	095	586E-03
ENST00000524369	ENST00000524369	Unknown	hs|chr8:102138349-102138290	A_21_P0005878	888	166E-01	-019	706E-01	787	056	241E-02
FAM66A	NR_026789	ref|Homo sapiens family with sequence similar	hs|chr8:12219964-12220023	A_33_P3316410	712	360E-03	038	100E+00	698	058	201E-02
lnc-FAM84B-6	lnc-FAM84B-6:3	linc|LNCipedia lincRNA (lnc-FAM84B-6), lincRN	hs|chr8:128153653-128153594	A_21_P0005904	544	567E-01	-008	706E-01	666	-065	122E-02
lnc-FAM84B-8	lnc-FAM84B-8:2	linc|LNCipedia lincRNA (lnc-FAM84B-8), lincRN	hs|chr8:128186497-128186438	A_21_P0005905	515	361E-01	-012	477E-01	605	-089	600E-03
lnc-ASAP1-1	lnc-ASAP1-1:1	linc|LNCipedia lincRNA (lnc-ASAP1-1), lincRNA	hs|chr8:131455734-131440038	A_21_P0005976	805	391E-01	012	867E-01	831	058	208E-02
KIAA1875	NR_024207	ref|Homo sapiens KIAA1875 (KIAA1875), long r	hs|chr8:145172632-145172680	A_33_P3290955	1164	342E-02	028	867E-01	1349	097	378E-02
LOC101927815	NR_125426	ref|Homo sapiens uncharacterized LOC101927	hs|chr8:2554222-2554163	A_21_P0005621	755	228E-01	-016	357E-01	712	059	152E-02
lnc-ZMAT4-3	lnc-ZMAT4-3:4	linc|LNCipedia lincRNA (lnc-ZMAT4-3), lincRNA	hs|chr8:40156308-40156249	A_21_P0005824	542	778E-01	004	706E-01	683	063	350E-02
lnc-RAB2A-1	lnc-RAB2A-1:1	linc|LNCipedia lincRNA (lnc-RAB2A-1), lincRNA	hs|chr8:60987226-60987285	A_21_P0005703	728	677E-01	-006	143E-01	888	117	527E-03
DEFB109P1B	NR_003668	ref|Homo sapiens defensin, beta 109, pseudog	hs|chr8:7177414-7177473	A_33_P3240163	550	494E-01	009	706E-01	636	072	449E-02
LOC392196	NR_003275	ref|Homo sapiens ubiquitin specific peptidase	hs|chr8:7200733-7200792	A_33_P3338491	652	357E-01	-012	706E-01	731	062	265E-02
lnc-TMEM64-1	lnc-TMEM64-1:5	linc|LNCipedia lincRNA (lnc-TMEM64-1), lincRN	hs|chr8:91566582-91546799	A_21_P0005869	723	765E-01	-004	100E+00	953	-128	149E-03
lnc-DCAF10-2	lnc-DCAF10-2:1	linc|LNCipedia lincRNA (lnc-DCAF10-2), lincRN	hs|chr9:037905553-037905612	A_21_P0006347	749	834E-01	-003	706E-01	627	056	221E-02
LOC100129034	NR_027406	ref|Homo sapiens uncharacterized LOC100129	hs|chr9:127120692-127120751	A_32_P91042	754	122E-03	042	867E-01	636	063	174E-02
SLC25A51	NR_024872	ref|Homo sapiens solute carrier family 25, mer	hs|chr9:37877631-37877572	A_33_P3255499	1043	201E-02	031	706E-01	880	062	339E-02
LOC403323	NR_122077	ref|Homo sapiens uncharacterized LOC403323	hs|chr9:46687913-46687972	A_33_P3346327	813	604E-02	025	477E-01	999	-185	353E-04
LOC642236	NR_033907	ref|Homo sapiens FSHD region gene 1 pseudog	hs|chr9:68727550-68727609	A_33_P3310366	1018	155E-01	019	477E-01	1032	084	701E-03
XLOC_l2_015464	TCONS_l2_00030158	linc|BROAD Institute lincRNA (XLOC_l2_01546	hs|chrX:018884701-018884760	A_33_P3354296	888	158E-02	-032	143E-01	680	-076	281E-02
XLOC_l2_015760	TCONS_l2_00030535	linc|BROAD Institute lincRNA (XLOC_l2_01576	hs|chrX:073466010-073465951	A_21_P0013819	682	544E-02	026	209E-01	637	079	112E-02
lnc-PLS3-2	lnc-PLS3-2:1	linc|LNCipedia lincRNA (lnc-PLS3-2), lincRNA [l	hs|chrX:114961240-114961299	A_21_P0006570	671	562E-01	-008	477E-01	765	089	346E-03
ENST00000421897	ENST00000421897	Unknown	hs|chrX:27882376-27882435	A_21_P0013756	1017	198E-01	-017	100E+00	694	052	397E-02
XLOC_l2_015478	TCONS_l2_00030171	linc|BROAD Institute lincRNA (XLOC_l2_01547	hs|chrX:30649021-30649080	A_21_P0013757	1760	583E-01	-007	100E+00	1519	-124	866E-03
FTX	NR_028379	ref|Homo sapiens FTX transcript, XIST regulato	hs|chrX:73501599-73501540	A_21_P0013866	755	179E-02	031	477E-01	791	065	153E-02
UBE2DNL	NR_024062	ref|Homo sapiens ubiquitin-conjugating enzym	hs|chrX:84189670-84189729	A_23_P367071	653	823E-01	003	100E+00	844	-120	670E-03
XLOC_l2_015938	TCONS_l2_00030929	linc|BROAD Institute lincRNA (XLOC_l2_01593	hs|chrY:10036168-10035807	A_21_P0013894	1200	512E-01	009	867E-01	1257	206	429E-02
ENST00000421819	ENST00000421819	ens|ubiquitin-conjugating enzyme E2Q family	hs|chrY:26291082-26292234	A_21_P0013885	977	373E-02	028	477E-01	819	054	350E-02
lnc-APLN-1	lnc-APLN-1:1	linc|LNCipedia lincRNA (lnc-APLN-1), lincRNA [l	unmapped	A_21_P0006549	801	484E-01	009	477E-01	743	068	128E-02
LOC104968399	NR_130145	ref|Homo sapiens (LOC104968399), transcript	hs|chr18:3255511-3255452	A_21_P0014363	550	965E-05	-050	444E-02	566	008	759E-01
lnc-LRRC58-1	lnc-LRRC58-1:2	linc|LNCipedia lincRNA (lnc-LRRC58-1), lincRNA	hs|chr3:120084906-120084847	A_21_P0003296	582	296E-05	-053	444E-02	633	015	634E-01
lnc-CCDC37-3	lnc-CCDC37-3:1	linc|LNCipedia lincRNA (lnc-CCDC37-3), lincRNA	hs|chr3:125965876-125965935	A_21_P0003026	575	828E-04	043	191E-02	567	-018	544E-01
LOC389641	NR_033928	ref|Homo sapiens uncharacterized LOC389641	hs|chr8:23088374-23088433	A_33_P3442605	568	215E-04	-048	444E-02	555	011	740E-01
lnc-RALGDS-2	lnc-RALGDS-2:1	linc|LNCipedia lincRNA (lnc-RALGDS-2), lincRNA	hs|chr9:135903577-135903518	A_21_P0006331	571	644E-04	-044	106E-02	556	011	674E-01

lnc-ARHGAP6-1	lnc-ARHGAP6-1:3	linc|LNCipedia lincRNA (lnc-ARHGAP6-1), lincRI	hs|chrX:11125061-11074143	A_21_P0006526	553	878E-05	-050	444E-02	575	-002	960E-01
TTTY6	NR_001527	ref|Homo sapiens testis-specific transcript, Y-li	hs|chrY:24292823-24292882	A_23_P171409	560	425E-04	045	191E-02	575	010	726E-01
lnc-RRP1B-2	lnc-RRP1B-2:1	linc|LNCipedia lincRNA (lnc-RRP1B-2), lincRNA	hs|chr21:44887367-44887426	A_21_P0010260	612	115E-02	-033	444E-02	639	044	486E-02
PCAT6	NR_046326	ref|Homo sapiens prostate cancer associated t	hs|chr1:202780319-202780378	A_21_P0001711	590	208E-01	-017	106E-02	601	042	131E-01
HOTAIR	NR_047518	ref|Homo sapiens HOX transcript antisense RN	hs|chr12:54356679-54356620	A_19_P00330814	576	973E-01	000	106E-02	581	-003	914E-01
LINC01021	NR_038848	ref|Homo sapiens long intergenic non-protein	hs|chr5:27494366-27494425	A_19_P00321053	549	547E-01	008	191E-02	595	019	436E-01
HCG18	NR_024052	ref|Homo sapiens HLA complex group 18 (non-	hs|chr6:30262563-30262504	A_19_P00320396	740	309E-01	-014	444E-02	594	-004	931E-01
KHDC1	NR_027005	ref|Homo sapiens KH homology domain contai	hs|chr6:73984736-73984677	A_19_P00325810	677	181E-02	-031	444E-02	613	-009	760E-01
LINC01278	NR_015353	ref|Homo sapiens long intergenic non-protein	hs|chrX:62780783-62780724	A_19_P00316325	779	179E-02	031	387E-03	686	-011	737E-01
LINC01225	ENST00000563550	ens|long intergenic non-protein coding RNA 12	hs|chr1:031974776-031974835	A_33_P3339202	704	923E-01	001	444E-02	722	064	103E-01
lnc-MTOR-1	lnc-MTOR-1:1	linc|LNCipedia lincRNA (lnc-MTOR-1), lincRNA [	hs|chr1:11371863-11371804	A_21_P0001466	542	204E-02	-031	444E-02	571	-007	827E-01
LOC101929181	NR_104624	ref|Homo sapiens uncharacterized LOC101929	hs|chr1:11672885-11672944	A_21_P0000949	699	228E-01	-016	444E-02	727	038	203E-01
lnc-WDR3-2	lnc-WDR3-2:1	linc|LNCipedia lincRNA (lnc-WDR3-2), lincRNA [	hs|chr1:118749565-118749624	A_21_P0001367	549	101E-01	-022	195E-03	562	010	753E-01
ENST00000457719	ENST00000457719	Unknown	hs|chr1:119803136-119803077	A_21_P0001171	626	298E-02	-029	444E-02	649	042	106E-01
lnc-VPS13D-1	lnc-VPS13D-1:1	linc|LNCipedia lincRNA (lnc-VPS13D-1), lincRNA	hs|chr1:12588513-12588572	A_21_P0001263	561	714E-01	-005	444E-02	589	008	779E-01
lnc-RP3-377D14.1.1-4	lnc-RP3-377D14.1.1-4:1	linc|LNCipedia lincRNA (lnc-RP3-377D14.1.1-4)	hs|chr1:143189463-143189522	A_21_P0001011	689	737E-01	-005	191E-02	688	016	485E-01
lnc-TTC24-1	lnc-TTC24-1:1	linc|LNCipedia lincRNA (lnc-TTC24-1), lincRNA [	hs|chr1:156483049-156483108	A_21_P0001376	514	841E-01	-003	444E-02	551	006	848E-01
lnc-CD1B-3	lnc-CD1B-3:1	linc|LNCipedia lincRNA (lnc-CD1B-3), lincRNA [l	hs|chr1:158097162-158096916	A_21_P0001586	535	115E-02	-033	106E-02	575	-008	792E-01
ENST00000448643	ENST00000448643	Unknown	hs|chr1:166357026-166356967	A_21_P0001186	575	603E-02	025	387E-03	576	009	732E-01
RASAL2-AS1	NR_027982	ref|Homo sapiens RASAL2 antisense RNA 1 (RA	hs|chr1:178061262-178061203	A_21_P0000550	542	153E-02	-032	444E-02	556	-016	514E-01
lnc-NUAK2-1	lnc-NUAK2-1:1	linc|LNCipedia lincRNA (lnc-NUAK2-1), lincRNA	hs|chr1:205251660-205251601	A_21_P0001613	575	529E-01	-009	444E-02	619	-007	796E-01
LOC148696	NR_026817	ref|Homo sapiens uncharacterized LOC148696	hs|chr1:207994997-207995056	A_19_P00318375	549	504E-02	-026	444E-02	578	-005	869E-01
lnc-CAMK1G-5	lnc-CAMK1G-5:5	linc|LNCipedia lincRNA (lnc-CAMK1G-5), lincRN	hs|chr1:208907380-208909509	A_21_P0001413	518	941E-01	-001	387E-03	555	-008	776E-01
ENST00000440104	ENST00000440104	Unknown	hs|chr1:221684171-221684230	A_21_P0010739	544	969E-01	001	106E-02	563	002	941E-01
lnc-IRF2BP2-3	lnc-IRF2BP2-3:1	linc|LNCipedia lincRNA (lnc-IRF2BP2-3), lincRN/	hs|chr1:234852511-234852452	A_21_P0001761	559	569E-01	-008	444E-02	583	-001	985E-01
ENST00000442712	ENST00000442712	ref|PREDICTED: Homo sapiens uncharacterized	hs|chr1:246855064-246855123	A_21_P0001447	609	344E-01	013	387E-03	572	013	625E-01
lnc-TAF12-3	lnc-TAF12-3:1	linc|LNCipedia lincRNA (lnc-TAF12-3), lincRNA [	hs|chr1:29197120-29197061	A_21_P0001112	629	348E-01	013	106E-02	595	010	708E-01
lnc-PTPRU-2	lnc-PTPRU-2:1	linc|LNCipedia lincRNA (lnc-PTPRU-2), lincRNA	hs|chr1:30159192-30161259	A_21_P0001277	543	355E-01	012	191E-02	573	005	882E-01
lnc-HPCA-1	lnc-HPCA-1:1	linc|LNCipedia lincRNA (lnc-HPCA-1), lincRNA [l	hs|chr1:33399800-33399859	A_21_P0001671	525	207E-01	-017	444E-02	569	008	780E-01
lnc-ELAVL4-2	lnc-ELAVL4-2:1	linc|LNCipedia lincRNA (lnc-ELAVL4-2), lincRNA	hs|chr1:50772989-50773048	A_21_P0001294	527	617E-01	007	191E-02	549	-013	722E-01
LOC100505887	ENST00000414210	Unknown	hs|chr1:6784760-6784819	A_21_P0001664	603	603E-01	007	444E-02	606	018	531E-01
lnc-SLC44A5-4	lnc-SLC44A5-4:1	linc|LNCipedia lincRNA (lnc-SLC44A5-4), lincRN	hs|chr1:76186074-76176294	A_21_P0001142	750	378E-01	-012	444E-02	770	073	131E-01
lnc-PTGFR-1	lnc-PTGFR-1:1	linc|LNCipedia lincRNA (lnc-PTGFR-1), lincRNA	hs|chr1:78689353-78691008	A_21_P0001688	593	535E-02	026	191E-02	571	-005	888E-01
LINC01128	NR_047519	ref|Homo sapiens long intergenic non-protein	hs|chr1:789523-789582	A_24_P750305	667	271E-02	-029	444E-02	565	-010	748E-01
XLOC_l2_000961	TCONS_l2_00001297	linc|BROAD Institute lincRNA (XLOC_l2_00096	hs|chr1:79561171-79561112	A_21_P0010627	530	728E-01	005	387E-03	559	001	990E-01
ENST00000426794	ENST00000426794	Unknown	hs|chr1:86066311-86066370	A_21_P0000992	534	347E-01	-013	444E-02	562	-008	756E-01
lnc-ABCD3-2	lnc-ABCD3-2:4	linc|LNCipedia lincRNA (lnc-ABCD3-2), lincRNA	hs|chr1:94800322-94808287	A_21_P0001342	560	207E-01	017	191E-02	577	034	286E-01
lnc-C1orf200-3	lnc-C1orf200-3:1	linc|LNCipedia lincRNA (lnc-C1orf200-3), lincRN	hs|chr1:9509353-9509294	A_21_P0001465	532	770E-01	-004	444E-02	567	-002	958E-01
lnc-RWDD3-2	lnc-RWDD3-2:1	linc|LNCipedia lincRNA (lnc-RWDD3-2), lincRNA	hs|chr1:95787107-95787166	A_21_P0001348	533	200E-01	-017	444E-02	554	010	730E-01
lnc-FRRS1-1	lnc-FRRS1-1:1	linc|LNCipedia lincRNA (lnc-FRRS1-1), lincRNA [	hs|chr1:99937982-99937923	A_21_P0001159	536	384E-01	012	191E-02	564	-001	988E-01
lnc-PITRM1-7	lnc-PITRM1-7:8	linc|LNCipedia lincRNA (lnc-PITRM1-7), lincRNA	hs|chr10:002489269-002489210	A_21_P0006946	535	241E-01	-016	444E-02	565	016	494E-01
XLOC_l2_001669	TCONS_l2_00003150	linc|BROAD Institute lincRNA (XLOC_l2_00166	hs|chr10:089579300-089579359	A_21_P0010838	635	514E-02	-026	444E-02	670	045	229E-01
lnc-ABLIM1-1	lnc-ABLIM1-1:1	linc|LNCipedia lincRNA (lnc-ABLIM1-1), lincRNA	hs|chr10:116581048-116579688	A_21_P0007115	551	773E-01	004	444E-02	576	-010	761E-01
lnc-EBF3-4	lnc-EBF3-4:1	linc|LNCipedia lincRNA (lnc-EBF3-4), lincRNA [ln	hs|chr10:131170533-131170474	A_21_P0007034	652	299E-01	-014	444E-02	662	015	570E-01
lnc-NMT2-1	lnc-NMT2-1:1	linc|LNCipedia lincRNA (lnc-NMT2-1), lincRNA [	hs|chr10:15227059-15227000	A_21_P0006973	632	126E-02	033	191E-02	583	-013	622E-01
LOC102724039	ENST00000434386	Unknown	hs|chr10:16330985-16331044	A_21_P0006656	518	337E-01	-013	444E-02	560	010	686E-01
LINC00700	NR_040253	ref|Homo sapiens long intergenic non-protein	hs|chr10:2047738-2047679	A_33_P3740427	568	585E-02	025	191E-02	582	013	659E-01
LINC01552	NR_103721	ref|Homo sapiens long intergenic non-protein	hs|chr10:23492804-23492745	A_33_P3284838	647	211E-02	031	191E-02	576	017	483E-01
LINC01516	NR_120649	ref|Homo sapiens long intergenic non-protein	hs|chr10:25402603-25405587	A_21_P0006660	526	725E-01	-005	106E-02	558	-014	634E-01
AKR1C8P	NR_027916	ref|Homo sapiens aldo-keto reductase family 1	hs|chr10:5203879-5203820	A_33_P3329028	537	400E-01	011	191E-02	558	030	266E-01
lnc-MBL2-2	lnc-MBL2-2:2	linc|LNCipedia lincRNA (lnc-MBL2-2), lincRNA [l	hs|chr10:54731822-54731763	A_21_P0006997	534	307E-01	-014	444E-02	542	-004	921E-01
lnc-JMJD1C-1	lnc-JMJD1C-1:1	linc|LNCipedia lincRNA (lnc-JMJD1C-1), lincRNA	hs|chr10:64884545-64884486	A_21_P0006766	550	859E-02	-023	444E-02	590	002	947E-01
lnc-AL137145.1-3	lnc-AL137145.1-3:1	linc|LNCipedia lincRNA (lnc-AL137145.1-3), linc	hs|chr10:6663852-6663911	A_21_P0006827	536	207E-01	-017	444E-02	579	023	561E-01
lnc-ITIH2-4	lnc-ITIH2-4:1	linc|LNCipedia lincRNA (lnc-ITIH2-4), lincRNA [l	hs|chr10:7186733-7186792	A_21_P0006832	551	322E-02	028	191E-02	568	015	582E-01
GLUD1P3	NR_048575	ref|Homo sapiens glutamate dehydrogenase 1	hs|chr10:75491317-75491376	A_33_P3295148	1464	762E-03	035	191E-02	1322	-057	638E-02
lnc-RPS24-2	lnc-RPS24-2:1	linc|LNCipedia lincRNA (lnc-RPS24-2), lincRNA [	hs|chr10:79973772-79973831	A_21_P0007067	673	873E-01	002	106E-02	635	004	914E-01
lnc-GRID1-1	lnc-GRID1-1:1	linc|LNCipedia lincRNA (lnc-GRID1-1), lincRNA [	hs|chr10:87339854-87339795	A_21_P0007007	552	412E-01	011	191E-02	575	-013	626E-01
ENST00000414903	ENST00000414903	Unknown	hs|chr10:92262663-92262722	A_21_P0006694	572	249E-02	030	326E-02	556	-017	480E-01
lnc-RDX-1	lnc-RDX-1:1	linc|LNCipedia lincRNA (lnc-RDX-1), lincRNA [ln	hs|chr11:109693672-109651727	A_21_P0007396	544	219E-02	030	191E-02	558	022	403E-01
LOC100652768	NR_045215	ref|Homo sapiens uncharacterized LOC100652	hs|chr11:117068046-117067987	A_21_P0014887	606	864E-01	002	191E-02	602	014	705E-01
LINC01001	NR_028326	ref|Homo sapiens long intergenic non-protein	hs|chr11:128915-128856	A_33_P3352687	1393	174E-01	018	191E-02	1371	-031	325E-01
lnc-INSC-2	lnc-INSC-2:1	linc|LNCipedia lincRNA (lnc-INSC-2), lincRNA [ln	hs|chr11:15682066-15682125	A_21_P0007179	529	246E-01	-016	444E-02	568	012	633E-01

WT1-AS	NR_120548	ref|Homo sapiens WT1 antisense RNA (WT1-AS	hs|chr11:32475629-32475688	A_21_P0010968	545	232E-01	016	444E-02	570	-003	945E-01
CSTF3-AS1	NR_034027	ref|Homo sapiens CSTF3 antisense RNA 1 (head	hs|chr11:33211781-33211840	A_33_P3311621	562	286E-03	-039	444E-02	571	000	998E-01
lnc-CTD-2210P24.4.1-3	lnc-CTD-2210P24.4.1-3:1	linc|LNCipedia lincRNA (lnc-CTD-2210P24.4.1-3	hs|chr11:45602155-45602214	A_21_P0007206	580	957E-01	-001	106E-02	565	011	831E-01
LOC143666	NR_026967	ref|Homo sapiens uncharacterized LOC143666	hs|chr11:574411-574352	A_33_P3353864	641	656E-01	-006	444E-02	587	024	328E-01
lnc-CCND1-1	lnc-CCND1-1:1	linc|LNCipedia lincRNA (lnc-CCND1-1), lincRNA	hs|chr11:69680412-69681838	A_21_P0007230	586	590E-01	-007	444E-02	598	016	552E-01
LOC100128494	NR_104178	ref|Homo sapiens uncharacterized LOC100128	hs|chr11:71729219-71729278	A_33_P3348519	655	113E-01	-021	444E-02	653	030	237E-01
LOC101929084	NR_110103	ref|Homo sapiens uncharacterized LOC101929	hs|chr12:103953427-103953486	A_21_P0007490	517	910E-01	002	191E-02	570	010	788E-01
lnc-MED13L-1	lnc-MED13L-1:1	linc|LNCipedia lincRNA (lnc-MED13L-1), lincRN/	hs|chr12:116796795-116796736	A_21_P0007769	600	142E-02	032	191E-02	574	-006	857E-01
lnc-DHX37-8	lnc-DHX37-8:1	linc|LNCipedia lincRNA (lnc-DHX37-8), lincRNA	hs|chr12:126929825-126929766	A_21_P0007782	519	220E-01	-016	444E-02	542	009	764E-01
LINC00938	NR_028408	ref|Homo sapiens long intergenic non-protein (	hs|chr12:46120164-46120105	A_24_P661641	715	207E-01	-017	444E-02	565	-025	581E-01
lnc-TMEM106C-1	lnc-TMEM106C-1:2	linc|LNCipedia lincRNA (lnc-TMEM106C-1), linc	hs|chr12:48405182-48405241	A_21_P0007550	580	234E-01	-016	444E-02	617	-007	824E-01
LINC00592	NR_027358	ref|Homo sapiens long intergenic non-protein (	hs|chr12:52617464-52617523	A_21_P0007476	640	154E-01	-019	191E-02	664	-007	844E-01
lnc-CTDSP2-3	lnc-CTDSP2-3:1	linc|LNCipedia lincRNA (lnc-CTDSP2-3), lincRNA	hs|chr12:58475072-58475013	A_21_P0007714	529	406E-02	027	191E-02	567	-014	555E-01
lnc-FOXJ2-1	lnc-FOXJ2-1:1	linc|LNCipedia lincRNA (lnc-FOXJ2-1), lincRNA [	hs|chr12:8179212-8179271	A_21_P0007520	547	196E-03	040	387E-03	573	-006	849E-01
LINC00937	ENST00000544461	ens|long intergenic non-protein coding RNA 93	hs|chr12:8543510-8543451	A_33_P3364651	639	102E-02	034	191E-02	587	-007	793E-01
lnc-DAOA-6	lnc-DAOA-6:1	linc|LNCipedia lincRNA (lnc-DAOA-6), lincRNA [	hs|chr13:104975832-105020474	A_21_P0008111	578	210E-01	017	191E-02	608	-043	308E-01
LINC00460	NR_034119	ref|Homo sapiens long intergenic non-protein (	hs|chr13:107029573-107029632	A_21_P0007962	582	127E-01	-020	191E-02	568	014	614E-01
LINC00399	NR_126361	ref|Homo sapiens long intergenic non-protein (	hs|chr13:110053148-110053739	A_21_P0007965	524	281E-01	-014	195E-03	568	011	720E-01
lnc-TUBGCP3-7	lnc-TUBGCP3-7:1	linc|LNCipedia lincRNA (lnc-TUBGCP3-7), lincRN	hs|chr13:112834954-112834895	A_21_P0008218	513	270E-02	-029	444E-02	575	-069	232E-01
lnc-PCID2-1	lnc-PCID2-1:1	ref|PREDICTED: Homo sapiens uncharacterized	hs|chr13:113805066-113805007	A_21_P0008225	586	813E-03	035	387E-03	576	027	223E-01
LOC101928697	NR_126054	ref|Homo sapiens uncharacterized LOC101928	hs|chr13:19761342-19761401	A_33_P3269817	516	204E-01	-017	444E-02	554	-003	925E-01
LINC00540	NR_103810	ref|Homo sapiens long intergenic non-protein (	hs|chr13:22850538-22850597	A_21_P0007907	669	415E-02	-027	444E-02	565	011	664E-01
lnc-SGCG-1	lnc-SGCG-1:1	linc|LNCipedia lincRNA (lnc-SGCG-1), lincRNA [l	hs|chr13:23744177-23744735	A_21_P0007924	662	300E-01	-014	444E-02	651	024	458E-01
SACS-AS1	NR_103450	ref|Homo sapiens SACS antisense RNA 1 (SACS	hs|chr13:24002926-24002985	A_21_P0014499	546	440E-01	-010	444E-02	561	004	901E-01
LINC00463	XR_245411	ref|PREDICTED: Homo sapiens uncharacterized	hs|chr13:25749471-25749412	A_21_P0011222	531	788E-01	004	191E-02	569	019	448E-01
USP12-AS2	NR_046548	ref|Homo sapiens USP12 antisense RNA 2 (hea	hs|chr13:27757072-27757131	A_21_P0007927	660	205E-02	-031	444E-02	637	-002	967E-01
lnc-EPSTI1-3	lnc-EPSTI1-3:1	linc|LNCipedia lincRNA (lnc-EPSTI1-3), lincRNA	hs|chr13:43312703-43312644	A_21_P0008147	582	773E-02	024	598E-04	586	016	687E-01
ENST00000426509	ENST00000426509	ens|long intergenic non-protein coding RNA 40	hs|chr13:45275093-45275152	A_21_P0007934	637	475E-01	010	191E-02	613	-015	664E-01
LINC01198	ENST00000595532	ens|long intergenic non-protein coding RNA 11	hs|chr13:47032960-47032901	A_33_P3300985	522	116E-01	-021	444E-02	561	-002	949E-01
lnc-DIAPH3-6	lnc-DIAPH3-6:1	linc|LNCipedia lincRNA (lnc-DIAPH3-6), lincRNA	hs|chr13:57711300-57711241	A_21_P0008168	552	483E-01	009	191E-02	563	029	191E-01
lnc-AL445989.1-4	lnc-AL445989.1-4:1	linc|LNCipedia lincRNA (lnc-AL445989.1-4), linc	hs|chr13:63167439-63167498	A_21_P0008070	567	363E-02	028	191E-02	558	015	608E-01
LINC00448	ENST00000438352	ens|long intergenic non-protein coding RNA 44	hs|chr13:63306128-63306069	A_21_P0011188	553	923E-01	001	191E-02	564	-001	976E-01
lnc-NDFIP2-12	lnc-NDFIP2-12:1	linc|LNCipedia lincRNA (lnc-NDFIP2-12), lincRN	hs|chr13:83229743-83229802	A_21_P0008097	539	444E-01	010	444E-02	557	-019	483E-01
MIR4500HG	ENST00000441617	ens|MIR4500 host gene (non-protein coding) [	hs|chr13:88237273-88237214	A_21_P0008008	550	598E-01	-007	191E-02	560	017	468E-01
lnc-HSP90AA1-10	lnc-HSP90AA1-10:1	linc|LNCipedia lincRNA (lnc-HSP90AA1-10), linc	hs|chr14:101985915-101985856	A_21_P0008493	547	155E-03	041	191E-02	572	001	976E-01
lnc-RP11-80A15.1.1-4	lnc-RP11-80A15.1.1-4:1	linc|LNCipedia lincRNA (lnc-RP11-80A15.1.1-4),	hs|chr14:25591130-25591189	A_21_P0008502	525	763E-02	-024	444E-02	574	-018	440E-01
LOC101927418	NR_110050	ref|Homo sapiens uncharacterized LOC101927	hs|chr14:45368248-45368189	A_32_P14457	710	303E-01	014	191E-02	577	013	618E-01
XLOC_l2_004180	TCONS_l2_00007711	linc|BROAD Institute lincRNA (XLOC_l2_00418(	hs|chr14:48697344-48697403	A_21_P0011241	597	173E-02	-031	444E-02	612	003	923E-01
lnc-CNIH-1	lnc-CNIH-1:1	linc|LNCipedia lincRNA (lnc-CNIH-1), lincRNA [l	hs|chr14:54854765-54854706	A_21_P0008441	547	213E-02	031	387E-03	564	007	832E-01
LINC00520	NR_026796	ref|Homo sapiens long intergenic non-protein (	hs|chr14:56247937-56247878	A_32_P189781	562	160E-03	-041	444E-02	553	002	958E-01
lnc-MAX-2	lnc-MAX-2:2	linc|LNCipedia lincRNA (lnc-MAX-2), lincRNA [ln	hs|chr14:65708938-65708879	A_21_P0008449	557	635E-01	006	106E-02	574	001	978E-01
ENST00000556662	ENST00000556662	Unknown	hs|chr14:66589381-66589440	A_21_P0008514	516	533E-01	008	444E-02	554	-003	923E-01
lnc-SEL1L-7	lnc-SEL1L-7:2	linc|LNCipedia lincRNA (lnc-SEL1L-7), lincRNA [l	hs|chr14:84369916-84369857	A_21_P0008468	587	357E-01	-012	444E-02	600	-002	959E-01
ITPK1-AS1	NR_002808	ref|Homo sapiens ITPK1 antisense RNA 1 (ITPK	hs|chr14:93538216-93538275	A_33_P3397955	550	849E-01	-003	191E-02	574	001	977E-01
lnc-CLMN-2	lnc-CLMN-2:1	linc|LNCipedia lincRNA (lnc-CLMN-2), lincRNA [	hs|chr14:95806020-95805961	A_21_P0008479	573	106E-01	022	326E-02	582	-018	491E-01
SPATA41	NR_028139	ref|Homo sapiens spermatogenesis associated	hs|chr15:100889532-100889473	A_33_P3421084	645	357E-03	038	191E-02	612	-062	248E-01
lnc-OR4M2-7	lnc-OR4M2-7:4	linc|LNCipedia lincRNA (lnc-OR4M2-7), lincRNA	hs|chr15:20508789-20508848	A_21_P0008581	559	284E-02	-029	106E-02	585	013	629E-01
NBEAP1	NR_027992	ref|Homo sapiens neurobeachin pseudogene 1	hs|chr15:20874916-20874857	A_33_P3305655	625	730E-01	005	444E-02	621	008	771E-01
lnc-POTEB-4	lnc-POTEB-4:1	linc|LNCipedia lincRNA (lnc-POTEB-4), lincRNA	hs|chr15:22525840-22525781	A_21_P0008704	550	414E-01	011	444E-02	572	022	375E-01
LOC283683	NR_040057	ref|Homo sapiens uncharacterized LOC283683	hs|chr15:23094949-23094890	A_21_P0011348	618	759E-01	-004	444E-02	547	-017	505E-01
IPW	NR_023915	ref|Homo sapiens imprinted in Prader-Willi syn	hs|chr15:25363441-25363500	A_19_P00321911	660	154E-01	019	191E-02	664	003	945E-01
lnc-TRPM1-1	lnc-TRPM1-1:2	linc|LNCipedia lincRNA (lnc-TRPM1-1), lincRNA	hs|chr15:31514746-31514687	A_21_P0008716	629	197E-01	017	191E-02	577	014	553E-01
lnc-VPS18-1	lnc-VPS18-1:2	linc|LNCipedia lincRNA (lnc-VPS18-1), lincRNA [	hs|chr15:41199979-41200038	A_21_P0008624	599	812E-02	-023	106E-02	620	037	115E-01
OIP5-AS1	NR_026757	ref|Homo sapiens OIP5 antisense RNA 1 (OIP5-	hs|chr15:41577459-41577518	A_21_P0008626	895	664E-01	006	191E-02	896	-006	835E-01
LOC145837	ENST00000558309	Unknown	hs|chr15:69760529-69760588	A_21_P0008558	547	524E-01	-009	444E-02	549	026	229E-01
LOC283731	NR_027073	ref|Homo sapiens uncharacterized LOC283731	hs|chr15:74419199-74419140	A_21_P0000518	544	145E-01	020	444E-02	578	034	281E-01
lnc-STRA6-1	lnc-STRA6-1:2	linc|LNCipedia lincRNA (lnc-STRA6-1), lincRNA [	hs|chr15:74448528-74448469	A_21_P0008757	611	162E-03	041	191E-02	576	-015	646E-01
LINC00052	NR_026869	ref|Homo sapiens long intergenic non-protein (	hs|chr15:88122667-88122726	A_23_P382094	570	839E-01	003	191E-02	574	-004	908E-01
lnc-CRTC3-1	lnc-CRTC3-1:1	linc|LNCipedia lincRNA (lnc-CRTC3-1), lincRNA [	hs|chr15:91061852-91061911	A_21_P0008672	548	741E-01	004	125E-03	550	014	614E-01
lnc-SLCO3A1-1	lnc-SLCO3A1-1:2	linc|LNCipedia lincRNA (lnc-SLCO3A1-1), lincRN	hs|chr15:92179190-92180063	A_21_P0008839	526	216E-02	030	191E-02	550	-004	904E-01
lnc-NR2F2-8	lnc-NR2F2-8:7	linc|LNCipedia lincRNA (lnc-NR2F2-8), lincRNA	hs|chr15:95995004-95995063	A_21_P0008685	575	957E-02	-022	444E-02	591	032	186E-01
lnc-CA7-3	lnc-CA7-3:2	linc|LNCipedia lincRNA (lnc-CA7-3), lincRNA [ln	hs|chr16:066929196-066929255	A_21_P0008960	545	207E-01	-017	444E-02	580	-001	986E-01

ENST00000572479	ENST00000572479	Unknown	hs|chr16:14512539-14504245	A_21_P0009014	555	200E-01	-017	444E-02	577	003	934E-01
LOC100507534	NR_110649	ref|Homo sapiens uncharacterized LOC100507	hs|chr16:47883683-47883624	A_21_P0008882	591	189E-01	-018	444E-02	587	025	293E-01
XLOC_l2_005503	TCONS_l2_00010196	linc|BROAD Institute lincRNA (XLOC_l2_00550	hs|chr16:56177559-56176583	A_21_P0011484	523	312E-01	014	326E-02	555	-021	360E-01
LINC01572	ENST00000561611	Unknown	hs|chr16:72460032-72459973	A_21_P0009056	631	394E-02	-027	444E-02	617	005	873E-01
lnc-MPHOSPH6-2	lnc-MPHOSPH6-2:2	linc|LNCipedia lincRNA (lnc-MPHOSPH6-2), linc	hs|chr16:82578814-82578755	A_21_P0009073	741	303E-01	-014	191E-02	643	025	293E-01
ENST00000563248	ENST00000563248	ens|Uncharacterized protein {ECO:0000313|Er	hs|chr16:83966196-83966255	A_19_P00318761	587	950E-01	-001	444E-02	612	028	257E-01
lnc-ZDHHC7-1	lnc-ZDHHC7-1:1	linc|LNCipedia lincRNA (lnc-ZDHHC7-1), lincRN	hs|chr16:84981969-84981910	A_21_P0009136	543	363E-01	012	191E-02	564	005	917E-01
lnc-JPH3-3	lnc-JPH3-3:7	linc|LNCipedia lincRNA (lnc-JPH3-3), lincRNA [ln	hs|chr16:87549478-87549537	A_21_P0008992	558	351E-01	-013	444E-02	582	001	975E-01
SNAI3-AS1	NR_024399	ref|Homo sapiens SNAI3 antisense RNA 1 (SNA	hs|chr16:88738261-88738320	A_19_P00319854	612	220E-01	-016	195E-03	613	028	201E-01
LOC100289580	NR_103774	ref|Homo sapiens uncharacterized LOC100289	hs|chr16:88805420-88805479	A_21_P0014337	567	291E-02	029	326E-02	583	003	918E-01
LOC101059954	ENST00000578280	Unknown	hs|chr17:038677044-038677103	A_21_P0009245	568	278E-01	015	191E-02	568	001	980E-01
MIR22HG	NR_028502	ref|Homo sapiens MIR22 host gene (non-prote	hs|chr17:1615641-1615582	A_24_P253723	998	132E-02	033	191E-02	919	025	317E-01
ENST00000456090	ENST00000456090	ens|Smith-Magenis syndrome chromosome re	hs|chr17:17578743-17578684	A_21_P0009187	611	356E-01	-012	106E-02	594	004	906E-01
XLOC_l2_006013	TCONS_l2_00011129	linc|BROAD Institute lincRNA (XLOC_l2_00601	hs|chr17:19342434-19342375	A_21_P0011575	510	961E-01	-001	262E-02	562	001	973E-01
XLOC_l2_006026	TCONS_l2_00011144	linc|BROAD Institute lincRNA (XLOC_l2_00602	hs|chr17:20640403-20640344	A_21_P0011581	571	471E-01	-010	444E-02	634	-002	968E-01
CCDC144NL-AS1	XR_424802	ref|PREDICTED: Homo sapiens uncharacterized	hs|chr17:20832987-20833046	A_21_P0009162	623	802E-01	003	444E-02	577	006	881E-01
TBC1D3P5	NR_033892	ref|Homo sapiens TBC1 domain family, membe	hs|chr17:25758247-25758306	A_24_P838947	707	384E-02	-028	444E-02	666	041	124E-01
lnc-TMEM98-1	lnc-TMEM98-1:2	linc|LNCipedia lincRNA (lnc-TMEM98-1), lincRN	hs|chr17:31271986-31276797	A_21_P0009389	619	191E-01	018	444E-02	613	-009	771E-01
MAPT-IT1	NR_024560	ref|Homo sapiens MAPT intronic transcript 1 (r	hs|chr17:43974067-43974126	A_21_P0014032	595	662E-01	-006	106E-02	610	013	689E-01
ENST00000576313	ENST00000576313	Unknown	hs|chr17:55156660-55156601	A_21_P0009339	587	187E-01	-018	444E-02	577	-005	879E-01
lnc-PTRH2-1	lnc-PTRH2-1:3	linc|LNCipedia lincRNA (lnc-PTRH2-1), lincRNA	hs|chr17:57506558-57506499	A_21_P0009340	523	537E-01	-008	444E-02	564	009	740E-01
ENST00000453722	ENST00000453722	ens|long intergenic non-protein coding RNA 51	hs|chr17:70381150-70381091	A_21_P0009202	563	113E-01	-021	444E-02	576	-014	582E-01
MYO15B	NR_003587	ref|Homo sapiens myosin XVB pseudogene (M	hs|chr17:73622248-73622307	A_32_P475513	735	313E-02	029	191E-02	608	012	708E-01
LOC100132174	NR_104134	ref|Homo sapiens uncharacterized LOC100132	hs|chr17:75719810-75719751	A_21_P0013967	560	237E-02	030	387E-03	575	003	923E-01
FLJ43681	NR_029406	ref|Homo sapiens ribosomal protein L23a pseu	hs|chr17:81188142-81188201	A_23_P256059	1364	163E-01	-019	195E-03	1019	-003	919E-01
ENST00000582531	ENST00000582531	Unknown	hs|chr18:010371454-010371396	A_21_P0009524	553	507E-01	009	191E-02	575	000	995E-01
lnc-ZNF397-1	lnc-ZNF397-1:1	linc|LNCipedia lincRNA (lnc-ZNF397-1), lincRNA	hs|chr18:032906768-032906827	A_21_P0009472	534	567E-01	-008	444E-02	550	-010	723E-01
OACYLP	ENST00000417918	ens|O-acyltransferase like, pseudogene [Sourc	hs|chr18:056735646-056735705	A_21_P0011649	515	837E-01	-003	444E-02	556	015	626E-01
LDLRAD4-AS1	NR_040031	ref|Homo sapiens LDLRAD4 antisense RNA 1 (L	hs|chr18:13420028-13419969	A_21_P0000886	578	280E-01	015	444E-02	588	009	743E-01
LOC101927229	NR_110743	ref|Homo sapiens uncharacterized LOC101927	hs|chr18:52773225-52773166	A_21_P0009426	585	116E-02	-033	444E-02	573	-006	859E-01
lnc-WDR7-8	lnc-WDR7-8:1	linc|LNCipedia lincRNA (lnc-WDR7-8), lincRNA [	hs|chr18:53506018-53506077	A_19_P00807614	568	205E-01	-017	444E-02	617	023	396E-01
lnc-CETN1-1	lnc-CETN1-1:1	linc|LNCipedia lincRNA (lnc-CETN1-1), lincRNA	hs|chr18:571010-571069	A_21_P0009436	511	913E-01	-001	444E-02	549	010	708E-01
ENST00000590968	ENST00000590968	Unknown	hs|chr18:59416056-59415997	A_19_P00318514	559	678E-01	006	444E-02	575	-010	712E-01
lnc-FBXO15-3	lnc-FBXO15-3:3	linc|LNCipedia lincRNA (lnc-FBXO15-3), lincRNA	hs|chr18:71351169-71351110	A_21_P0009580	543	615E-01	-007	444E-02	568	002	959E-01
ENST00000580487	ENST00000580487	Unknown	hs|chr18:76556541-76556482	A_21_P0009593	515	263E-01	-015	444E-02	564	-019	464E-01
XLOC_l2_006673	TCONS_l2_00012432	linc|BROAD Institute lincRNA (XLOC_l2_00667	hs|chr19:028283924-028283983	A_19_P00319057	520	708E-01	005	444E-02	580	-008	779E-01
lnc-S1PR2-1	lnc-S1PR2-1:1	linc|LNCipedia lincRNA (lnc-S1PR2-1), lincRNA [	hs|chr19:10349825-10349766	A_21_P0009739	697	493E-02	-026	444E-02	670	023	429E-01
LOC100507373	NR_045214	ref|Homo sapiens uncharacterized LOC100507	hs|chr19:14279599-14279658	A_21_P0014393	609	255E-02	030	191E-02	606	042	376E-01
XLOC_l2_006624	TCONS_l2_00012350	linc|BROAD Institute lincRNA (XLOC_l2_00662	hs|chr19:16147713-16147772	A_21_P0011677	532	223E-01	-016	444E-02	554	017	498E-01
lnc-ZNF85-1	lnc-ZNF85-1:1	linc|LNCipedia lincRNA (lnc-ZNF85-1), lincRNA [	hs|chr19:21166816-21166875	A_21_P0009692	533	309E-02	-029	444E-02	571	-026	265E-01
LINC00662	ENST00000586954	ens|long intergenic non-protein coding RNA 66	hs|chr19:28268277-28268218	A_19_P00804777	905	461E-02	027	191E-02	816	028	207E-01
lnc-ZNF404-1	lnc-ZNF404-1:4	linc|LNCipedia lincRNA (lnc-ZNF404-1), lincRNA	hs|chr19:44405166-44405107	A_21_P0009768	629	275E-03	-039	444E-02	584	020	411E-01
lnc-ZNF227-1	lnc-ZNF227-1:2	linc|LNCipedia lincRNA (lnc-ZNF227-1), lincRNA	hs|chr19:44700015-44700228	A_21_P0009722	595	108E-01	022	191E-02	597	007	846E-01
ENST00000426213	ENST00000426213	Unknown	hs|chr19:54703616-54703557	A_21_P0009674	563	558E-01	-008	444E-02	595	-007	802E-01
lnc-SAFB-1	lnc-SAFB-1:1	linc|LNCipedia lincRNA (lnc-SAFB-1), lincRNA [ln	hs|chr19:5559129-5559188	A_21_P0009659	537	394E-01	-011	444E-02	600	008	814E-01
XLOC_l2_006832	TCONS_l2_00012718	linc|BROAD Institute lincRNA (XLOC_l2_00683	hs|chr19:56122951-56123010	A_21_P0011716	616	498E-01	-009	106E-02	640	014	707E-01
ZSCAN18	NR_027135	ref|Homo sapiens zinc finger and SCAN domair	hs|chr19:58619077-58619018	A_21_P0000526	549	288E-01	-014	444E-02	580	-002	962E-01
EMR4P	NR_024075	ref|Homo sapiens egf-like module containing,	hs|chr19:6952815-6952756	A_33_P3247320	555	513E-01	009	191E-02	556	-012	623E-01
XLOC_l2_006575	TCONS_l2_00012262	linc|BROAD Institute lincRNA (XLOC_l2_00657	hs|chr19:7849592-7849651	A_21_P0011668	552	111E-02	-033	444E-02	584	031	178E-01
lnc-PTBP1-1	lnc-PTBP1-1:1	linc|LNCipedia lincRNA (lnc-PTBP1-1), lincRNA [	hs|chr19:786107-786166	A_21_P0009675	536	196E-03	040	191E-02	617	007	837E-01
lnc-TGFBRAP1-3	lnc-TGFBRAP1-3:1	linc|LNCipedia lincRNA (lnc-TGFBRAP1-3), lincR	hs|chr2:105719491-105719432	A_21_P0002481	571	745E-02	-024	444E-02	611	-007	869E-01
LOC100507334	NR_037626	ref|Homo sapiens two pore channel 3 pseudog	hs|chr2:111020268-111020327	A_21_P0011840	615	262E-02	-030	195E-03	579	006	846E-01
lnc-FOXD4L1-1	lnc-FOXD4L1-1:3	linc|LNCipedia lincRNA (lnc-FOXD4L1-1), lincRN	hs|chr2:114265783-114265842	A_21_P0002606	609	682E-02	-024	444E-02	629	003	939E-01
RPL23AP7	NR_000029	ref|Homo sapiens ribosomal protein L23a pseu	hs|chr2:114368843-114368816	A_24_P153043	1465	281E-02	-029	106E-02	1112	015	703E-01
LINC00570	NR_047499	ref|Homo sapiens long intergenic non-protein	hs|chr2:11542175-11542234	A_21_P0011999	647	263E-03	039	191E-02	579	-008	775E-01
lnc-GLI2-4	lnc-GLI2-4:2	linc|LNCipedia lincRNA (lnc-GLI2-4), lincRNA [ln	hs|chr2:121339490-121339549	A_21_P0002279	521	589E-01	-007	191E-02	542	003	928E-01
lnc-POTEF-3	lnc-POTEF-3:1	linc|LNCipedia lincRNA (lnc-POTEF-3), lincRNA	hs|chr2:130343809-130343750	A_21_P0002504	526	406E-01	011	191E-02	557	011	755E-01
XLOC_l2_007571	TCONS_l2_00014004	linc|BROAD Institute lincRNA (XLOC_l2_00757	hs|chr2:136758031-136758090	A_21_P0011856	584	161E-01	-019	444E-02	617	011	697E-01
LOC101926966	NR_110201	ref|Homo sapiens uncharacterized LOC101926	hs|chr2:15858978-15859037	A_21_P0001770	550	914E-03	-034	444E-02	550	-002	955E-01
TTC21B-AS1	NR_038983	ref|Homo sapiens TTC21B antisense RNA 1 (TT	hs|chr2:166804712-166804771	A_21_P0000861	535	117E-01	-021	106E-02	566	006	837E-01
ENST00000448650	ENST00000448650	gb|DA532438 FEBRA2 Homo sapiens cDNA clo	hs|chr2:16948692-16948751	A_21_P0001838	502	453E-01	-010	106E-02	551	-006	859E-01
ENST00000414394	ENST00000414394	Unknown	hs|chr2:193510200-193510259	A_21_P0001948	676	922E-02	-023	444E-02	683	034	285E-01

LOC101927619	NR_110267	ref|Homo sapiens uncharacterized LOC101927	hs|chr2:199239284-199239225	A_21_P0002132	557	550E-01	008	191E-02	569	-016	563E-01
lnc-HS1BP3-1	lnc-HS1BP3-1:8	linc|LNCipedia lincRNA (lnc-HS1BP3-1), lincRNA	hs|chr2:20739256-20739197	A_21_P0002650	674	152E-01	-019	106E-02	689	-030	197E-01
lnc-CPO-1	lnc-CPO-1:4	linc|LNCipedia lincRNA (lnc-CPO-1), lincRNA [ln	hs|chr2:207791035-207791094	A_21_P0002624	539	505E-01	009	106E-02	543	005	899E-01
lnc-CCDC140-5	lnc-CCDC140-5:1	linc|LNCipedia lincRNA (lnc-CCDC140-5), lincRN	hs|chr2:222548206-222548265	A_21_P0002360	570	654E-01	006	444E-02	575	-001	975E-01
lnc-KLHL29-2	lnc-KLHL29-2:1	linc|LNCipedia lincRNA (lnc-KLHL29-2), lincRNA	hs|chr2:22762634-22762693	A_21_P0002579	638	119E-01	021	326E-02	563	004	913E-01
lnc-WDR69-1	lnc-WDR69-1:1	linc|LNCipedia lincRNA (lnc-WDR69-1), lincRNA	hs|chr2:229379293-229379352	A_21_P0002367	530	185E-02	-031	444E-02	553	-009	799E-01
lnc-SP140-3	lnc-SP140-3:1	linc|LNCipedia lincRNA (lnc-SP140-3), lincRNA [	hs|chr2:230990233-230993725	A_21_P0002370	545	223E-01	016	191E-02	561	003	928E-01
lnc-ATAD2B-1	lnc-ATAD2B-1:1	linc|LNCipedia lincRNA (lnc-ATAD2B-1), lincRN/	hs|chr2:23958444-23958385	A_21_P0002415	654	115E-02	033	191E-02	657	024	524E-01
LOC150935	NR_037808	ref|Homo sapiens uncharacterized LOC150935	hs|chr2:240721748-240721807	A_33_P3356080	530	190E-01	-018	444E-02	565	005	868E-01
lnc-AC074091.13.1-1	lnc-AC074091.13.1-1:1	linc|LNCipedia lincRNA (lnc-AC074091.13.1-1),	hs|chr2:27958295-27958094	A_21_P0002416	641	868E-01	-002	444E-02	592	-003	934E-01
ENST00000416685	ENST00000416685	Unknown	hs|chr2:306130-306071	A_21_P0001985	535	805E-01	003	191E-02	554	008	847E-01
lnc-LCLAT1-2	lnc-LCLAT1-2:1	linc|LNCipedia lincRNA (lnc-LCLAT1-2), lincRNA	hs|chr2:31082978-31083037	A_21_P0002194	574	365E-01	012	191E-02	575	012	664E-01
lnc-RPS7-1	lnc-RPS7-1:6	linc|LNCipedia lincRNA (lnc-RPS7-1), lincRNA [ln	hs|chr2:3606322-3608947	A_21_P0002160	673	627E-02	-025	444E-02	632	-016	512E-01
LINC01304	NR_037881	ref|Homo sapiens long intergenic non-protein	hs|chr2:4007668-4007609	A_21_P0001783	538	528E-01	009	191E-02	561	004	902E-01
lnc-PLEKHH2-2	lnc-PLEKHH2-2:1	linc|LNCipedia lincRNA (lnc-PLEKHH2-2), lincRN	hs|chr2:43415560-43433742	A_21_P0002206	586	813E-03	-035	444E-02	614	014	706E-01
ENST00000423539	ENST00000423539	Unknown	hs|chr2:64313566-64313507	A_21_P0002056	539	828E-01	003	444E-02	556	011	662E-01
lnc-LGALSL-1	lnc-LGALSL-1:2	linc|LNCipedia lincRNA (lnc-LGALSL-1), lincRNA	hs|chr2:64501080-64501139	A_21_P0002596	546	342E-01	-013	444E-02	572	026	435E-01
LOC339807	NR_034023	ref|Homo sapiens uncharacterized LOC339807	hs|chr2:64840085-64840144	A_21_P0002226	634	376E-01	-012	444E-02	625	028	333E-01
LOC101927533	ENST00000441506	ref|PREDICTED: Homo sapiens uncharacterized	hs|chr2:65981906-65981965	A_21_P0002231	530	385E-03	-038	106E-02	562	028	251E-01
lnc-ACTR2-3	lnc-ACTR2-3:10	linc|LNCipedia lincRNA (lnc-ACTR2-3), lincRNA	hs|chr2:65981908-66015822	A_21_P0002597	547	140E-01	020	387E-03	563	-006	828E-01
lnc-MEIS1-3	lnc-MEIS1-3:2	linc|LNCipedia lincRNA (lnc-MEIS1-3), lincRNA [	hs|chr2:66921035-66921094	A_21_P0002233	504	764E-01	004	444E-02	565	022	397E-01
ENST00000618608	ENST00000618608	Unknown	hs|chr2:67488184-67488243	A_19_P00317563	520	484E-01	009	444E-02	568	-022	448E-01
XLOC_l2_007876	TCONS_l2_00014406	linc|BROAD Institute lincRNA (XLOC_l2_00787	hs|chr2:7199849-7199790	A_21_P0011899	539	222E-01	-016	444E-02	562	007	830E-01
lnc-EXOC6B-1	lnc-EXOC6B-1:1	linc|LNCipedia lincRNA (lnc-EXOC6B-1), lincRNA	hs|chr2:73090729-73090670	A_21_P0002459	532	615E-01	007	444E-02	571	013	679E-01
LOC101927948	NR_110287	ref|Homo sapiens uncharacterized LOC101927	hs|chr2:78354773-78354832	A_21_P0002245	570	738E-01	005	444E-02	582	016	496E-01
LOC100630918	NR_038942	ref|Homo sapiens uncharacterized LOC100630	hs|chr2:85764776-85764717	A_21_P0000847	748	185E-01	-018	106E-02	597	022	732E-01
lnc-TMEM18-11	lnc-TMEM18-11:1	linc|LNCipedia lincRNA (lnc-TMEM18-11), lincR	hs|chr2:916706-907037	A_21_P0001989	613	364E-01	-012	444E-02	639	040	228E-01
ENST00000437696	ENST00000437696	Unknown	hs|chr20:22665925-22665866	A_19_P00321223	587	120E-02	-033	106E-02	579	-005	872E-01
lnc-SSTR4-4	lnc-SSTR4-4:3	linc|LNCipedia lincRNA (lnc-SSTR4-4), lincRNA [	hs|chr20:22666505-22671141	A_21_P0010086	564	126E-01	020	191E-02	563	-023	365E-01
LINC00028	NR_024358	ref|Homo sapiens long intergenic non-protein	hs|chr20:30075307-30075366	A_33_P3411392	586	104E-01	-022	444E-02	603	-010	708E-01
lnc-BPIFA2-1	lnc-BPIFA2-1:1	linc|LNCipedia lincRNA (lnc-BPIFA2-1), lincRNA	hs|chr20:31729369-31733749	A_21_P0009954	531	500E-01	009	191E-02	573	009	771E-01
LINC01370	NR_109936	ref|Homo sapiens long intergenic non-protein	hs|chr20:38635014-38635073	A_21_P0012121	543	555E-01	008	191E-02	563	006	829E-01
LINC01522	NR_110027	ref|Homo sapiens long intergenic non-protein	hs|chr20:46617383-46609496	A_21_P0009904	534	999E-01	000	444E-02	562	006	885E-01
lnc-CEBPB-6	lnc-CEBPB-6:1	linc|LNCipedia lincRNA (lnc-CEBPB-6), lincRNA		hs|chr20:48918429-48918488	A_21_P0010095	549	434E-02	-027	444E-02	558	-002	954E-01
LOC100506175	NR_111906	ref|Homo sapiens uncharacterized LOC100506	hs|chr20:49276575-49276634	A_21_P0014406	532	386E-02	-027	444E-02	560	-013	654E-01
lnc-TSHZ2-4	lnc-TSHZ2-4:1	linc|LNCipedia lincRNA (lnc-TSHZ2-4), lincRNA [	hs|chr20:52464678-52464737	A_21_P0009977	546	880E-03	-035	444E-02	562	012	658E-01
lnc-C20orf196-2	lnc-C20orf196-2:1	linc|LNCipedia lincRNA (lnc-C20orf196-2), lincR	hs|chr20:5479042-5479101	A_21_P0010079	626	652E-02	-025	444E-02	634	006	832E-01
ENST00000442780	ENST00000442780	Unknown	hs|chr20:55682125-55682184	A_21_P0009864	648	348E-01	-013	444E-02	623	020	385E-01
ENST00000603095	ENST00000603095	gb|Homo sapiens cDNA clone IMAGE:4131853	hs|chr20:57331823-57331882	A_21_P0012153	608	143E-02	-032	444E-02	639	029	222E-01
lnc-SYCP2-2	lnc-SYCP2-2:1	linc|LNCipedia lincRNA (lnc-SYCP2-2), lincRNA [	hs|chr20:58042998-58042939	A_21_P0010067	519	286E-01	014	326E-02	560	-033	157E-01
LOC100506470	NR_109919	ref|Homo sapiens uncharacterized LOC100506	hs|chr20:59655150-59655209	A_21_P0009986	535	567E-01	-008	444E-02	593	003	943E-01
LINC00659	NR_046224	ref|Homo sapiens long intergenic non-protein	hs|chr20:61405827-61405768	A_19_P00315651	602	114E-01	-021	106E-02	593	000	998E-01
LINC00320	NR_109786	ref|Homo sapiens long intergenic non-protein	hs|chr21:22116117-22116058	A_21_P0010183	598	320E-02	-029	106E-02	630	027	224E-01
LINC00515	NR_024092	ref|Homo sapiens long intergenic non-protein	hs|chr21:26955240-26955181	A_32_P149060	553	641E-01	006	444E-02	552	-010	719E-01
lnc-CYYR1-1	lnc-CYYR1-1:1	linc|LNCipedia lincRNA (lnc-CYYR1-1), lincRNA [	hs|chr21:27805057-27804998	A_21_P0010319	550	155E-02	032	191E-02	579	-007	828E-01
PAXBP1-AS1	NR_038880	ref|Homo sapiens PAXBP1 antisense RNA 1 (PA	hs|chr21:34105163-34105222	A_21_P0000824	572	165E-03	-041	444E-02	563	-015	577E-01
lnc-ITSN1-2	lnc-ITSN1-2:5	linc|LNCipedia lincRNA (lnc-ITSN1-2), lincRNA [	hs|chr21:35350604-35350663	A_19_P00812310	674	773E-01	-004	444E-02	577	007	833E-01
XLOC_l2_009216	TCONS_l2_00017233	linc|BROAD Institute lincRNA (XLOC_l2_00921	hs|chr21:40249231-40249172	A_21_P0012202	542	151E-03	041	387E-03	568	005	881E-01
XLOC_l2_009219	TCONS_l2_00017237	linc|BROAD Institute lincRNA (XLOC_l2_00921	hs|chr21:40360460-40360401	A_21_P0012204	617	552E-01	-008	444E-02	638	-004	946E-01
LINC00479	NR_027272	ref|Homo sapiens long intergenic non-protein	hs|chr21:43133093-43133034	A_33_P3239467	535	253E-02	030	191E-02	551	010	708E-01
lnc-WDR4-1	lnc-WDR4-1:1	ref|PREDICTED: Homo sapiens collagen alpha-3	hs|chr21:44257001-44256942	A_21_P0010322	719	775E-02	024	191E-02	657	-012	705E-01
LINC01424	NR_109928	ref|Homo sapiens long intergenic non-protein	hs|chr21:46223868-46223927	A_21_P0010262	570	145E-03	-041	444E-02	577	-029	244E-01
LINC00334	BM686390	gb|UI-E-CR0-adm-a-05-0-UI.r1 UI-E-CR0 Homo	hs|chr21:46678625-46678566	A_33_P3792489	541	325E-01	-013	444E-02	554	017	533E-01
LINC00316	NR_103811	ref|Homo sapiens long intergenic non-protein	hs|chr21:46758566-46758507	A_33_P3452287	555	153E-01	019	191E-02	570	-008	798E-01
LOC642426	NR_046104	ref|Homo sapiens uncharacterized LOC642426	hs|chr22:16420308-16420367	A_33_P3859499	566	830E-01	003	444E-02	592	004	936E-01
lnc-AC007663.1-1	lnc-AC007663.1-1:1	linc|LNCipedia lincRNA (lnc-AC007663.1-1), lin	hs|chr22:20188487-20188546	A_21_P0010342	555	796E-02	-023	444E-02	642	-020	388E-01
LL22NC03-63E9.3	NR_027426	ref|Homo sapiens uncharacterized LOC648691	hs|chr22:22908948-22909007	A_33_P3337124	548	984E-03	-034	106E-02	585	016	493E-01
XLOC_l2_009332	TCONS_l2_00017670	linc|BROAD Institute lincRNA (XLOC_l2_00933	hs|chr22:25905430-25905489	A_21_P0012266	529	958E-02	-022	444E-02	561	-005	859E-01
ENST00000430449	ENST00000430449	Unknown	hs|chr22:32366222-32366281	A_21_P0010353	551	308E-01	014	444E-02	580	-011	712E-01
LINC01315	NR_120595	ref|Homo sapiens long intergenic non-protein	hs|chr22:42760626-42760567	A_23_P166508	932	308E-03	039	191E-02	690	-018	493E-01
LINC01310	NR_038944	ref|Homo sapiens long intergenic non-protein	hs|chr22:49290848-49290907	A_33_P3384502	596	382E-01	-012	444E-02	601	020	438E-01
LINC00883	NR_028302	ref|Homo sapiens long intergenic non-protein	hs|chr3:107010635-107010694	A_19_P00319709	535	633E-01	006	191E-02	566	-013	648E-01

XLOC_l2_009811	TCONS_l2_00018728	linc|BROAD Institute lincRNA (XLOC_l2_00981	hs|chr3:107151962-107152021	A_21_P0012387	570	288E-01	-014	444E-02	593	003	932E-01
LINC01215	NR_110028	ref|Homo sapiens long intergenic non-protein	hs|chr3:107856895-107856954	A_19_P00809368	559	312E-01	-014	444E-02	563	-001	987E-01
lnc-RETNLB-1	lnc-RETNLB-1:2	linc|LNCipedia lincRNA (lnc-RETNLB-1), lincRNA	hs|chr3:108462221-108462162	A_21_P0003293	619	104E-03	043	191E-02	572	011	710E-01
PVRL3-AS1	NR_045114	ref|Homo sapiens PVRL3 antisense RNA 1 (PVR	hs|chr3:110764253-110764194	A_21_P0014107	584	821E-01	-003	444E-02	587	021	356E-01
lnc-FBLN2-1	lnc-FBLN2-1:3	linc|LNCipedia lincRNA (lnc-FBLN2-1), lincRNA	hs|chr3:13759172-13759231	A_21_P0002932	1017	180E-01	018	191E-02	1084	-039	217E-01
ENST00000492731	ENST00000492731	Unknown	hs|chr3:151360563-151360622	A_21_P0012408	563	352E-03	038	191E-02	564	-001	980E-01
ENST00000498604	ENST00000498604	Unknown	hs|chr3:154229384-154229325	A_21_P0002893	571	242E-01	-016	444E-02	604	-007	810E-01
LINC00880	NR_034007	ref|Homo sapiens long intergenic non-protein	hs|chr3:156800853-156800794	A_19_P00315790	521	425E-02	-027	444E-02	557	016	561E-01
ENST00000498241	ENST00000498241	Unknown	hs|chr3:157734866-157734187	A_21_P0002898	542	202E-02	031	598E-04	583	037	140E-01
LINC01192	ENST00000489012	ens|long intergenic non-protein coding RNA 11	hs|chr3:162925515-162925456	A_21_P0012553	516	352E-01	013	733E-03	562	-007	843E-01
LINC01322	NR_125764	ref|Homo sapiens long intergenic non-protein	hs|chr3:165208995-165209054	A_19_P00323040	558	800E-01	-003	191E-02	615	009	803E-01
lnc-OXNAD1-2	lnc-OXNAD1-2:3	linc|LNCipedia lincRNA (lnc-OXNAD1-2), lincRN	hs|chr3:16580344-16580403	A_21_P0003221	578	319E-01	013	191E-02	560	-001	984E-01
LOC100128164	NR_027622	ref|Homo sapiens four and a half LIM domains	hs|chr3:169661931-169661872	A_21_P0000541	575	125E-02	-033	444E-02	598	013	612E-01
LOC101928790	NR_110059	ref|Homo sapiens uncharacterized LOC101928	hs|chr3:179639606-179639665	A_21_P0014130	534	798E-01	003	191E-02	566	-010	792E-01
LINC01206	NR_104146	ref|Homo sapiens long intergenic non-protein	hs|chr3:181717981-181718040	A_21_P0012516	559	142E-01	-020	444E-02	644	040	216E-01
lnc-KCNH8-1	lnc-KCNH8-1:1	linc|LNCipedia lincRNA (lnc-KCNH8-1), lincRNA	hs|chr3:18948263-18959166	A_21_P0002727	606	375E-02	-028	444E-02	604	017	537E-01
XLOC_l2_010489	TCONS_l2_00019667	linc|BROAD Institute lincRNA (XLOC_l2_01048	hs|chr3:193310830-193308119	A_21_P0012475	571	309E-01	-014	444E-02	572	004	907E-01
lnc-CPN2-2	lnc-CPN2-2:4	linc|LNCipedia lincRNA (lnc-CPN2-2), lincRNA [l	hs|chr3:193919557-193919498	A_21_P0003205	565	149E-01	-019	444E-02	587	026	312E-01
lnc-HES1-3	lnc-HES1-3:2	linc|LNCipedia lincRNA (lnc-HES1-3), lincRNA [l	hs|chr3:193975823-193975882	A_19_P00809455	619	622E-01	-007	444E-02	631	030	228E-01
lnc-NKIRAS1-3	lnc-NKIRAS1-3:1	linc|LNCipedia lincRNA (lnc-NKIRAS1-3), lincRN	hs|chr3:23236441-23236382	A_21_P0003106	502	480E-02	026	191E-02	551	005	891E-01
lnc-OXSM-1	lnc-OXSM-1:2	linc|LNCipedia lincRNA (lnc-OXSM-1), lincRNA [	hs|chr3:26020280-26020339	A_21_P0003224	543	536E-01	008	191E-02	555	005	872E-01
lnc-AZI2-1	lnc-AZI2-1:1	linc|LNCipedia lincRNA (lnc-AZI2-1), lincRNA [l	hs|chr3:28180572-28179945	A_21_P0003108	540	313E-01	-014	444E-02	559	000	999E-01
LINC00693	NR_038840	ref|Homo sapiens long intergenic non-protein	hs|chr3:28617764-28617823	A_21_P0012370	605	121E-02	033	191E-02	606	003	920E-01
lnc-PRSS42-1	lnc-PRSS42-1:1	linc|LNCipedia lincRNA (lnc-PRSS42-1), lincRNA	hs|chr3:46877551-46877492	A_21_P0003115	582	151E-02	-032	444E-02	558	010	714E-01
lnc-KLHL18-1	lnc-KLHL18-1:1	linc|LNCipedia lincRNA (lnc-KLHL18-1), lincRNA	hs|chr3:47206072-47206657	A_21_P0002956	552	182E-03	-040	444E-02	563	001	989E-01
lnc-RFT1-1	lnc-RFT1-1:1	linc|LNCipedia lincRNA (lnc-RFT1-1), lincRNA [l	hs|chr3:53119801-53112204	A_21_P0003119	539	623E-01	-007	106E-02	548	002	969E-01
ENST00000497258	ENST00000497258	Unknown	hs|chr3:59365812-59365753	A_21_P0002856	540	332E-01	-013	444E-02	601	001	976E-01
lnc-ADAMTS9-1	lnc-ADAMTS9-1:1	linc|LNCipedia lincRNA (lnc-ADAMTS9-1), lincR	hs|chr3:64474706-64474647	A_21_P0003123	526	418E-01	-011	444E-02	556	012	678E-01
LINC00870	ENST00000469178	ens|long intergenic non-protein coding RNA 87	hs|chr3:72202010-72202069	A_19_P00319765	579	647E-01	-006	444E-02	611	021	409E-01
LOC101927394	NR_110131	ref|Homo sapiens uncharacterized LOC101927	hs|chr3:7995131-7995072	A_21_P0012534	512	304E-01	-014	444E-02	550	-017	483E-01
lnc-CADM2-5	lnc-CADM2-5:1	linc|LNCipedia lincRNA (lnc-CADM2-5), lincRNA	hs|chr3:83281586-83281645	A_21_P0002988	544	883E-02	023	191E-02	560	021	391E-01
lnc-CADM2-4	lnc-CADM2-4:2	linc|LNCipedia lincRNA (lnc-CADM2-4), lincRNA	hs|chr3:83486635-83486694	A_21_P0002989	572	948E-03	034	191E-02	576	-007	813E-01
LINC00971	ENST00000491849	ens|long intergenic non-protein coding RNA 97	hs|chr3:84917356-84917297	A_21_P0012545	583	195E-02	031	387E-03	575	010	734E-01
ENST00000509306	ENST00000509306	ens|olfactory receptor, family 5, subfamily AC,	hs|chr3:97823608-97823667	A_21_P0012384	538	601E-02	-025	444E-02	553	001	987E-01
lnc-RAB28-3	lnc-RAB28-3:1	linc|LNCipedia lincRNA (lnc-RAB28-3), lincRNA	hs|chr4:012550453-012550394	A_21_P0003723	569	291E-01	014	191E-02	574	-007	821E-01
ENST00000504132	ENST00000504132	Unknown	hs|chr4:107370176-107370235	A_21_P0003843	533	455E-01	010	191E-02	569	026	413E-01
ENST00000512039	ENST00000512039	Unknown	hs|chr4:137734865-137734806	A_21_P0003513	536	198E-01	017	191E-02	580	012	651E-01
DKFZP434I0714	NR_033797	ref|Homo sapiens uncharacterized protein DKF	hs|chr4:153459619-153459678	A_23_P413303	576	872E-03	-034	444E-02	562	-030	180E-01
lnc-CRIPAK-1	lnc-CRIPAK-1:7	linc|LNCipedia lincRNA (lnc-CRIPAK-1), lincRNA	hs|chr4:1553384-1553517	A_21_P0003825	655	124E-02	033	191E-02	585	-014	664E-01
TAPT1-AS1	NR_027696	ref|Homo sapiens TAPT1 antisense RNA 1 (hea	hs|chr4:16259107-16259166	A_19_P00328190	709	872E-02	-023	106E-02	716	032	370E-01
lnc-CLCN3-1	lnc-CLCN3-1:3	linc|LNCipedia lincRNA (lnc-CLCN3-1), lincRNA	hs|chr4:170861125-170861184	A_21_P0003858	586	475E-02	-026	444E-02	691	033	216E-01
XLOC_l2_010947	TCONS_l2_00020894	linc|BROAD Institute lincRNA (XLOC_l2_01094	hs|chr4:174847092-174850418	A_21_P0012636	541	998E-01	000	444E-02	552	-013	606E-01
lnc-VEGFC-1	lnc-VEGFC-1:1	linc|LNCipedia lincRNA (lnc-VEGFC-1), lincRNA	hs|chr4:177567741-177567682	A_21_P0003801	526	680E-01	-006	106E-02	556	-016	633E-01
XLOC_l2_010963	TCONS_l2_00020917	linc|BROAD Institute lincRNA (XLOC_l2_01096	hs|chr4:183956600-183956659	A_21_P0012638	550	354E-01	012	387E-03	571	005	883E-01
XLOC_l2_010976	TCONS_l2_00020934	linc|BROAD Institute lincRNA (XLOC_l2_01097	hs|chr4:185977196-185977255	A_21_P0012642	521	389E-01	-012	444E-02	554	009	741E-01
lnc-SORBS2-1	lnc-SORBS2-1:1	linc|LNCipedia lincRNA (lnc-SORBS2-1), lincRNA	hs|chr4:186491393-186491334	A_21_P0003817	524	861E-03	-034	444E-02	552	-018	451E-01
LOC100506272	NR_110436	ref|Homo sapiens uncharacterized LOC100506	hs|chr4:188454177-188454118	A_33_P3390591	522	386E-02	027	191E-02	561	-004	911E-01
LOC100133461	NR_034136	ref|Homo sapiens uncharacterized LOC100133	hs|chr4:3675402-3675343	A_33_P3255459	580	569E-01	008	191E-02	562	018	442E-01
LOC101060498	NR_121640	ref|Homo sapiens uncharacterized LOC101060	hs|chr4:40332375-40332434	A_33_P3310774	637	329E-02	028	598E-04	573	-014	562E-01
lnc-PDE6B-1	lnc-PDE6B-1:9	linc|LNCipedia lincRNA (lnc-PDE6B-1), lincRNA	hs|chr4:582501-582560	A_21_P0003556	566	258E-01	-015	444E-02	620	011	735E-01
FLJ36777	NR_027441	ref|Homo sapiens uncharacterized LOC730971	hs|chr4:7099308-7099249	A_21_P0003313	524	105E-02	-034	444E-02	547	002	956E-01
EPB41L4A-AS2	NR_027706	ref|Homo sapiens EPB41L4A antisense RNA 2 (	hs|chr5:111756497-111756556	A_23_P69941	643	117E-02	033	444E-02	559	-016	538E-01
CTD-3080P12.3	NR_109911	ref|Homo sapiens uncharacterized LOC101928	hs|chr5:1176149-1176090	A_19_P00317835	716	751E-01	-004	444E-02	728	011	785E-01
ENST00000513329	ENST00000513329	Unknown	hs|chr5:133827910-133827969	A_19_P00319133	568	366E-01	-012	444E-02	573	-016	512E-01
LINC01511	NR_125810	ref|Homo sapiens long intergenic non-protein	hs|chr5:1364346-1364287	A_21_P0012969	589	160E-02	-032	444E-02	560	007	869E-01
lnc-C5orf32-1	lnc-C5orf32-1:8	linc|LNCipedia lincRNA (lnc-C5orf32-1), lincRNA	hs|chr5:139553546-139553605	A_21_P0004530	535	279E-01	015	191E-02	562	006	862E-01
lnc-PRELID2-2	lnc-PRELID2-2:1	linc|LNCipedia lincRNA (lnc-PRELID2-2), lincRN	hs|chr5:144775587-144769380	A_21_P0004453	559	706E-03	035	191E-02	576	014	594E-01
ENST00000503113	ENST00000503113	Unknown	hs|chr5:1544320-1544261	A_21_P0012970	603	508E-01	009	444E-02	570	020	407E-01
lnc-UBLCP1-8	lnc-UBLCP1-8:1	linc|LNCipedia lincRNA (lnc-UBLCP1-8), lincRNA	hs|chr5:159012833-159012892	A_21_P0004322	520	734E-01	005	387E-03	574	-024	439E-01
lnc-CCDC99-1	lnc-CCDC99-1:1	linc|LNCipedia lincRNA (lnc-CCDC99-1), lincRN	hs|chr5:168896210-168926516	A_21_P0004328	521	623E-01	-007	444E-02	548	-007	823E-01
LINC01366	NR_026945	ref|Homo sapiens long intergenic non-protein	hs|chr5:169760316-169760375	A_21_P0004329	569	699E-02	024	191E-02	565	005	869E-01
lnc-BASP1-3	lnc-BASP1-3:10	linc|LNCipedia lincRNA (lnc-BASP1-3), lincRNA	hs|chr5:17412140-17412199	A_21_P0004491	510	894E-01	-002	191E-02	550	-010	733E-01

XLOC_l2_012323	TCONS_l2_00023265	linc|BROAD Institute lincRNA (XLOC_l2_012323	hs|chr5:175598926-175598867	A_21_P0012934	524	127E-01	-020	444E-02	557	009	785E-01
LINC01574	NR_108030	ref|Homo sapiens long intergenic non-protein	hs|chr5:176170801-176170860	A_21_P0004343	572	550E-01	-008	106E-02	588	-009	807E-01
ENST00000502100	ENST00000502100	Unknown	hs|chr5:25302384-25302443	A_21_P0003980	546	242E-01	-016	191E-02	572	-023	366E-01
lnc-CDH6-2	lnc-CDH6-2:3	linc|LNCipedia lincRNA (lnc-CDH6-2), lincRNA [l	hs|chr5:29162715-29162774	A_21_P0004238	549	905E-01	002	444E-02	563	-030	368E-01
LOC100506674	NR_109862	ref|Homo sapiens uncharacterized LOC100506	hs|chr5:44745199-44745140	A_21_P0012983	602	119E-01	021	444E-02	577	010	732E-01
ENST00000504349	ENST00000504349	Unknown	hs|chr5:55609791-55609732	A_21_P0004129	552	362E-01	-012	444E-02	574	-004	921E-01
lnc-NSUN2-1	lnc-NSUN2-1:4	linc|LNCipedia lincRNA (lnc-NSUN2-1), lincRNA	hs|chr5:6702853-6702794	A_21_P0004359	529	683E-01	006	444E-02	556	011	662E-01
lnc-PIK3R1-6	lnc-PIK3R1-6:1	linc|LNCipedia lincRNA (lnc-PIK3R1-6), lincRNA	hs|chr5:67976879-67976938	A_21_P0004262	522	235E-01	-016	444E-02	556	009	803E-01
lnc-ZNF366-2	lnc-ZNF366-2:3	linc|LNCipedia lincRNA (lnc-ZNF366-2), lincRNA	hs|chr5:71870167-71817327	A_21_P0004561	557	983E-02	022	191E-02	572	-011	663E-01
lnc-ADCY2-1	lnc-ADCY2-1:1	linc|LNCipedia lincRNA (lnc-ADCY2-1), lincRNA	hs|chr5:7391934-7391993	A_21_P0004223	531	607E-01	-007	444E-02	566	001	987E-01
DMGDH	NR_104002	ref|Homo sapiens dimethylglycine dehydrogen	hs|chr5:78317489-78317430	A_33_P3387716	541	730E-01	-005	387E-03	547	015	619E-01
lnc-MTRR-2	lnc-MTRR-2:1	linc|LNCipedia lincRNA (lnc-MTRR-2), lincRNA [l	hs|chr5:8052037-8052096	A_21_P0003966	579	412E-01	-011	444E-02	576	015	539E-01
lnc-POLR3G-1	lnc-POLR3G-1:2	linc|LNCipedia lincRNA (lnc-POLR3G-1), lincRNA	hs|chr5:89711863-89711922	A_21_P0004276	596	874E-01	-002	444E-02	579	005	869E-01
ENST00000506070	ENST00000506070	Unknown	hs|chr5:95305978-95300949	A_21_P0012999	521	223E-01	-016	444E-02	556	014	728E-01
lnc-HIST1H1A-1	lnc-HIST1H1A-1:1	linc|LNCipedia lincRNA (lnc-HIST1H1A-1), lincRI	hs|chr6:025997812-025997753	A_21_P0005014	545	155E-01	-019	444E-02	565	-017	585E-01
lnc-OLIG3-1	lnc-OLIG3-1:1	linc|LNCipedia lincRNA (lnc-OLIG3-1), lincRNA [l	hs|chr6:137994354-137994295	A_21_P0005061	576	108E-01	-022	444E-02	609	004	922E-01
LOC100130476	NR_049793	ref|Homo sapiens uncharacterized LOC100130	hs|chr6:138144988-138144929	A_19_P00317547	584	186E-01	-018	191E-02	554	-028	255E-01
ENST00000421237	ENST00000421237	Unknown	hs|chr6:143277594-143267285	A_21_P0004784	592	253E-01	015	444E-02	584	006	832E-01
XLOC_l2_012748	TCONS_l2_00024458	linc|BROAD Institute lincRNA (XLOC_l2_012748	hs|chr6:143278231-143278290	A_21_P0013043	530	694E-01	005	195E-03	567	-013	714E-01
ENST00000436953	ENST00000436953	Unknown	hs|chr6:153625846-153625787	A_21_P0005179	538	249E-01	-016	106E-02	583	003	946E-01
LOC729603	NR_003288	ref|Homo sapiens calcineurin-like EF-hand prot	hs|chr6:160517125-160517184	A_33_P3410859	641	935E-02	-022	106E-02	570	000	996E-01
DKFZp451B082	NR_033862	ref|Homo sapiens uncharacterized LOC401282	hs|chr6:163759448-163759389	A_33_P3826751	513	769E-01	004	106E-02	562	-021	431E-01
ENST00000434596	ENST00000434596	Unknown	hs|chr6:168641336-168641395	A_21_P0004709	639	595E-01	-007	444E-02	595	009	738E-01
LINC00574	NR_026780	ref|Homo sapiens long intergenic non-protein	hs|chr6:170201802-170201861	A_23_P168165	580	501E-02	-026	444E-02	603	009	789E-01
lnc-FAM120B-6	lnc-FAM120B-6:2	linc|LNCipedia lincRNA (lnc-FAM120B-6), lincRI	hs|chr6:170455160-170455219	A_21_P0004977	546	150E-01	-019	195E-03	568	003	936E-01
lnc-GMDS-1	lnc-GMDS-1:1	linc|LNCipedia lincRNA (lnc-GMDS-1), lincRNA [	hs|chr6:2273117-2272911	A_21_P0004989	634	160E-01	-019	444E-02	657	023	374E-01
HCG11	NR_026790	ref|Homo sapiens HLA complex group 11 (non-	hs|chr6:26527499-26527558	A_33_P3281036	1058	322E-01	013	444E-02	677	017	497E-01
LOC285847	NR_027117	ref|Homo sapiens uncharacterized LOC285847	hs|chr6:35694692-35694633	A_21_P0000525	601	596E-03	-036	444E-02	620	025	311E-01
ENST00000444731	ENST00000444731	Unknown	hs|chr6:40239563-40239504	A_21_P0005023	775	337E-01	-013	444E-02	892	094	125E-01
TDRG1	NR_024015	ref|Homo sapiens testis development related 1	hs|chr6:40347350-40347409	A_19_P00322096	904	541E-01	008	191E-02	842	043	203E-01
lnc-TREM2-1	lnc-TREM2-1:1	linc|LNCipedia lincRNA (lnc-TREM2-1), lincRNA	hs|chr6:41144873-41144814	A_21_P0005025	626	764E-03	035	191E-02	585	-003	925E-01
lnc-SUPT3H-1	lnc-SUPT3H-1:1	linc|LNCipedia lincRNA (lnc-SUPT3H-1), lincRNA	hs|chr6:45535248-45535189	A_21_P0005029	545	261E-01	-015	444E-02	584	006	869E-01
lnc-UBE2CBP-1	lnc-UBE2CBP-1:1	linc|LNCipedia lincRNA (lnc-UBE2CBP-1), lincRN	hs|chr6:83386934-83386875	A_21_P0005038	538	963E-01	-001	444E-02	556	011	664E-01
lnc-GJA10-7	lnc-GJA10-7:1	linc|LNCipedia lincRNA (lnc-GJA10-7), lincRNA [	hs|chr6:93099369-93099428	A_21_P0004909	523	342E-01	013	191E-02	557	003	938E-01
lnc-GNB2-1	lnc-GNB2-1:1	linc|LNCipedia lincRNA (lnc-GNB2-1), lincRNA [l	hs|chr7:100264211-100264270	A_21_P0005195	583	444E-01	-010	444E-02	582	-010	787E-01
DOCK4-AS1	NR_103806	ref|Homo sapiens DOCK4 antisense RNA 1 (DO	hs|chr7:111461499-111461558	A_21_P0014196	546	126E-03	042	191E-02	565	000	997E-01
ST7-AS1	NR_002330	ref|Homo sapiens ST7 antisense RNA 1 (ST7-AS	hs|chr7:116592754-116592695	A_32_P181297	775	526E-01	-009	444E-02	572	018	477E-01
lnc-ARF5-5	lnc-ARF5-5:1	linc|LNCipedia lincRNA (lnc-ARF5-5), lincRNA [li	hs|chr7:125870412-125873594	A_21_P0005425	560	219E-01	017	191E-02	568	-021	462E-01
lnc-RP11-305M3.3.1-1	lnc-RP11-305M3.3.1-1:1	linc|LNCipedia lincRNA (lnc-RP11-305M3.3.1-1)	hs|chr7:129172020-129172079	A_21_P0005428	554	464E-01	010	387E-03	579	015	613E-01
JHDM1D-AS1	NR_024451	ref|Homo sapiens JHDM1D antisense RNA 1 (h	hs|chr7:139878860-139878919	A_32_P54503	626	727E-02	-024	444E-02	561	-005	893E-01
lnc-ARHGEF5-1	lnc-ARHGEF5-1:4	linc|LNCipedia lincRNA (lnc-ARHGEF5-1), lincRN	hs|chr7:144088043-144088102	A_21_P0005439	525	527E-02	-026	444E-02	552	-015	585E-01
ENST00000426187	ENST00000426187	Unknown	hs|chr7:156225567-156225508	A_21_P0005341	559	225E-01	-016	444E-02	585	-007	822E-01
LINC01006	NR_103858	ref|Homo sapiens long intergenic non-protein	hs|chr7:156265007-156264948	A_21_P0013460	672	880E-01	-002	444E-02	592	028	373E-01
LOC101927668	NR_110114	ref|Homo sapiens uncharacterized LOC101927	hs|chr7:19958663-19958604	A_19_P00315631	587	272E-03	039	191E-02	557	010	795E-01
ENST00000414127	ENST00000414127	Unknown	hs|chr7:25360984-25360925	A_21_P0005288	534	473E-01	-010	387E-03	564	-006	865E-01
lnc-KIAA0087-1	lnc-KIAA0087-1:1	linc|LNCipedia lincRNA (lnc-KIAA0087-1), lincRI	hs|chr7:26590507-26590448	A_21_P0005483	580	387E-02	-028	444E-02	643	031	239E-01
DPY19L2P1	NR_002833	ref|Homo sapiens DPY19L2 pseudogene 1 (DPY	hs|chr7:29781923-29781982	A_33_P3626301	609	221E-02	-030	195E-03	629	-006	870E-01
SEPT7-AS1	ENST00000424194	ens|SEPT7 antisense RNA 1 (head to head) [So	hs|chr7:35803444-35803385	A_21_P0013419	531	621E-01	007	191E-02	554	-011	683E-01
lnc-HERPUD2-4	lnc-HERPUD2-4:1	linc|LNCipedia lincRNA (lnc-HERPUD2-4), lincRI	hs|chr7:36015574-36015515	A_21_P0005490	556	590E-01	007	444E-02	566	-009	738E-01
ENST00000440935	ENST00000440935	Unknown	hs|chr7:46521104-46521045	A_21_P0005494	541	777E-01	-004	444E-02	562	004	901E-01
lnc-POM121L12-1	lnc-POM121L12-1:2	linc|LNCipedia lincRNA (lnc-POM121L12-1), linc	hs|chr7:52842474-52842533	A_21_P0005561	533	208E-01	017	191E-02	578	-015	654E-01
FLJ44511	NR_033963	ref|Homo sapiens uncharacterized LOC441307	hs|chr7:563781-563840	A_33_P3887888	565	114E-01	021	387E-03	571	-014	599E-01
lnc-TPST1-1	lnc-TPST1-1:4	linc|LNCipedia lincRNA (lnc-TPST1-1), lincRNA [	hs|chr7:65960517-65960576	A_21_P0005391	552	612E-01	007	191E-02	571	010	708E-01
LOC101927354	NR_108073	ref|Homo sapiens uncharacterized LOC101927	hs|chr7:7317328-7317387	A_21_P0005210	591	293E-01	-014	444E-02	586	006	870E-01
ENST00000416593	ENST00000416593	Unknown	hs|chr7:95103251-95103310	A_21_P0005247	599	161E-01	-019	444E-02	599	-016	519E-01
NACAP1	NR_002182	ref|Homo sapiens nascent-polypeptide-associa	hs|chr8:102381755-102381814	A_23_P59888	1105	544E-02	-026	444E-02	608	-013	613E-01
ERICH1-AS1	NR_073397	ref|Homo sapiens ERICH1 antisense RNA 1 (ER	hs|chr8:1087670-1087729	A_32_P930375	523	278E-01	-015	106E-02	557	004	924E-01
lnc-SLC45A4-1	lnc-SLC45A4-1:1	linc|LNCipedia lincRNA (lnc-SLC45A4-1), lincRN	hs|chr8:142208102-142208043	A_21_P0005915	706	279E-01	-015	444E-02	758	067	822E-02
lnc-PTP4A3-3	lnc-PTP4A3-3:2	linc|LNCipedia lincRNA (lnc-PTP4A3-3), lincRNA	hs|chr8:142336327-142336386	A_21_P0005775	571	567E-01	-008	444E-02	592	002	956E-01
ADAM3A	NR_073423	ref|Homo sapiens ADAM metallopeptidase dor	hs|chr8:39308659-39308600	A_33_P3256505	568	624E-02	025	191E-02	576	-001	972E-01
lnc-ZMAT4-2	lnc-ZMAT4-2:1	linc|LNCipedia lincRNA (lnc-ZMAT4-2), lincRNA	hs|chr8:40810882-40810823	A_21_P0005826	559	476E-01	-010	444E-02	574	004	920E-01
LOC100507651	NR_038235	ref|Homo sapiens uncharacterized LOC100507	hs|chr8:58145323-58145382	A_21_P0000730	547	565E-01	-008	106E-02	552	003	921E-01

lnc-HNF4G-1	lnc-HNF4G-1:1	linc|LNCipedia lincRNA (lnc-HNF4G-1), lincRNA	hs|chr8:76851968-76863780	A_21_P0005725	538	512E-01	009	191E-02	570	000	995E-01
REXO1L2P	NR_003594	ref|Homo sapiens REX1, RNA exonuclease 1 ho	hs|chr8:86788048-86787989	A_33_P3339915	618	216E-02	-030	106E-02	658	014	728E-01
OTUD6B-AS1	XR_432334	ref|PREDICTED: Homo sapiens uncharacterized	hs|chr8:92081834-92072924	A_21_P0013524	582	192E-01	-018	444E-02	571	-003	929E-01
lnc-POP1-1	lnc-POP1-1:1	linc|LNCipedia lincRNA (lnc-POP1-1), lincRNA [l	hs|chr8:99192077-99192136	A_21_P0005738	566	114E-01	021	191E-02	610	063	271E-01
lnc-COL15A1-1	lnc-COL15A1-1:1	linc|LNCipedia lincRNA (lnc-COL15A1-1), lincRN	hs|chr9:101691689-101691748	A_21_P0006189	514	219E-01	-017	444E-02	561	-017	497E-01
XLOC_l2_015315	TCONS_l2_00030039	linc|BROAD Institute lincRNA (XLOC_l2_01531!	hs|chr9:102122014-102121955	A_21_P0013737	544	790E-03	-035	444E-02	569	013	608E-01
lnc-TMEM38B-1	lnc-TMEM38B-1:1	gb|Homo sapiens, clone IMAGE:5538960, mRN	hs|chr9:109173237-109173296	A_21_P0005992	516	466E-01	-010	444E-02	559	011	681E-01
lnc-KLF4-4	lnc-KLF4-4:2	linc|LNCipedia lincRNA (lnc-KLF4-4), lincRNA [ln	hs|chr9:109864446-109864387	A_21_P0006100	549	397E-02	-027	195E-03	580	005	865E-01
lnc-DENND1A-1	lnc-DENND1A-1:1	linc|LNCipedia lincRNA (lnc-DENND1A-1), lincR	hs|chr9:126101257-126101198	A_21_P0006318	546	692E-01	-005	444E-02	577	017	462E-01
lnc-C9orf50-1	lnc-C9orf50-1:1	linc|LNCipedia lincRNA (lnc-C9orf50-1), lincRNA	hs|chr9:132324873-132324814	A_21_P0006329	539	470E-01	-010	106E-02	561	000	995E-01
LINC01451	ENST00000623792	ens|long intergenic non-protein coding RNA 14	hs|chr9:139511203-139511144	A_33_P3235282	603	724E-02	-024	444E-02	626	024	278E-01
LOC100128593	NR_033913	ref|Homo sapiens uncharacterized LOC100128	hs|chr9:139643349-139643408	A_33_P3264072	585	699E-03	-036	106E-02	563	001	974E-01
XLOC_l2_014711	TCONS_l2_00028630	linc|BROAD Institute lincRNA (XLOC_l2_01471	hs|chr9:15017475-15017534	A_21_P0013556	937	453E-02	-027	444E-02	621	-023	423E-01
ENST00000443359	ENST00000443359	Unknown	hs|chr9:18360655-18360596	A_21_P0006069	545	294E-02	-029	444E-02	568	012	639E-01
VLDLR-AS1	NR_015375	ref|Homo sapiens VLDLR antisense RNA 1 (VLD	hs|chr9:2535731-2535672	A_19_P00320469	554	174E-01	-018	444E-02	563	-001	973E-01
TOPORS-AS1	NR_033991	ref|Homo sapiens TOPORS antisense RNA 1 (T(	hs|chr9:32552870-32552929	A_24_P136641	919	962E-01	-001	444E-02	740	-010	762E-01
FAM74A1	NR_026803	ref|Homo sapiens family with sequence similar	hs|chr9:39900783-39900842	A_32_P57160	543	633E-01	-006	444E-02	553	018	444E-01
XLOC_l2_014820	TCONS_l2_00028765	linc|BROAD Institute lincRNA (XLOC_l2_01482(	hs|chr9:67282369-67288092	A_21_P0013572	557	350E-01	013	191E-02	578	000	995E-01
ENST00000448475	ENST00000448475	gb|BX096527 Soares_testis_NHT Homo sapien	hs|chr9:82489686-82489627	A_21_P0006082	544	173E-02	-031	444E-02	578	001	983E-01
lnc-FRMD3-1	lnc-FRMD3-1:1	linc|LNCipedia lincRNA (lnc-FRMD3-1), lincRNA	hs|chr9:86189005-86188946	A_21_P0006285	554	540E-01	008	191E-02	575	-065	232E-01
LINC01508	ENST00000436671	ens|long intergenic non-protein coding RNA 15	hs|chr9:93147336-93145983	A_21_P0006087	562	126E-02	033	191E-02	570	-009	757E-01
XLOC_l2_015593	TCONS_l2_00030324	linc|BROAD Institute lincRNA (XLOC_l2_01559	hs|chrX:106790446-106793311	A_21_P0013789	588	757E-02	024	326E-02	571	015	634E-01
LINC00633	NR_033941	ref|Homo sapiens long intergenic non-protein	hs|chrX:134253109-134253050	A_32_P110655	568	693E-02	024	191E-02	566	-002	950E-01
VENTXP1	NR_001559	ref|Homo sapiens VENT homeobox pseudogen	hs|chrX:26578376-26578435	A_24_P254762	520	146E-01	-020	444E-02	546	011	676E-01
lnc-MID1IP1-3	lnc-MID1IP1-3:1	linc|LNCipedia lincRNA (lnc-MID1IP1-3), lincRN	hs|chrX:38796696-38796755	A_21_P0006489	543	435E-01	-011	444E-02	572	009	754E-01
ENST00000438867	ENST00000438867	Unknown	hs|chrX:39260766-39260707	A_19_P00320780	634	178E-02	-031	444E-02	691	036	325E-01
UXT-AS1	NR_028119	ref|Homo sapiens UXT antisense RNA 1 (UXT-A	hs|chrX:47519412-47519471	A_33_P3382291	553	440E-01	-010	106E-02	569	-010	690E-01
LINC01496	ENST00000448761	ens|long intergenic non-protein coding RNA 14	hs|chrX:51245021-51244962	A_21_P0006450	536	177E-02	031	191E-02	557	-004	920E-01
XLOC_l2_015520	TCONS_l2_00030204	linc|BROAD Institute lincRNA (XLOC_l2_01552(	hs|chrX:52654718-52654777	A_21_P0013763	518	212E-01	-017	106E-02	558	-002	960E-01
XLOC_l2_015542	TCONS_l2_00030232	linc|BROAD Institute lincRNA (XLOC_l2_01554	hs|chrX:61999118-61999775	A_21_P0013768	758	115E-02	-033	106E-02	622	-028	222E-01
PABPC1L2B-AS1	NR_110398	ref|Homo sapiens PABPC1L2B antisense RNA 1	hs|chrX:72304415-72304474	A_21_P0006426	626	924E-01	-001	444E-02	572	011	684E-01
FTX	NR_028379	ref|Homo sapiens FTX transcript, XIST regulato	hs|chrX:73248632-73248573	A_21_P0000565	586	651E-01	006	106E-02	560	-009	743E-01
TTC3P1	NR_030737	ref|Homo sapiens tetratricopeptide repeat dor	hs|chrX:74960492-74960433	A_21_P0000586	755	648E-01	006	444E-02	646	021	425E-01
XLOC_l2_015766	TCONS_l2_00030544	linc|BROAD Institute lincRNA (XLOC_l2_01576(	hs|chrX:79526427-79526368	A_21_P0013822	562	258E-01	-015	444E-02	578	007	830E-01
lnc-KDM5D-4	lnc-KDM5D-4:1	linc|LNCipedia lincRNA (lnc-KDM5D-4), lincRNA	hs|chrY:22681903-22681844	A_21_P0006607	578	204E-01	017	191E-02	571	002	947E-01
lnc-ZFY-2	lnc-ZFY-2:1	linc|LNCipedia lincRNA (lnc-ZFY-2), lincRNA [lnc	hs|chrY:2981903-2981962	A_21_P0006599	527	525E-01	-009	387E-03	549	003	948E-01
TTTY1	NR_001538	ref|Homo sapiens testis-specific transcript, Y-li	hs|chrY:6269165-6269224	A_24_P323131	549	425E-04	046	191E-02	570	-001	987E-01
TTTY23	NR_001540	ref|Homo sapiens testis-specific transcript, Y-li	hs|chrY:9749473-9749532	A_24_P340227	512	785E-01	-004	444E-02	557	010	740E-01
LOC257396	NR_104654	ref|Homo sapiens uncharacterized LOC257396	unmapped	A_21_P0003999	573	232E-02	-030	444E-02	575	-005	864E-01
XLOC_l2_012210	TCONS_l2_00023087	linc|BROAD Institute lincRNA (XLOC_l2_01221(	unmapped	A_21_P0012925	653	341E-01	-013	444E-02	569	003	925E-01
