# Supplementary material for: LINC01116, a hypoxia‐lncRNA marker of pathological lymphangiogenesis and poor prognosis in lung adenocarcinoma
Source: Mol Oncol. 2025 Dec 9;20(4):962–80. doi: 10.1002/1878-0261.70175 (PMC13060655; doi:10.1002/1878-0261.70175)
Supplement: Supplementary file 2 — Fig. S1. Altering LINC01116 expression does not affect the proliferative and migratory properties of LUAD tumor cells. Fig. S2. LINC01116 CRISPRi efficiency in LUAD xenografts. Fig. S3. LINC01116 is expressed in tumor‐associated endothelial cells in LUADs. Fig. S4. Association between tumor stroma composition and LINC01116 expression in LUAD cohorts. Fig. S5. LINC01116 is expressed in lymphatic endothelial cells in the human lung. Fig. S6. Differentially Expressed Genes in LEC repressed for LINC01116 expression. [file MOL2-20-962-s001.pdf]

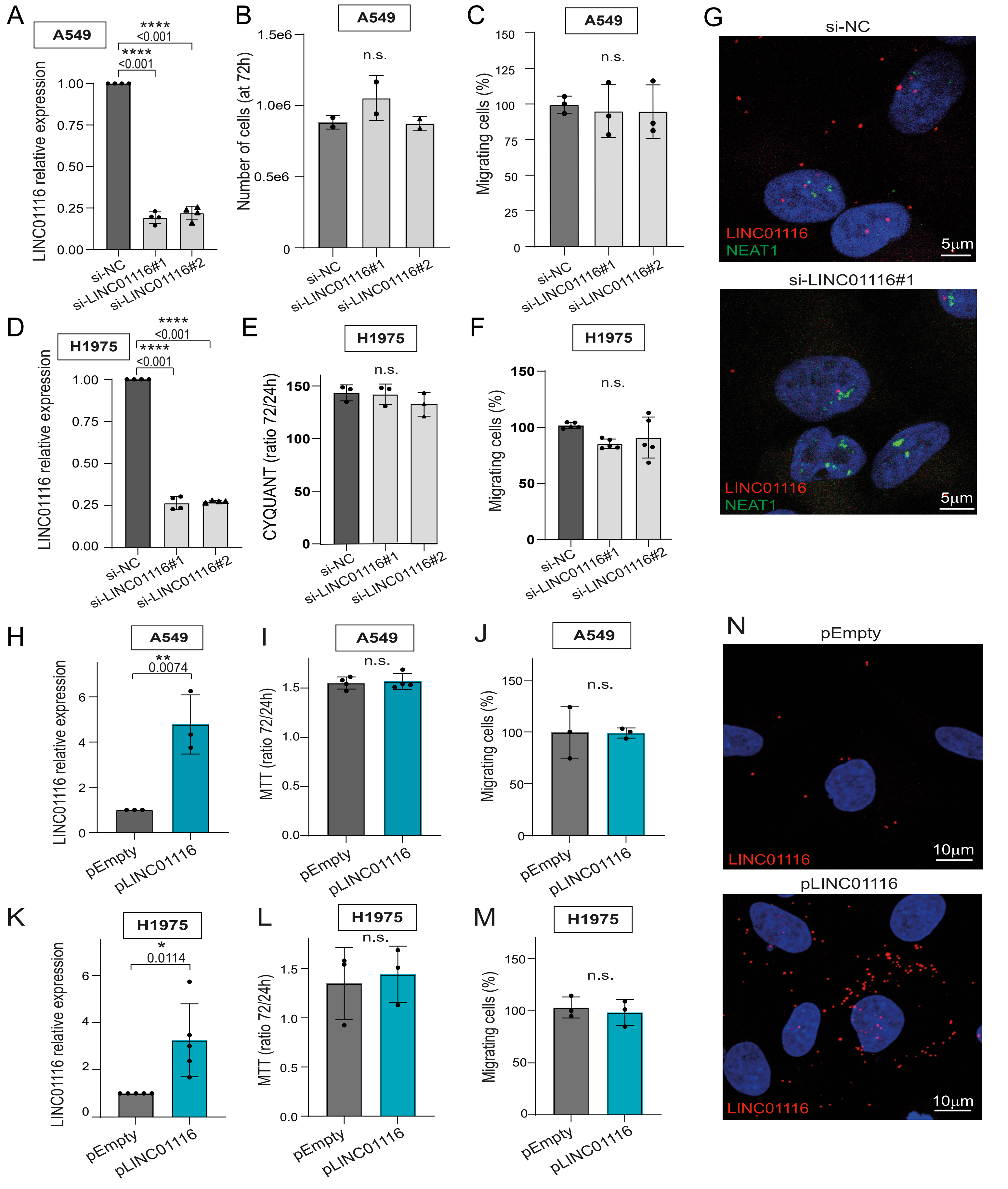

Supplemental Figure S1 – Altering LINC01116 expression does not affect the proliferative and migratory properties of LUAD tumor cells. A-G. Knock-down of LINC01116 was done in A549 and H1975 LUAD cells by the transient transfection of two distincts siRNA against LINC01116 (si-LINC01116#1 and #2) compared to a negative control siRNA (si-NC). **H-N**. Overexpression of LINC01116 was performed in the same cell lines through lentiviral transduction with pLINC01116 or an empty vector. **A,D,H,K**. LINC01116 expression was monitored by RT-qPCR. **G**. RNA-Fluorescence In Situ Hybridization (FISH) of LINC01116 (red) and NEAT1 (green) on A549 cells 48 hours after transfection with si-LINC01116#1 or si-NC. Nuclei were counterstained with DAPI (blue). Scale bar =5µm **B,E**. Cell proliferation assay: Cells were plated (250 000/well), transfected the next day with si-NC, si-LINC01116 #1 or #2 and cell proliferation assessed 48 hours later by cell counting (**B**) or CYQUANT assay (**E**). **C,F,J,M**. Cell migration assay: Cells were seeded in the upper chambers of Transwell inserts 48 hours after transfection with si-NC, si-LINC01116 #1 or #2. Then migrating cells were scored 24 hours later on the lower side of filters. **N**. RNA-FISH of LINC01116 (red) on pEmpty- and pLINC01116-stably transduced A549 cells. Nuclei were counterstained with DAPI (blue). Scale bar =10µm **I,L**. Cell proliferation was analyzed 72h after plating pEmpty- or pLINC01116-transduced cells using an MTT assay. **J,M**. Cell migration assay using boyden chambers on pEmpty- and pLINC01116-transduced cells. Data are the means ± SD of 2 independent experiments for **B**; 3 independent experiments for **C, E, H, J, L, M**; 4 independent experiments for **A, D, I**; 5 independent experiments for **F, K**. Statistical tests: (**A-F**) One-way ANOVA with pairwise comparison with Bonferroni correction and (**H-M**) Student's t test.

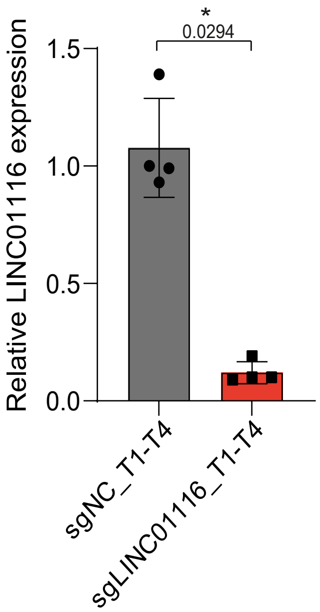

Supplemental Figure S2 – LINC01116 CRISPRi efficiency in LUAD xenografts.

Analysis of LINC01116 expression by qPCR in tumor xenografts from A549 cells transduced with single guide RNA (sgRNA) sgNC (n=4, T1-T4) or sgLINC01116 (n=4, T1-T4), at 46 days after engraftment. Data are the means ± SD. Statistical analysis was done using a Mann Whitney test.

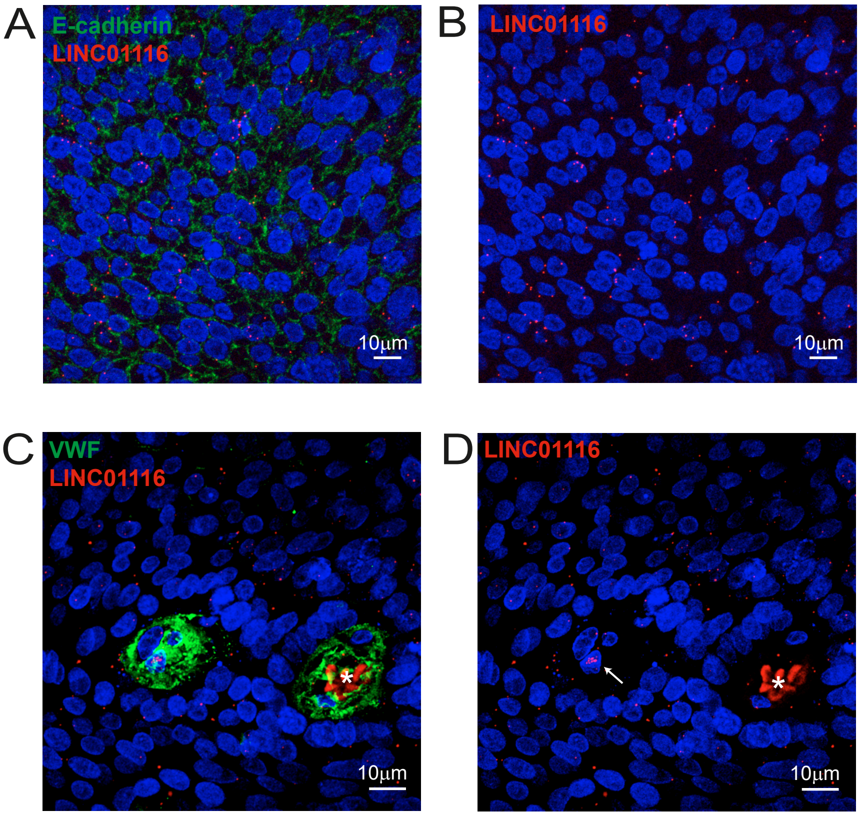

Supplemental Figure S3 – LINC01116 is expressed in tumor-associated endothelial cells in LUADs. Analysis of LINC01116 expression (Red) was done by RNA-Fluorescence In Situ Hybridization (FISH) on LUAD biopsy sections. Immunofluorescence co-labeling (Green) was performed with anti-E-cadherin (**A**) or anti-VWF (**C**) antibodies to label epithelial or endothelial cells respectively. E cadherin labeling, and to a lesser extent VWF labeling are altered by the RNA-FISH protocol protease treatment of tissue sections that allows access of oligo probes to target RNA. Nuclei were counterstained with DAPI. Asterisk shows unspecific trapping of fluorescence by red blood cells in a venous blood vessel.

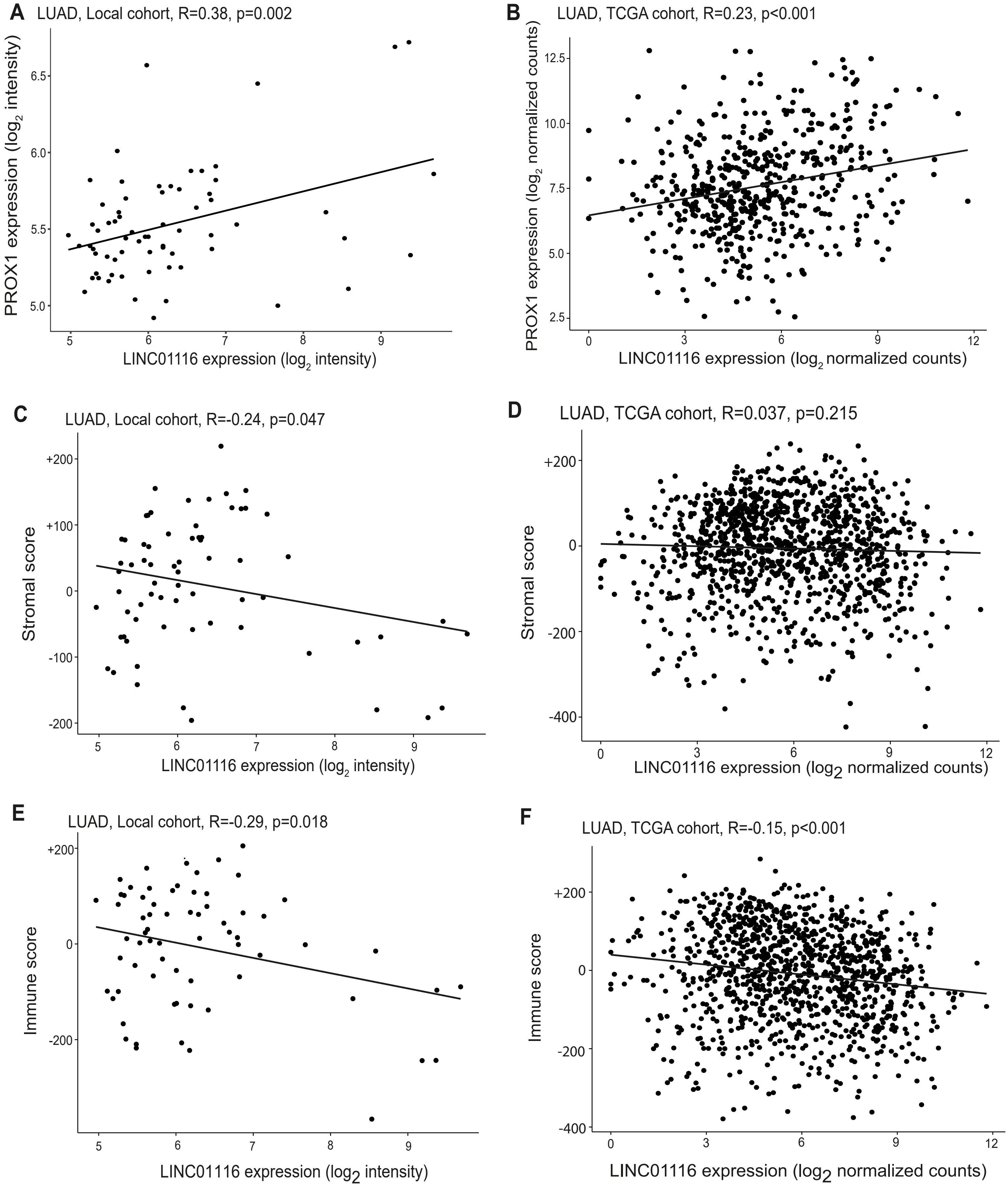

Supplemental Figure S4 – Association between tumor stroma composition and LINC01116 expression in LUAD cohorts.
Scatterplots between PROX1 expression (**A,B**), stromal score (**C,D**) or immune score (**E,F**) and LINC01116 expression in the local (**A,C,E**) (n=57) or in the TCGA (**B,D,F**) (n=531) LUAD data sets. R is the Pearson's correlation coefficient.

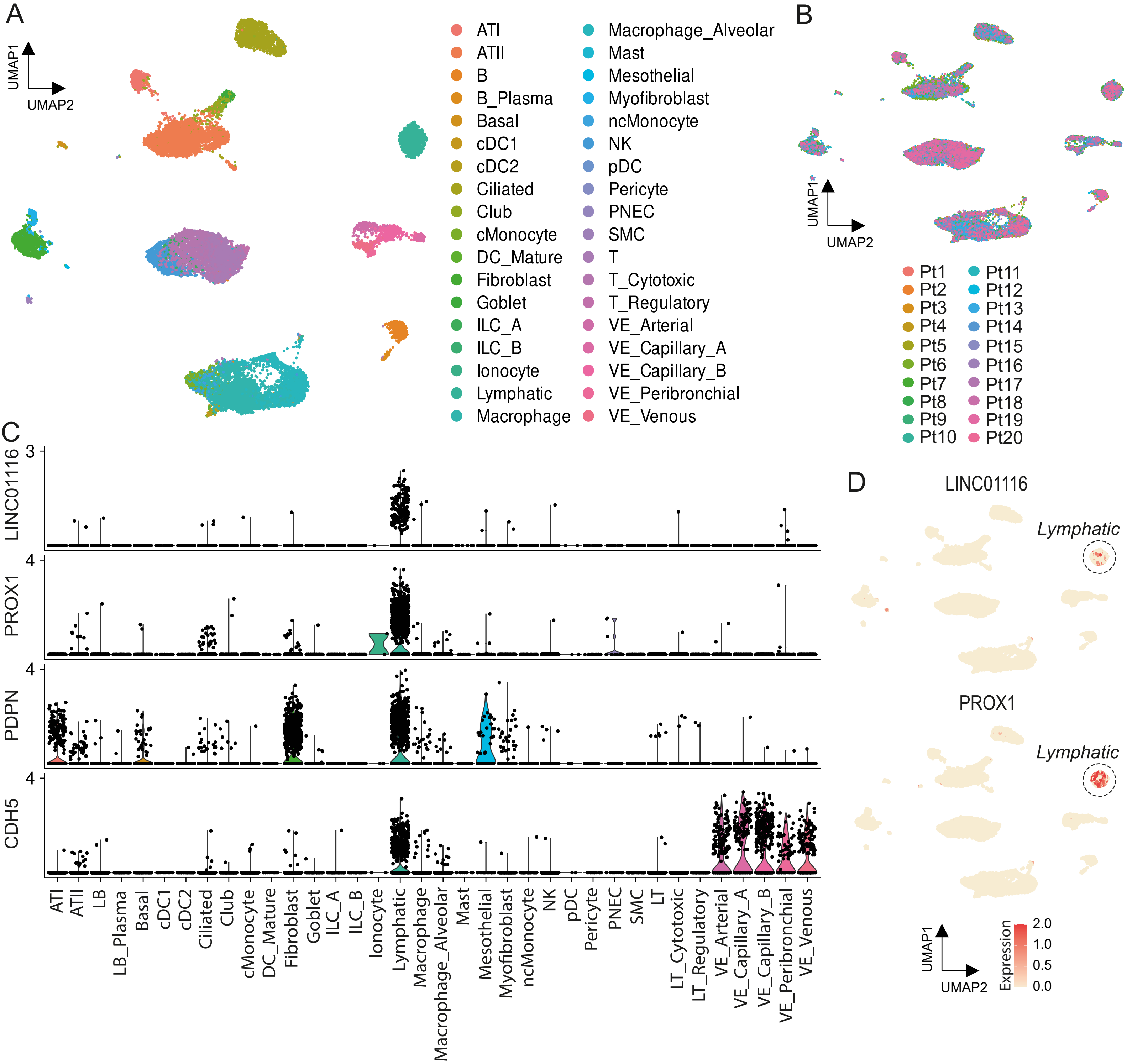

Supplemental Figure S5 – LINC01116 is expressed in lymphatic endothelial cells in the human lung.
A. Uniform manifold approximation and projection (UMAP) plots of healthy lungs scRNA-seq expression data from GSE136831 public dataset highlighting the main cell type clusters. **B.** UMAP plots of GSE136831 dataset colored by donors (Pt1-Pt20). **C.** Violin plots of normalized expression of LINC01116, and of PROX1, PDPN and CDH5, three markers of lymphatic endothelial cells, in each cell type clusters. **D.** UMAP plots of LINC01116 (Top) and PROX1 (Bottom) expression.

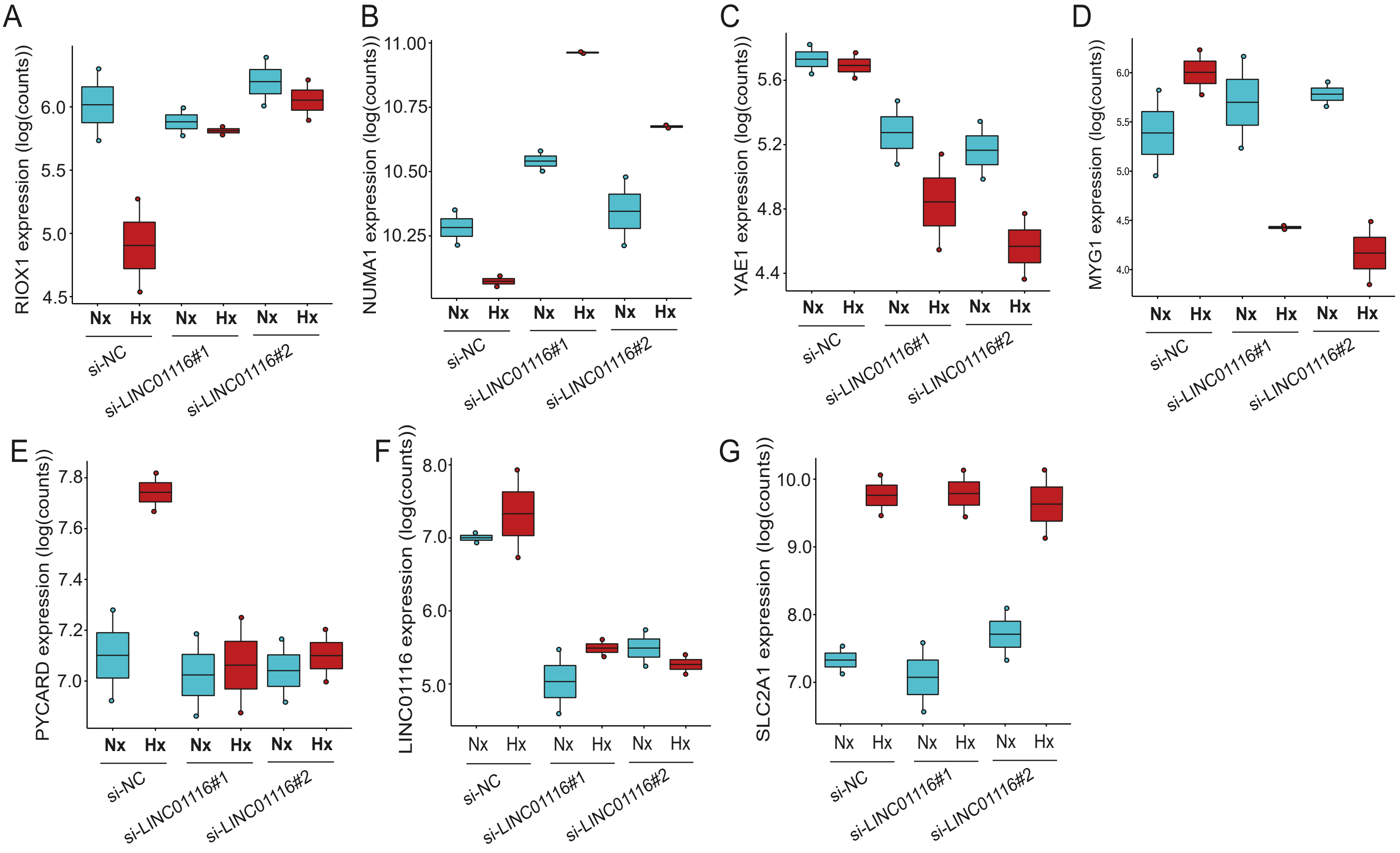

Supplemental Figure S6 – Differentially Expressed Genes in LEC repressed for LINC01116 expression.
A-G. Gene expression of RIOX1 (**A**), NUMA1 (**B**), YAE1 (**C**), MYG1 (**D**), PYCARD (**E**), LINC01116 (**F**) and SLC2A1 (**G**) in LEC transfected with a siRNA control (si-NC) or two distincts siRNAs against LINC01116 (si-LINC01116#1 and #2). LINC01116 (**F**) and SLC2A1 (**G**) gene expression highlight KD efficiency and hypoxic response, respectively. Box plots are medians ± quartiles of normalized expression from RNAseq data of two independent experiments on LEC cultured in normoxic (Nx) or hypoxic (Hx) conditions for 24 hours.
